# What Is the Evidence for Physical Therapy Poststroke? A Systematic Review and Meta-Analysis

**DOI:** 10.1371/journal.pone.0087987

**Published:** 2014-02-04

**Authors:** Janne Marieke Veerbeek, Erwin van Wegen, Roland van Peppen, Philip Jan van der Wees, Erik Hendriks, Marc Rietberg, Gert Kwakkel

**Affiliations:** 1 Department of Rehabilitation Medicine, MOVE Research Institute Amsterdam, VU University Medical Center, Amsterdam, The Netherlands; 2 Department of Physiotherapy, University of Applied Sciences Utrecht, Utrecht, The Netherlands; 3 Scientific Institute for Quality of Healthcare (IQ healthcare), Radboud University Nijmegen Medical Center, Nijmegen, The Netherlands; 4 Department of Epidemiology, Maastricht University, Maastricht, The Netherlands; 5 Department of Neurorehabilitation, Reade Center for Rehabilitation and Rheumatology, Amsterdam, The Netherlands; University of Glasgow, United Kingdom

## Abstract

**Background:**

Physical therapy (PT) is one of the key disciplines in interdisciplinary stroke rehabilitation. The aim of this systematic review was to provide an update of the evidence for stroke rehabilitation interventions in the domain of PT.

**Methods and Findings:**

Randomized controlled trials (RCTs) regarding PT in stroke rehabilitation were retrieved through a systematic search. Outcomes were classified according to the ICF. RCTs with a low risk of bias were quantitatively analyzed. Differences between phases poststroke were explored in subgroup analyses. A best evidence synthesis was performed for neurological treatment approaches. The search yielded 467 RCTs (N = 25373; median PEDro score 6 [IQR 5–7]), identifying 53 interventions. No adverse events were reported. Strong evidence was found for significant positive effects of 13 interventions related to gait, 11 interventions related to arm-hand activities, 1 intervention for ADL, and 3 interventions for physical fitness. Summary Effect Sizes (SESs) ranged from 0.17 (95%CI 0.03–0.70; I^2^ = 0%) for therapeutic positioning of the paretic arm to 2.47 (95%CI 0.84–4.11; I^2^ = 77%) for training of sitting balance. There is strong evidence that a higher dose of practice is better, with SESs ranging from 0.21 (95%CI 0.02–0.39; I^2^ = 6%) for motor function of the paretic arm to 0.61 (95%CI 0.41–0.82; I^2^ = 41%) for muscle strength of the paretic leg. Subgroup analyses yielded significant differences with respect to timing poststroke for 10 interventions. Neurological treatment approaches to training of body functions and activities showed equal or unfavorable effects when compared to other training interventions. Main limitations of the present review are not using individual patient data for meta-analyses and absence of correction for multiple testing.

**Conclusions:**

There is strong evidence for PT interventions favoring intensive high repetitive task-oriented and task-specific training in all phases poststroke. Effects are mostly restricted to the actually trained functions and activities. Suggestions for prioritizing PT stroke research are given.

## Introduction

Prospective studies have estimated that about 795.000 people in the USA suffer a first or recurrent stroke each year [1]. The prevalence of chronic stroke in the USA is estimated at about 7 million [1], with about 80% of patients with stroke being over the age of 65. The prevalence of stroke is likely to increase in the future due to the aging population. Even though acute stroke care has improved, for example by large-scale application of recombinant tissue plasminogen activator (rTPA) [1], [2] and organized interdisciplinary inpatient stroke care [3], and although mortality rates have been decreasing [1], a large number of patients still remain disabled regardless of the time that has elapsed poststroke. Only 12% of the patients with stroke are independent in basic activities of daily living (ADL) at the end of the first week [4]. In the long term, 25–74% of patients have to rely on human assistance for basic ADLs like feeding, self-care, and mobility [5].

Interdisciplinary complex rehabilitation interventions [6], [7] are assumed to represent the mainstay of poststroke care [8]. One of the key disciplines in interdisciplinary stroke rehabilitation is physical therapy which is primarily aimed at restoring and maintaining ADLs, usually starting within the first days and often continuing into the chronic phase poststroke [8]. While the interdisciplinary character of stroke rehabilitation is paramount, the availability of specific, up-to-date, and professional evidence-based guidelines for the physical therapy profession is crucial for making adequate evidence-based clinical decisions [9]–[11]. The recommendations in the first Dutch evidence-based ‘Clinical Practice Guideline for physical therapy in patients with stroke’ were based on meta-analyses of 123 randomized controlled trials (RCTs) and date back to 2004 [12]. In view of the tremendous growth in the number of RCTs in this field, it is now necessary to re-establish the “state of the art” concerning the evidence for physical therapy interventions in stroke rehabilitation. This aim is in line with the 2006 Helsingborg Declaration on European Stroke Strategies, which states that stroke rehabilitation should be based on evidence as much as possible [13], [14].

The first aim of the present systematic review was to update our previous meta-analyses of complex stroke rehabilitation interventions in the domain of physical therapy, based on RCTs with a low risk of bias (i.e. a moderate to good methodological quality) with no restrictions to the comparator. Primary outcomes, measured post intervention, were defined at the levels of body functions and/or activities and participation of the International Classification of Functioning, disability and health model (ICF) [15]. The second aim was to explore whether the timing of interventions poststroke moderated the main effects.

## Methods

### Definitions

In accordance with the definition used by the World Health Organization (WHO), stroke was defined as “rapidly developing clinical symptoms and/or signs of focal, and at times global, loss of cerebral function, with symptoms lasting more than 24 hours or leading to death, with no apparent cause other than that of vascular origin” [16]. We distinguished four poststroke phases: the hyper acute or acute phase (0–24 hours), the early rehabilitation phase (24 hours until 3 months), the late rehabilitation phase (3–6 months), and the chronic phase (>6 months).

A study was considered an RCT when “the individuals (or other units) followed in the trial were definitely or possibly assigned prospectively to one of two (or more) alternative forms of health care using random allocation” [17].

Physical therapy was defined as “therapeutic modalities frequently used in physical therapy specialty by physical therapists or physiotherapists to promote, maintain, or restore the physical and physiological well-being of an individual” (Medline Subject Heading; MeSH). According to the American Physical Therapy Association (APTA), “physical therapists are health care professionals who maintain, restore, and improve movement, activity, and health, enabling an individual to have optimal functioning and quality of life, while ensuring patient safety and applying evidence to provide efficient and effective care. Physical therapists evaluate, diagnose, and manage individuals of all ages who have impairments, activity limitations, and participation restrictions. In addition, physical therapists are involved in promoting health, wellness, and fitness through risk factor identification and the implementation of services to reduce risk, slow the progression of or prevent functional decline and disability, and enhance participation in chosen life situations.” [18].

Exercise therapy refers to “a regimen or plan of physical activities designed and prescribed for specific therapeutic goals” (MeSH) in the field of physical therapy, intended to restore optimal functioning [19]. For the present meta-analysis, we included the use of technical applications such as robotics, electrostimulation and treadmills with body-weight support.

In line with previous reviews, we defined intensity of practice as the number of hours spent in exercise therapy [12], [19], [20]. Treatment contrast refers to “the amount of time spent on exercise therapy by the experimental group minus that spent by the control group” [20].

Activities of daily living (ADL) are “the daily self-care activities required to function in the home and/or outdoor environment. They may be classified as basic or extended” [21]. Basic ADL covers the ability to perform basic activities of self-care and mobility [21], [22]. These activities are captured by a combination of two or more of the codes d510 (washing oneself), d530 (toileting), d550 (eating), d540 (dressing), b5253 (fecal continence) and b6202 (urinary continence), d410 (changing basic body position), d420 (transferring oneself), and d450 (walking) as listed in the ICF [22]. By contrast, extended ADL “whilst not fundamental to functioning, allow an individual to live independently, e.g. shopping, housekeeping, managing finances, preparing meals, and using transportation” [21].

### Study Identification

Our previous search, covering the period up to January 29, 2004, was updated. Relevant publications were identified by searching the electronic databases PubMed (last searched June 28, 2011), EBSCO*host*/Excerpta Medica Databank (EMBASE; last searched June 9, 2011), EBSCO*host*/Cumulative Index of Nursing and Allied Health Literature (CINAHL; last searched July 14, 2011), Wiley/Cochrane Library (Cochrane Database of Systematic Reviews [CDSR], Cochrane Central Register of Controlled Trials [CENTRAL], Cochrane Methodology Register [CMR], Database of Abstracts of Reviews of Effects [DARE], Health Technology Assessment Database [HTA], NHS Economic Evaluation Database [EED]; last searched July 21, 2011), Physiotherapy Evidence Database (PEDro; last searched August 24, 2011), and SPORTDiscus™ (last searched August 24, 2011). This was done by J.M.V. after two researchers (J.M.V. and J.C.F.K.) had built the search string. The databases were searched by indexing terms and free-text terms used with synonyms and related terms in the title or abstract. We searched for “stroke”, and “exercise” or “physical therapy” or “physiotherapy” or “rehabilitation”, and “randomized controlled trials” or “reviews” (see table 1). Additional searches were performed for specified interventions. The full search strategy can be obtained from the corresponding author. One reviewer (J.M.V.), who was not blinded, screened the titles and abstracts and assessed potentially relevant publications in full-text. In addition, references of included RCTs and relevant reviews like those of the Cochrane Collaboration and the Evidence-Based Review of Stroke Rehabilitation (EBRSR) were screened. Authors of conference abstracts were contacted for full-text publications, if available, and experts in the field were consulted.

**Table 1:** Search strategy PubMed.

#1	Search "Stroke"[Mesh] OR cva[tiab] OR cvas[tiab] OR poststroke*[tiab] OR stroke*[tiab] OR apoplex*[tiab]
#2	Search (((brain*[tiab] OR cerebr*[tiab] OR cerebell*[tiab] OR intracran*[tiab] OR intracerebral[tiab] OR vertebrobasilar[tiab]) AND vascular*[tiab]) OR cerebrovascular*[tiab]) AND (disease[tiab] OR diseases[tiab] OR accident*[tiab] OR disorder*[tiab])
#3	Search (brain*[tiab] OR cerebr*[tiab] OR cerebell*[tiab] OR intracran*[tiab] OR intracerebral[tiab] OR vertebrobasilar[tiab]) AND (haemorrhag*[tiab] OR hemorrhag*[tiab] OR ischemi*[tiab] OR ischaemi*[tiab] OR infarct*[tiab] OR haematoma*[tiab] or hematoma*[tiab] or bleed*[tiab])
#4	Search "Hemiplegia"[Mesh] OR "Paresis"[Mesh] OR (hemipleg*[tiab] OR hemipar*[tiab] OR paresis[tiab] OR paretic[tiab])
#5	Search #1 OR #2 OR #3 OR #4
#6	Search "Occupational Therapy"[MeSH] OR "Physical Therapy Modalities"[MeSH] OR "Rehabilitation"[MeSH] OR "Exercise Therapy"[Mesh] OR "Exercise Movement Techniques"[Mesh] OR "Physical Therapy (Specialty)"[MeSH] OR "Recovery of Function"[Mesh] OR "rehabilitation"[SH] OR rehabilitati*[tiab] OR physiotherap*[tiab] OR (physical[tiab] AND (therapy[tiab] OR therapies[tiab] OR activity[tiab] OR activities[tiab])) OR exercis*[tiab] OR training[tiab] OR (occupational[tiab] AND (therapy[tiab] OR therapies[tiab]))
#7	Search (review*[tiab] OR search*[tiab] OR survey*[tiab] OR handsearch*[tiab] OR hand-search*[tiab]) AND (databa*[tiab] OR data-ba*[tiab] OR bibliograph*[tiab] OR electronic*[tiab] OR medline*[tiab] OR pubmed*[tiab] OR embase*[tiab] OR Cochrane[tiab] OR cinahl[tiab] OR psycinfo[tiab] OR psychinfo[tiab] OR cinhal[tiab] OR "web of science"[tiab] OR "web of knowledge"[tiab] OR ebsco[tiab] OR ovid[tiab] OR mrct[tiab] OR metaregist*[tiab] OR meta-regist*[tiab] OR ((predetermined[tiab] OR pre-determined[tiab]) AND criteri*[tiab]) OR apprais*[tiab] OR inclusion criteri*[tiab] OR exclusion criteri*[tiab]) OR (review[pt] AND systemat*[tiab]) OR "systematic review"[tiab] OR "systematic literature"[tiab] OR "integrative review"[tiab] OR "integrative literature"[tiab] OR "evidence-based review"[tiab] OR "evidence-based overview"[tiab] OR "evidence-based literature"[tiab] OR "evidence-based survey"[tiab] OR "literature search"[tiab] OR ((systemat*[ti] OR evidence-based[ti]) AND (review*[ti] OR literature[ti] OR overview[ti] OR survey[ti])) OR "data synthesis"[tiab] OR "evidence synthesis"[tiab] OR "data extraction"[tiab] OR "data source"[tiab] OR "data sources"[tiab] OR "study selection"[tiab] OR "methodological quality"[tiab] OR "methodologic quality"[tiab] OR cochrane database syst rev[ta] OR meta-analy*[tiab] OR metaanaly*[tiab] OR metanaly*[tiab] OR meta-analysis[pt] OR meta-synthesis[tiab] OR metasynthesis[tiab] OR meta-study[tiab] OR metastudy[tiab] OR metaethnograph*[tiab] OR meta-ethnograph*[tiab] OR Technology Assessment, Biomedical[mh] OR hta[tiab] OR health technol assess [ta] OR evid rep technol assess summ[ta] OR health technology assessment[tiab]
#8	Search randomized controlled trial[pt] OR controlled clinical trial[pt] OR randomized[tiab] OR placebo[tiab] OR drug therapy[sh] OR randomly[tiab] OR trial[tiab] OR groups[tiab] OR "cross over"[tiab] OR "Cross-Over Studies"[Mesh]
#9	Search #7 OR #8
#10	Search #5 AND #6 AND #9 NOT (animal[mh] NOT human [mh])

Studies were included if they met the following inclusion criteria: (1) the study sample analyzed consisted exclusively of patients with stroke aged 18 years or over; (2) the study was designed as an RCT including those with a two-group parallel, multi-arm parallel, crossover, cluster, or factorial designs; (3) the experimental intervention delivered fitted the domain of physical therapy and aimed to improve body functions and/or activities and participation and/or contextual factors; (4) the comparator was usual care, another intervention, the same intervention with a different dose, or no intervention; (5) the outcomes were measured post intervention and belonged to the domain of physical therapy (see the section on “Intervention categories and outcome domains”); and (6) the full-text publication was written in English, French, German, Spanish, Portuguese, or Dutch.

A review protocol was not published. An ethics statement was not required for this work.

### Data Extraction

One reviewer (J.M.V.) extracted the following information from the included RCTs using two forms developed in advance: first author, year of publication, number of patients in each group, eligibility criteria, stroke characteristics including poststroke phase, intervention characteristics, outcome measures, timing of assessment, the authors’ conclusions and the post intervention, and if applicable follow-up, point measures and measures of variability for each of the reported outcomes. Study authors were contacted in case the published results could not be used in the meta-analyses, e.g. when ranges were given instead of standard deviations (SDs) or interquartile ranges (IQRs), or results were only presented in graphs. The extracted data for the meta-analyses were cross-checked in random order. Duplicate publications were included, but counted as one RCT.

### Intervention Categories and Outcome Domains

Based on consensus between the authors, physical therapy interventions for the rehabilitation of patients with stroke were divided into: (1) interventions related to gait and mobility-related functions and activities, including novel methods focusing on efficient resource use, such as circuit class training and caregiver-mediated exercises; (2) interventions related to arm-hand activities; (3) interventions related to activities of daily living; (4) interventions related to physical fitness; and (5) other interventions which could not be classified into one of the other categories. In addition, attention was paid to (6) intensity of practice and (7) neurological treatment approaches.

The ICF [15], [23] was used to classify the outcome measures into the following domains: **muscle and movement functions** (e.g. muscle power functions [b730], control of voluntary movement functions [b760], muscle tone functions [b735]), **joint and bone functions** (e.g. mobility of joint functions [b710]), **sensory functions** (e.g. proprioceptive function [b260], touch function [b365], sensory functions related to temperature and other stimuli [b720]), **gait pattern functions** [b770] (e.g. gait speed, stride length), **functions of the cardiovascular and respiratory systems** (e.g. heart functions [b410], blood pressure functions [b420], respiration functions [b440], respiratory muscle functions [b445], exercise tolerance functions [b455]), **mental functions** (e.g. quality of life, depression), **balance** (e.g. changing basic body position [d410], maintaining a body position [d415]), **walking** [d450] (e.g. distance, independence, falls), **arm-hand activities** (e.g. fine hand use [d440], hand and arm use [d445]), **basic ADL** (e.g. washing oneself [d510], toileting [d520], dressing [d540], eating [d550], urination functions [d620]), **extended ADL** (e.g. acquisition of goods and services [d620], preparing meals [d630], doing housework [d640], recreation and leisure [d920]), and **attitudes** (e.g. individual attitudes of immediate or extended family members, like caregiver strain [e410 and e425 respectively]). The primary outcomes were at the body functions and activities and participation levels, while secondary outcomes included contextual factors.

### Quality Appraisal

The PEDro checklist was used to assess the risk of bias in the included RCTs [24], [25]. This 11-item list estimates the internal and external validity of an RCT based on 11 items. The items concern eligibility criteria, random allocation, concealment of allocation, group similarity at baseline, blinding of subjects, blinding of therapists, blinding of assessors, availability of key outcome measures of more than 85% of the subjects, intention-to-treat analysis, between-group statistical comparisons, and point measures and measures of variability [24], [25]. Except for item 1, which assesses the generalizability, one point is awarded if a criterion is satisfied. The maximum score is 10 points. For the purpose of this study, we considered RCTs with a score of ≥4 to have a low risk of bias [12]. One reviewer (J.M.V.) scored all RCTs identified in the updated search unblinded and crosschecked the scores with the PEDro database (www.pedro.org.au). In case of disagreement, another reviewer (E.v.W) made the final decision. For RCTs not listed in the PEDro database, two reviewers (J.M.V. and E.v.W.) independently assessed the risk of bias and disagreements were resolved in a consensus meeting.

### Analyses

Data from identified RCTs are reported in the results section. Our quantitative analyses only included RCTs with a PEDro score of ≥4. Aggregated data of individual RCTs were pooled when at least two RCTs with a measure in the same outcome category were available for an intervention. Interventions for which pooling was possible were automatically indicated as “strong evidence”, regardless of the direction of the results, because only RCTs with a low risk of bias were included (Level 1) [26]. A “strong evidence” label was also assigned when only one phase III trial was available for a particular intervention. Analogous to our 2004 review, a qualitative analysis was performed for the intervention category “neurological treatment approaches”. Based on an adaptation of the criteria established by Van Tulder et al. [26] the following four levels of evidence were distinguished:

**Level 1.** Strong evidence – provided by generally consistent findings in multiple, relevant, high-quality RCTs.

**Level 2.** Moderate evidence – provided by findings in one relevant, high-quality RCT.

**Level 3.** Limited evidence – provided by generally consistent findings in one or more relevant low-quality RCTs.

**Level 4.** No or conflicting evidence – if there were no RCTs or if the results were conflicting.

RCTs with a PEDro score of ≥4 are considered to be of high-quality, while a score of <4 is considered as low-quality.

### Quantitative Analysis

Studies with a crossover design were considered RCTs. Measurements up to the crossover point were used as post intervention outcomes. Single-session experiments were not included in the quantitative analyses.

Meta-analyses were performed for each intervention for which at least two RCTs with comparable outcomes were identified. Based on post intervention outcomes (means and SDs), the individual effect sizes with their 95% confidence intervals (CI) were calculated as Hedges’ *g*. The individual Hedges’ *g* values were pooled to determine the summary effect size (SES; number of SD units) and 95%CI. The I^2^ statistic was used to determine statistical consistency (between-study variation) [17]. An I^2^ of >50.0% was considered to reflect substantial heterogeneity [17] and in that case a random-effects model was applied, while a fixed-effect model was applied in case of statistical homogeneity. A significant positive SES indicates that the experimental intervention is beneficial for patients when compared to a comparator. In the same vein, a significant negative SES indicates that the intervention has unfavorable effects for patients when compared to a comparator.

We pre-specified that in case of differences between RCTs in the timing of the interventions after stroke, a possible moderator effect of timing after stroke would be explored (in accordance with the phases described in the “*Definitions*” section) [27]. The variance between the subgroups was statistically tested in a “fixed-effect or random-effects within, fixed-effects between” model by applying the Q-test based on analysis of variance (ANOVA). Since the number of studies within each subgroup was five or less in nearly all meta-analyses, a pooled estimate of τ^2^ (variance of the distribution of the true effect sizes within subgroups) across subgroups was used, as separate estimates of τ^2^ for each subgroup are likely to be imprecise [27]. The SES (95%CI) and number of RCTs for each subgroup were only reported if there were significant differences between the poststroke phases.

In all analyses, the null hypothesis was rejected when the probability value was <0.05 (2-tailed). Following Cohen, the effect sizes were classified into small (<0.2), medium (0.2–0.8), and large (>0.8) [28]. All analyses were performed using Comprehensive Meta-analysis (Biostat, Englewood, New Jersey).

The statistical power of each meta-analysis was calculated post hoc, based on the number of RCTs included, the within-study sample size, the SES, the between-studies variance, and 2-tailed p-value [29]. A power of ≥0.8 was regarded as satisfactory.

## Results

### Study Identification

**Figure 1:**
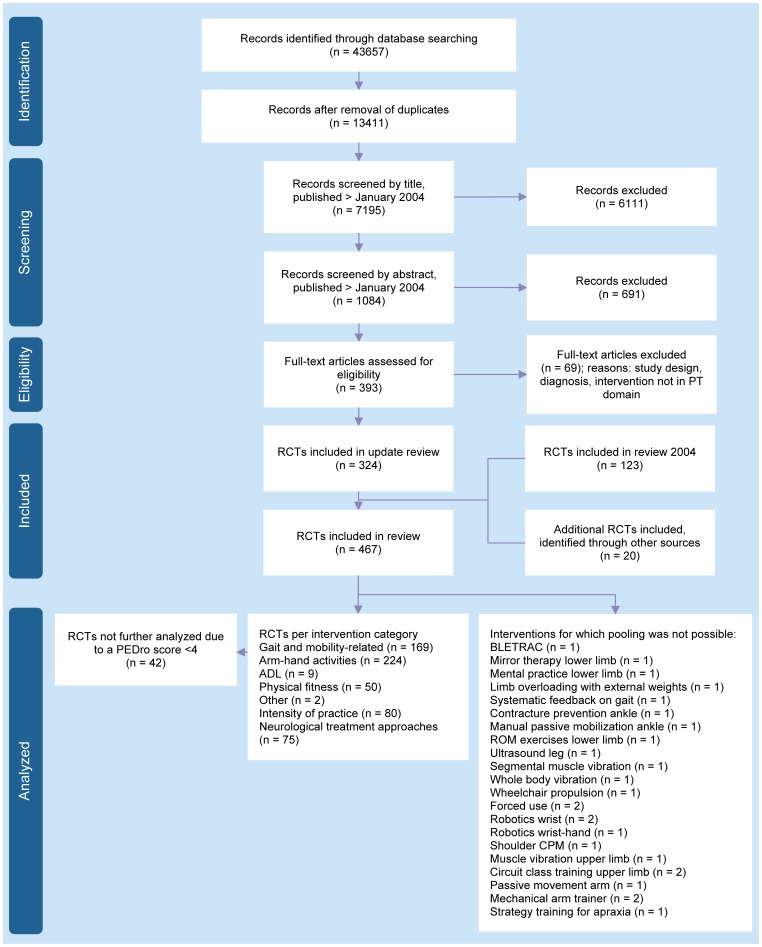
PRISMA Flow diagram. Legend: ADL, Activities of daily living; BLETRAC, Bilateral leg training with rhythmic auditory cueing; CPM, Continuous passive motion; PEDro, Physiotherapy evidence database; PT, Physical therapy; RCTs, Randomized controlled trials; ROM, Range of motion.

The search for relevant RCTs is visualized in figure 1. The final selection of RCTs consisted of 467 studies involving 25 373 patients with stroke; 123 RCTs from the 2004 search and an additional 344 RCTs from the updated search. Most studies included patients in the early rehabilitation phase (n = 198) or chronic phase (n = 202). Three RCTs included patients in the hyper acute or acute rehabilitation phase. For details see tables S1A–S1G in file S1.

### Quality Appraisal

The risk of bias in RCTs has decreased over time, as shown by the increase in PEDro scores from a median of 5 (IQR 4–6) points for RCTs published till 2004 [12] to 6 (IQR 5–7) for the RCTs published from 2004 to 2011. The median PEDro score of all 467 RCTs was 6 (IQR 5–7).

### Analyses

Pooling was possible for 23 physical therapy interventions related to gait and mobility-related functions and activities, for 23 interventions related to arm-hand activities, for two interventions related to ADL in general, for four interventions related to physical fitness, and for inspiratory muscle training which did not fit the other categories (see tables S1A–S1E in file S1). Meta-analyses were also performed for intensity of practice (for details see table S1F in file S1).

### Quantitative Analysis

**Figure 2:**
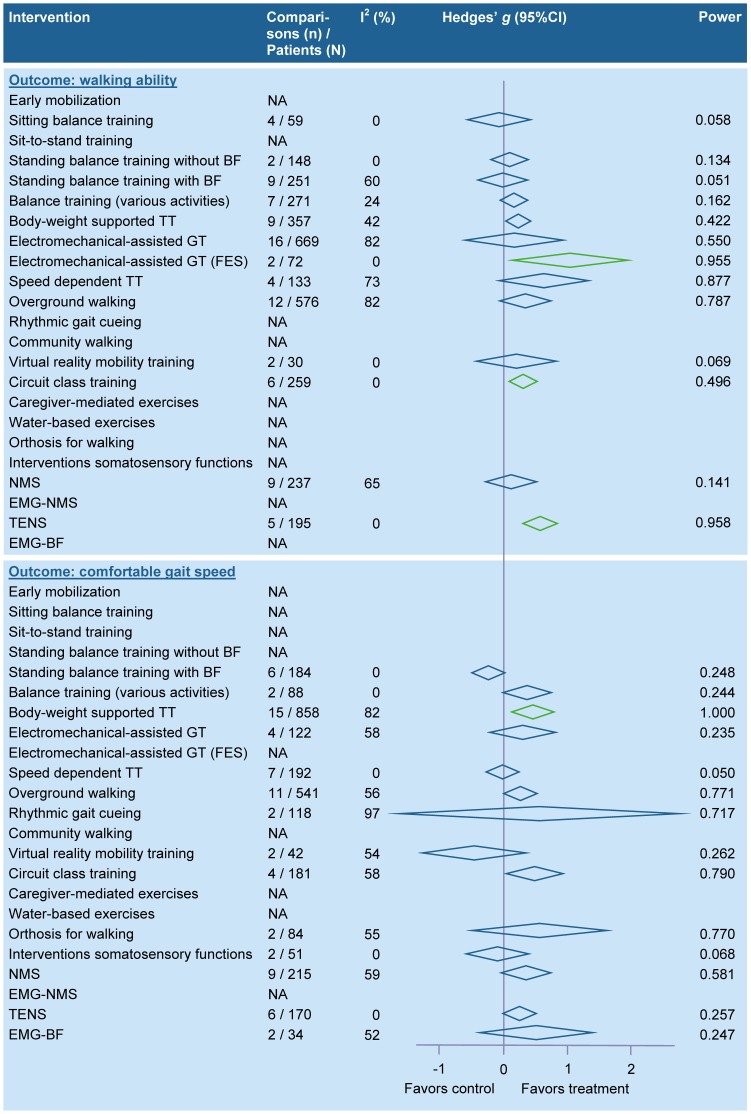
Summary effect sizes for physical therapy interventions – gait and mobility-related functions and activities. Legend: A green colored diamond indicates that the summary effect size is significant, while a blue colored diamond indicates that the summary effect size is nonsignificant; CI, Confidence interval; EMG-BF, Electromyographic biofeedback; EMG-NMS, Electromyography-triggered neuromuscular stimulation; FES, Functional electrostimulation; GT, Gait training; NA, Not applicable; NMS, Neuromuscular stimulation; TENS, Transcutaneous electrical nerve stimulation; TT, Treadmill training.

Physical therapy interventions related to gait and mobility-related functions and activities. The results of the meta-analyses for interventions related to gait and mobility-related functions and activities are summarized in figure 2 (for details see table S2A in file S1). Pooling was not possible for bilateral leg training with rhythmic gait cueing [30], mirror therapy for the paretic leg [31], mental practice with motor imagery [32], limb overloading with external weights [33], systematic verbal feedback on gait speed [34], maintenance of ankle dorsiflexion by using a standing frame or night splint [35], manual passive mobilization of the ankle [36], range of motion exercises of the ankle with specially designed equipment [37], ultrasound for the paretic leg [38], segmental muscle vibration for a drop foot [39], whole body vibration [40], and wheel chair propulsion [41].

1. Early mobilization

Early mobilization out of bed within 24 hours poststroke and stimulating the patient to exercise outside the bed [42] was investigated in two RCTs (N = 103, PEDro score 8) [43], [44], including patients in the hyper acute or acute phase.

A nonsignificant SES was found for complications, neurological deterioration early poststroke, fatigue, independence in basic ADL at 3 months, and discharge home.

2. Sitting balance training

Training of balance (i.e. maintaining, achieving, or restoring balance) during sitting [45] was investigated in six RCTs (N = 150, PEDro score range 4 [46] to 8 [47]) [46]–[51], including patients in the early rehabilitation phase [46], [47], [49]–[51] or chronic phase [48].

Overall, pooling of data showed a nonsignificant SES for symmetry while sitting and standing, balance, walking ability, and basic ADL. However, pooling only data of RCTs which investigated training of sitting balance while reaching beyond arm’s length yielded a significant heterogeneous positive SES for sitting balance. Nonsignificant SESs were found for ground reaction force while sitting and hand movement time. Subgroup analyses revealed no significant differences between poststroke phases.

3. Sit-to-stand training

Training the transfer from sit-to-stand and vice versa while maintaining balance [52] was investigated in five RCTs (N = 163, PEDro score range 4 [53] to 6 [54]–[56]) [53]–[57], including patients who were unable to perform a sit-to-stand without help in the early rehabilitation phase [53], [54], [56], [57] or chronic phase [55].

Nonsignificant SESs were found for body weight distribution, sit-to-stand, and balance. Subgroup analyses revealed no significant differences between poststroke phases.

4. Standing balance training without biofeedback

Training of balance (i.e. maintaining, achieving, or restoring balance) during standing [45] without the use of biofeedback was investigated in four RCTs (N = 199, PEDro score range 4 [58] to 8 [59]) [58]–[61], including patients in the early rehabilitation phase [59]–[61] or chronic phase [58]. The training consisted of standing on surfaces of different compliance with eyes open, optionally combined with eyes closed, or standing in a frame.

Nonsignificant SESs were found for postural sway, sit-to-stand, balance, and walking ability. Subgroup analyses revealed no significant differences between poststroke phases.

5. Standing balance training with biofeedback – force and position feedback

The use of a force platform with force sensors to measure the weight on each foot and the center of pressure to subsequently give visual or auditory feedback to the patient [8] was investigated in 12 RCTs (N = 333, PEDro score range 3 [62] to 6 [56], [63]–[67]) [56], [62]–[73], including patients in the early rehabilitation phase [56], [68]–[70], [72], [73], late rehabilitation phase [62]–[64], [67], [71], or chronic phase [66]. In most of the RCTs, patients had to be able to get from a seated to a standing position and be able to stand with or without physical support.

A significant homogeneous positive SES was found for postural sway. Subgroup analyses showed that the effect size was only significant in the chronic phase (n = 1), while the SES for the early rehabilitation phase (n = 6) was not. Nonsignificant SESs were found for motor function of the paretic leg (synergy), comfortable gait speed, step length, cadence, monopedal and bipedal phase, balance, walking ability, and basic ADL. Subgroup analyses revealed no significant differences between poststroke phases for these outcomes.

6. Balance training during various activities

Training of balance (i.e. maintaining, achieving, or restoring balance) during various activities [45] was investigated in 11 RCTs (N = 419, PEDro score range 4 [74] to 8 [75], [76]) [74]–[84], including patients in the early rehabilitation phase [76], [77], [80], [83], [84], late rehabilitation phase [74], [75], [82], or chronic phase [78], [79], [81].

Pooling resulted in a significant homogeneous positive SES for basic ADL and a significant heterogeneous positive SES for balance. Nonsignificant SESs were found for comfortable gait speed, falls-efficacy, walking ability, and quality of life. Subgroup analyses revealed no significant differences between poststroke phases.

7. Body-weight supported treadmill training

Treadmill training with the patient’s body-weight partially supported by a harness [8] was investigated in 18 RCTs (N = 1158, PEDro score range 4 [85]–[87] to 8 [88]–[91]) [85]–[105], including patients in the early rehabilitation phase [85]–[91], [94], [96], [98], [101], [103], [105] or chronic phase [90], [92], [93], [95], [97], [99], [100], [102], [104]. The patients had to be restricted in their walking ability, except in one study [90].

Meta-analyses showed significant heterogeneous positive SESs for comfortable gait speed and walking distance. Nonsignificant SESs were found for motor function of the paretic leg (synergy), maximum gait speed, stride length, cadence, aerobic capacity, energy expenditure, balance, walking ability, and quality of life. Subgroup analyses revealed no significant differences between poststroke phases.

8. Electromechanical-assisted gait training

Gait training using an apparatus which guides the walking cycle by electromechanical driven footplates or exoskeleton [8], [106], [107] was investigated in 16 RCTs (N = 766, PEDro score range 4 [108], [109] to 8 [110], [111]) [96], [102], [108]–[123], including patients in the early rehabilitation phase [96], [110], [113]–[115], [118]–[123], late rehabilitation phase [109], or chronic phase [102], [108], [112], [116]. For the purpose of this review, the meta-analyses for electromechanical-assisted gait training were subdivided into two groups: (a) without functional electrostimulation and (b) with functional electrostimulation.

a. Electromechanical-assisted gait training without functional electrostimulation

Electromechanical-assisted gait training without functional electrostimulation was investigated in 16 RCTs (N = 766) [96], [102], [108]–[110], [112]–[123].

Pooling resulted in significant homogeneous positive SESs for maximum gait speed, walking distance, peak heart rate, and basic ADL. Nonsignificant SESs were found for neurological functions, motor function of the paretic leg (synergy), muscle strength, comfortable gait speed, cadence, step length, heart rate at rest, balance, walking ability, extended ADL, and quality of life. Subgroup analyses showed significant differences between poststroke phases. The analysis for comfortable gait speed showed that only patients in the early rehabilitation phase who were dependent in walking benefited from electromechanical-assisted gait training. As regards balance, a significant homogeneous positive SES was found for the early rehabilitation phase (n = 4), a significant negative effect size for the late rehabilitation phase (n = 1), and a nonsignificant SES for the chronic phase (n = 4). As regards walking ability, a significant homogeneous positive SES was found for patients in the early rehabilitation phase (n = 12), a significant negative effect size for the late rehabilitation phase (n = 1), and a nonsignificant homogeneous negative SES for the chronic phase (n = 3).

b. Electromechanical-assisted gait training with functional electrostimulation

Electromechanical-assisted gait training with functional electrostimulation was investigated in three RCTs (N = 149) [112], [113], [118].

When data of these RCTs were pooled, significant homogeneous positive SESs were found for balance and walking ability (only for patients in the early rehabilitation phase). The statistical analyses for maximum gait speed and basic ADL resulted in nonsignificant SESs. Subgroup analyses for maximum gait speed revealed that patients in the early rehabilitation phase (dependent in walking; n = 1) significantly benefitted from electromechanical-assisted gait training with functional electrostimulation, while a nonsignificant effect was found for patients with chronic stroke (independent in walking; n = 1).

9. Speed dependent treadmill training (without body-weight support)

Speed dependent treadmill training without a harness to partially support the body-weight was investigated in 13 RCTs (N = 610, PEDro score range 4 [124], [125] to 8 [126], [127]) [92], [124]–[136], including patients in the early rehabilitation phase [127], [129], [136]; late rehabilitation phase [130], or chronic phase [92], [124]–[126], [128], [131], [132], [134], [135].

Pooling the results of individual RCTs showed significant homogeneous positive SESs for maximum gait speed and step width. For comfortable gait speed, gait speed endurance, stride length, cadence, VO_2_max, balance, and walking ability nonsignificant SESs were found. Subgroup analyses revealed no significant differences between poststroke phases.

10. Overground walking

Overground walking [137] was investigated in 19 RCTs (N = 1008, PEDro score range 2 [138] to 8 [89], [103], [139]–[143]) [86], [87], [89], [103], [109], [112], [119], [122], [123], [125], [138]–[150], including patients in the early rehabilitation phase [86], [89], [119], [122], [123], late rehabilitation phase [109], [140], [148], [150], or chronic phase [112], [125], [138], [139], [142], [144]–[147], [149].

The meta-analyses resulted in a significant homogeneous positive SES for anxiety in independently walking patients and a significant homogeneous negative SES for aerobic capacity in patients unable to walk dependently. Nonsignificant SESs were found for comfortable gait speed, maximum gait speed, walking distance, stride length, stride time, cadence, gait pattern symmetry, peak heart rate (patients unable to walk dependently), diastolic blood pressure (independently walking patients), systolic blood pressure (independent walking patients), balance, number of falls (independently walking patients), depression (independently walking patients), walking ability, and basic and extended ADL. Subgroup analyses revealed a significant difference in effects between poststroke phases for walking distance, cadence, stride length, balance, and walking ability. As regards walking distance, a significant homogeneous positive SES was found for independently walking patients in the chronic phase (n = 4) and a significant homogeneous negative SES for patients in the early rehabilitation phase who were unable to walk independently (n = 5). As regards cadence, a nonsignificant SES was found in the late rehabilitation phase (n = 2) and a significant negative effect size in the chronic phase (n = 1). As regards stride length, a nonsignificant effect size was found in the early rehabilitation phase, and a significant positive effect size was found in the late rehabilitation phase and chronic phase (all n = 1). As regards balance, a significant positive effect size was found in the late rehabilitation phase (n = 1) and a nonsignificant SES in the chronic phase (n = 4). As regards walking ability, a nonsignificant SES was found in the early rehabilitation phase (n = 6), a significant positive effect size in the late rehabilitation phase (n = 1), and a significant homogeneous positive SES in the chronic phase (n = 5).

11. Rhythmic gait cueing

Rhythmic auditory cueing to improve the gait pattern [8], [151] was investigated in six RCTs (N = 231, PEDro score range 3 [151]–[153] to 7 [154]) [151]–[156], including patients in the early rehabilitation phase [151], [153]–[155] or chronic phase [152], [156].

Only the RCTs including patients in the early rehabilitation phase could be pooled. Nonsignificant SESs were found for gait speed, cadence, stride length, and gait pattern symmetry.

12. Community walking

Training of walking in a community environment like a shopping mall or park [157] was investigated in three RCTs (N = 94, PEDro score range 6 [157], [158] to 8 [126]) [126], [157], [158], including patients in the early rehabilitation phase [157] or chronic phase [126], [158].

Pooling the data from the individual RCTs resulted in nonsignificant SESs for maximum gait speed, walking distance, and balance confidence. Subgroup analyses revealed no significant differences between poststroke phases.

13. Virtual reality mobility training

Training of mobility in a virtual environment using computer technology which enables patients to interact with this environment and receive feedback about the performance of movements and activities [159], [160] was investigated in six RCTs (N = 150, PEDro score range 5 [161], [162] to 7 [163]) [161]–[167], including patients in the early rehabilitation phase.

The meta-analyses showed nonsignificant SESs for comfortable gait speed, maximum gait speed, step length, and walking ability.

14. Circuit class training

Supervised circuit class training focused on gait and mobility-related functions and activities, in which patients train in groups in various work stations [168], [169], was investigated in eight RCTs (N = 359, PEDro score range 5 [146] to 8 [75], [142], [149], [170], [171]) [75], [81], [142], [143], [146], [170]–[173], including patients in the early rehabilitation phase [170], late rehabilitation phase [75], [171], [173], or chronic phase [81], [142], [146], [172].

Pooling resulted in significant homogeneous positive SESs for walking distance, balance, walking ability, and physical activity. Nonsignificant SESs were found for muscle strength, gait speed, self-efficacy, depression, number of falls, basic and extended ADL, and quality of life. Subgroup analyses revealed no significant differences between poststroke phases.

15. Caregiver-mediated exercises

Training of gait and mobility-related functions and activities with a caregiver under the auspices of a physical therapist [174] was investigated in three RCTs (N = 350, PEDro score range 4 [144] to 8 [174], [175]) [144], [174], [175], including patients in the early rehabilitation phase [174], [175] or chronic phase [144].

The meta-analyses resulted in significant homogeneous positive SESs for basic ADL and caregiver strain. A nonsignificant SES was found for extended ADL. Subgroup analyses revealed no significant differences between poststroke phases.

16. Orthosis for walking

The use of a splint or orthosis (ankle foot orthosis [AFO] or knee ankle foot orthosis [KEVO]) for walking was investigated in four RCTs (N = 137, PEDro score range 2 [176] to 7 [177]) [85], [176]–[178], which included patients in the early rehabilitation phase [85] or chronic phase [177], [178]. The poststroke phase was unclear for one RCT [176].

After pooling, a nonsignificant SES for comfortable gait speed was found when comparing walking with an orthosis with walking without an orthosis. Subgroup analyses revealed no significant differences between poststroke phases.

17. Water-based exercises

Water-based exercises are defined as “a therapy programme using the properties of water, designed by a suitably qualified physical therapist, to improve function, ideally in a purpose-built and suitably heated hydrotherapy pool” [179]. These exercises were investigated in three RCTs (N = 65, PEDro score range 5 [180], [181] to 6 [182]) [180]–[182], which all included patients in the chronic phase.

A significant homogeneous positive SES was found for muscle strength and a nonsignificant SES for balance.

18. Interventions for somatosensory functions of the paretic leg

Interventions designed to decrease or resolve impairments of the somatosensory functions of the paretic leg by e.g. electrostimulation or exposure to different stimuli such as texture, shape, temperature, or position [183], [184] were investigated in six RCTs (N = 151, PEDro score range 5 [185] to 8 [186]) [60], [185]–[189], including patients in the early rehabilitation phase [60], [187], [189], late rehabilitation phase [186], [188], or chronic phase [185].

The meta-analyses resulted in nonsignificant SESs for motor function of the paretic leg (synergy), gait speed, and balance. Subgroup analyses revealed no significant differences between poststroke phases.

19. Electrostimulation of the paretic leg

Electrostimulation of peripheral nerves and muscles with external electrodes [190] can be applied during training of activities, but also when just functions, like ankle dorsiflexion, are trained in a non-functional manner. For the purpose of this review, electrostimulation was divided into (a) neuromuscular stimulation (NMS); (b) electromyography-triggered neuromuscular stimulation (EMG-NMS); and (c) transcutaneous electrical nerve stimulation (TENS). Electrostimulation of the paretic leg was investigated in 26 RCTs (N = 814, PEDro score range 2 [176] to 8 [186], [191], [192]) [113], [118], [176], [186], [191]–[213], including patients in the early rehabilitation phase [113], [118], [192], [195], [196], [199]–[201], [203], [204], [206], [208], [212], late rehabilitation phase [186], [193], [197], [209], or chronic phase [194], [198], [202], [205], [207], [210], [213]. The RCT investigating the combination of EMG-NMS and NMS was not included in the meta-analyses [195]. The electrostimulation was not applied when outcomes were measured.

a. NMS

NMS of the paretic leg was investigated in 18 RCTs (N = 551) [113], [118], [176], [191]–[194], [196]–[198], [201]–[204], [206]–[208], [213].

Pooling resulted in significant homogeneous positive SESs for motor function of the paretic leg (synergy), muscle strength, and muscle tone. Nonsignificant SESs were found for active range of motion, gait speed, cadence, step and stride length, gait symmetry, balance, walking ability, and basic ADL. Subgroup analyses revealed no significant differences between poststroke phases.

b. EMG-NMS

EMG-NMS of the paretic leg was investigated in two RCTs (N = 68) [199], [209].

The meta-analyses resulted in nonsignificant SESs for muscle tone and basic ADL. Subgroup analyses revealed no significant differences between phases poststroke.

c. TENS

TENS of the paretic leg was investigated in five RCTs (N = 349) [186], [200], [205], [210]–[212].

Meta-analyses showed significant homogeneous positive SESs for muscle strength and walking ability, while nonsignificant SESs were found for muscle tone, active range of motion, gait speed, and walking distance. Subgroup analyses revealed no significant differences between poststroke phases.

20. Electromyographic biofeedback for the paretic leg

Electromyographic biofeedback (EMG-BF) involves registering the muscle activity by surface electrodes that are applied to the skin covering the muscles of interest [214], [215]. A biofeedback apparatus converts the recorded muscle activity (EMG) into visual or auditory information. EMG-BF for the paretic leg was investigated in 11 RCTs (N = 254, PEDro score range 2 [216] to 7 [217]) [152], [194], [216]–[224], including patients in the early rehabilitation phase [216], [219], [224] or chronic phase [152], [194], [217], [218], [220], [222], [223].

Pooling resulted in nonsignificant SESs for range of motion, gait speed, step and stride length, and EMG activity. Subgroup analyses revealed no significant differences between poststroke phases.

#### Physical therapy interventions related to arm-hand activities

**Figure 3:**
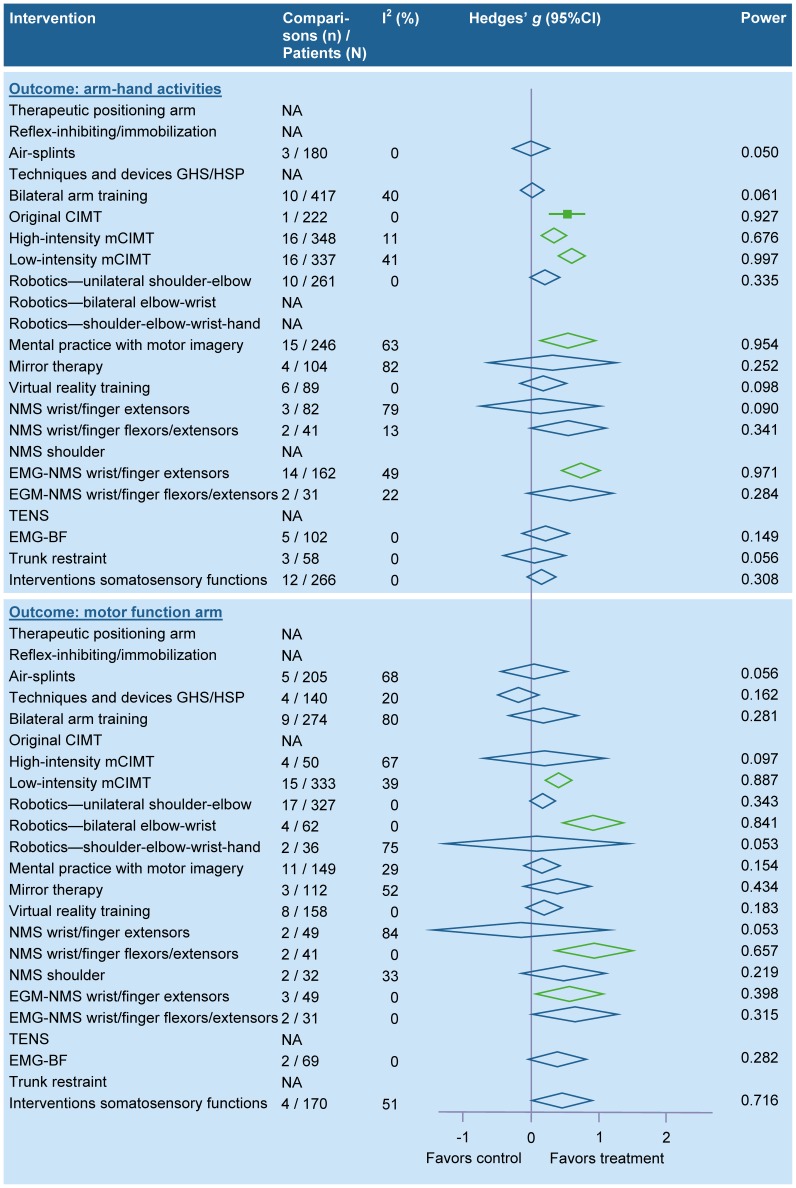
Summary effect sizes for physical therapy interventions – arm-hand activities. Legend: A green colored diamond indicates that the summary effect size is significant, while a blue colored diamond indicates that the summary effect size is nonsignificant; CI, Confidence Interval; CIMT, Constraint-induced movement therapy; EMG-BF, Electromyographic biofeedback; EMG-NMS, Electromyography-triggered neuromuscular stimulation; GHS, Glenohumeral subluxation; HSP, Hemiplegic shoulder pain; mCIMT, modified Constraint-induced movement therapy; NA, Not applicable; NMS, Neuromuscular stimulation; TENS, Transcutaneous electrical nerve stimulation.

The results of the meta-analyses for interventions related to arm-hand activities are summarized in figure 3 (for details see table S2B in file S1). Pooling was not possible for immobilization of the paretic arm (i.e. “forced-use”) [225], [226], wrist robotics [227], [228], wrist-hand robotics [229], continuous passive motion for the paretic shoulder [230], subsensory threshold electrical and vibration stimulation of the paretic arm [231], circuit class training [143], [182], passive bilateral arm training [232], and using a mechanical arm trainer [233], [234].

1. Therapeutic positioning of the paretic arm

Therapeutic positioning of the paretic arm, without the use of splints, with the purpose of maintaining range of motion and preventing harmful positions of the paretic arm [8] was investigated in five RCTs (N = 140, PEDro score range 6 [235], [236] to 7 [237]–[239]) [235]–[239], which all included patients in the early rehabilitation phase.

A significant homogeneous positive SES was found for passive range of motion of shoulder external rotation. Nonsignificant SESs were found for passive range of motion of shoulder internal rotation, external rotation contracture of the shoulder, pain at rest and while moving, and basic ADL.

2. Reflex-inhibiting positions and immobilization techniques for the paretic wrist and hand

The use of reflex-inhibiting positions or local immobilization of the wrist and hand by splints or plaster to (1) prevent or decrease an increased muscle tone or (2) to maintain or increase the range of motion of wrist and/or finger extension [8] were investigated in eight RCTs (N = 197, PEDro score range 3 [240] to 8 [241], [242]) [240]–[247], including patients in the early rehabilitation phase [241], [242], late rehabilitation phase [240], or chronic phase [243]–[247].

Meta-analyses resulted in nonsignificant SESs for passive range of motion, muscle tone, and pain. Subgroup analyses revealed no significant differences between poststroke phases.

3. Air-splints around the paretic arm

Air-splints give external pressure around the paretic limb and are primarily used to reduce an increased muscle tone [248], [249] and/or hand edema. Five RCTs investigated the effect of air-splints (N = 285, PEDro score range 4 [250], [251] to 8 [252]) [250]–[255], including patients in the early rehabilitation phase [250], [252], [254] or late rehabilitation phase [255]. The poststroke phase was unclear in one RCT [253].

Pooling resulted in nonsignificant SESs for motor function of the paretic arm (synergy), muscle tone, somatosensory functions, pain, and arm-hand activities. However, subgroup analyses revealed a significant homogeneous negative SES for muscle tone for patients in the early rehabilitation phase (n = 1, with 2 comparisons) and a significant homogeneous positive effect size for patients in the late rehabilitation phase (n = 1).

4. Supportive techniques or devices for the prevention or treatment of glenohumeral subluxation and/or hemiplegic shoulder pain

Supportive techniques – like strapping – or devices – like a sling or arm orthosis – for the prevention or treatment of glenohumeral subluxation and/or hemiplegic shoulder pain [256] were investigated in three RCTs (N = 142, PEDro score range from 4 [257] to 7 [258], [259]) [257]–[259], including patients in the early rehabilitation phase.

In the meta-analyses, nonsignificant SESs were found for motor function of the paretic arm and for pain.

5. Bilateral arm training

During bilateral arm training, movement patterns or activities are performed with both hands simultaneously but independent from each other and could be cyclic [8], [260]. This type of training was investigated in 22 RCTs (N = 823, PEDro score range 2 [261], [262] to 8 [263]) [261]–[282], including patients in the early rehabilitation phase [263], [265], [272], late rehabilitation phase [273], or chronic phase [261], [262], [264], [265], [267]–[271], [274]–[282]. The poststroke phase was unknown for one RCT [266].

The meta-analyses yielded nonsignificant SESs for motor function of the paretic arm (synergy), muscle strength, arm-hand activities, self-reported arm-hand use in daily life, and basic ADL. Subgroup analyses revealed no significant differences between poststroke phases.

6. Original or modified Constraint-induced movement therapy

Original or modified Constraint-Induced Movement Therapy (CIMT or mCIMT respectively) consists of immobilization of the non-paretic arm and is combined with repetitive task-specific training of the paretic arm, including shaping techniques [8].

(m)CIMT was investigated in 41 RCTs (N = 1342, PEDro score range 2 [261], [262], [283]–[285] to 8 [286]) [225], [226], [261], [262], [264], [270], [278], [282]–[318], including patients in the early rehabilitation phase [225], [226], [288], [293], [295], [299], [305], [309], [310], [312], [318], late rehabilitation phase [284], [289], [297], or chronic phase [261], [262], [264], [270], [278], [282], [283], [285]–[287], [290]–[292], [294], [296], [300]–[304], [307], [308], [313]–[317].

Different categories can be distinguished, depending on the duration of the immobilization of the paretic arm and the intensity of task-specific practice: (a) original CIMT, (b) high-intensity mCIMT, (c) low-intensity mCIMT, and (d) immobilization of the non-paretic arm (i.e. “forced-use”).

a. Original CIMT

Original CIMT is applied for 2 to 3 weeks and consists of (1) immobilization of the non-paretic arm with a padded mitt for 90% of the waking hours; (2) task-oriented training with a high number of repetitions for 6 hours a day; and (3) behavioral strategies to improve both compliance and transfer of the activities practiced from the clinical setting to the patient’s home environment. Original CIMT was investigated in one RCT (N = 222) [297], [298], which included patients in the late rehabilitation phase.

Significant positive effect sizes were found for arm-hand activities, self-reported amount of arm-hand use in daily life, and self-reported quality of arm-hand movement in daily life. Due to the size of the study sample and the low risk of bias, this result is classified as level 1 evidence.

b. High-intensity mCIMT

High-intensity mCIMT consists of (1) immobilization of the non-paretic arm with a padded mitt during 90% of the waking hours and (2) between 3 and 6 hours of task-oriented training a day. High-intensity mCIMT was investigated in 17 RCTs (N = 512) [261], [270], [285]–[287], [290], [291], [295], [296], [299], [304], [305], [308], [310]–[312], [314], [318], including patients in the early rehabilitation phase [295], [299], [305], [310], [312], [318] or chronic phase [261], [270], [285]–[287], [290], [291], [296], [304], [308], [314].

Pooling resulted in significant homogeneous positive SESs for arm-hand activities and self-reported quality of arm-hand movement in daily life. In addition, a significant heterogeneous positive SES was found for self-reported amount of the arm-hand use in daily life. Nonsignificant SESs were found for motor function of the paretic arm (synergy) and basic ADL. Subgroup analyses revealed a significant difference between poststroke phases for basic ADL. A significant positive effect size was found for the early rehabilitation phase (n = 1) and a nonsignificant effect size for the chronic phase (n = 1).

c. Low-intensity mCIMT

Low-intensity mCIMT consists of (1) immobilization of the non-paretic arm with a padded mitt during >0% to <90% of the waking hours and (2) between 0 and 3 hours of task-oriented training a day. Low-intensity mCIMT was investigated in 23 RCTs (N = 627) [262], [264], [278], [280], [282]–[284], [288], [289], [292]–[294], [300]–[303], [307], [309]–[313], [315], [317], including patients in the early rehabilitation phase [288], [293], [309], [312], late rehabilitation phase [284], [289], or chronic phase [262], [264], [278], [282], [283], [292], [294], [300]–[303], [307], [313], [315]–[317].

The meta-analyses yielded significant homogeneous positive SESs for motor function of the paretic arm (synergy), arm-hand activities, self-reported amount of arm-hand use in daily life, self-reported quality of arm-hand movement in daily life, and basic ADL. A nonsignificant SES was found for arm-related quality of life. Subgroup analyses for motor function of the paretic arm (synergy) showed that the positive effects were significant for the early rehabilitation phase (n = 1) and chronic phase (n = 12), but not for the late rehabilitation phase (n = 2).

7. Robot-assisted arm training

Robotic devices allow repetitive, interactive, high intensity training of the paretic arm and/or hand [8], [319]. Training with robotic devices was investigated in 22 RCTs (N = 648, PEDro score range 4 [227], [320]–[322] to 8 [323]) [227]–[229], [273], [320]–[338], including patients in the early rehabilitation phase [321], [322], [324], [325], [329], [331], [332], [336], [337], late rehabilitation phase [273], or chronic phase [227]–[229], [320], [323], [326]–[328], [330], [333]–[335], [338].

For the purpose of this review, robotic devices are classified on the basis of the joints they target: (a) shoulder-elbow robots; (b) elbow-wrist robots; and (c) shoulder-elbow-wrist-hand robots.

a. Shoulder-elbow robotics

Shoulder-elbow robots used in a unilateral mode were applied in 15 RCTs (N = 546) [273], [322], [324], [326]–[328], [330]–[338].

Pooling resulted in significant homogeneous positive SESs for motor function of the proximal part of the paretic arm (synergy), muscle strength, and pain. Nonsignificant SESs were found for motor function of the paretic arm, motor function of the distal part of the paretic arm, muscle tone, arm-hand activities, basic ADL, and quality of life. Subgroup analyses revealed no significant differences between poststroke phases.

b. Elbow-wrist robotics

Elbow-wrist robots used in a bilateral mode were investigated in two RCTs (N = 62) [323], [329].

Meta-analyses showed significant homogeneous positive SESs for motor function of the paretic arm (synergy) and muscle strength. Subgroup analyses revealed no significant differences between phases poststroke.

c. Shoulder-elbow-wrist-hand robotics

Shoulder-elbow-wrist-hand robots were investigated in two RCTs (N = 39) [320], [321].

Pooling the data resulted in nonsignificant SESs for both motor function of the paretic arm (synergy) and muscle strength of the distal part of the arm. Subgroup analyses revealed no significant differences between poststroke phases.

8. Mental practice with motor imagery

Mental practice of motor actions and/or activities for the purpose of improving their performance [8], [339] combined with physical practice, was investigated in 14 RCTs (N = 424, PEDro score range 4 [340], [341] to 7 [342]–[345]) [340]–[352], including patients in the early rehabilitation phase [340]–[342], [344], [345], [351] or chronic phase [346]–[350], [352], [353].

The meta-analyses showed a significant heterogeneous positive SES for arm-hand activities and nonsignificant SESs for motor function of the paretic arm (synergy), muscle strength, and basic ADL. Subgroup analyses revealed no significant differences between poststroke phases.

9. Mirror therapy for the paretic arm

During mirror therapy, the patient looks in a mirror placed perpendicular to the body. Looking in the mirror creates the suggestion that the patient is observing movements of the affected arm. Mirror therapy was investigated in seven RCTs (N = 255, PEDro score range 5 [349], [354] to 8 [355]) [349], [354]–[359], including patients in the early rehabilitation phase [359], late rehabilitation phase [357], [358], or chronic phase [349], [354]–[356].

Pooling resulted in nonsignificant SESs for motor function of the paretic arm (synergy), muscle tone, pain, and arm-hand activities. Subgroup analyses revealed a significant positive effect size for arm-hand activities in the late rehabilitation phase (n = 1) and a nonsignificant SES in the chronic phase (n = 2).

10. Virtual reality training for the paretic arm

Training of the arm and hand in a virtual environment using computer technology which enables patients to interact with this environment and receive feedback about the performance of movements and activities [159], [360] was investigated in 15 RCTs (N = 357, PEDro score range 3 [361]–[365] to 8 [366]) [360]–[375], including patients in the early rehabilitation phase [360], [363], [364], [373], [375], late rehabilitation phase [369], [370], or chronic phase [361], [362], [365]–[368], [371], [372], [374].

Pooling resulted in a significant homogeneous positive SES for basic ADL and a significant homogeneous negative SES for muscle tone. Nonsignificant SESs were found for motor function of the paretic arm (synergy) and arm-hand activities. Subgroup analyses revealed no significant differences between poststroke phases.

11. Electrostimulation of the paretic arm

Electrostimulation of peripheral nerves and muscles with external electrodes [190] can be applied during training of activities, but also when just functions, like wrist extension, are trained in a non-functional manner. For the purpose of the present review, electrostimulation was divided into (a) neuromuscular stimulation (NMS); (b) electromyography-triggered neuromuscular stimulation (EMG-NMS); and (c) transcutaneous electrical nerve stimulation (TENS). Electrostimulation of the paretic arm was investigated in 49 RCTs (N = 1521, PEDro score range 3 [376]–[379] to 8 [380]) [200], [267], [271], [321], [328], [376]–[423], including patients in the early rehabilitation phase [200], [321], [376], [380], [381], [383], [384], [386], [387], [389]–[392], [395], [402], [404], [405], [407], [413], [415]–[417], [419], [420], [422], late rehabilitation phase [382], [398]–[400], [406], [418], or chronic phase [267], [271], [328], [377]–[379], [393], [394], [396], [397], [401], [403], [408]–[412], [414], [421], [423]. The electrostimulation was not applied when outcomes were measured.

a. NMS

NMS of the paretic arm was investigated in 22 RCTs (N = 894) [376], [380], [381], [383]–[386], [389]–[392], [396], [398], [400], [402], [404], [406], [407], [410], [417]–[421].

a1. Wrist and finger extensors

Meta-analyses showed nonsignificant SESs for motor function of the paretic arm (synergy), active range of motion, muscle strength, and arm-hand activities. Subgroup analyses revealed no significant differences between poststroke phases.

a2. Wrist and finger flexors and extensors

The meta-analyses yielded significant homogeneous positive SESs for motor function of the paretic arm (synergy) and muscle strength, while the SES for arm-hand activities was nonsignificant.

a3. Shoulder muscles

Pooling resulted in a significant heterogeneous positive SES for shoulder subluxation, while nonsignificant SESs were found for motor function of the paretic arm (synergy), range of motion, and pain. Subgroup analyses revealed no significant differences between poststroke phases.

b. EMG-NMS

EMG-NMS of the paretic arm was investigated in 25 RCTs (N = 492) [267], [271], [321], [328], [378], [379], [387], [393]–[395], [397], [399], [401], [403]–[405], [408]–[414], [416], [422], [423].

b1. Wrist and finger extensors

The meta-analyses resulted in significant homogeneous positive SESs for motor function of the paretic arm (synergy) and arm-hand activities. A significant heterogeneous positive SES was found for active range of motion. The SESs for muscle strength and muscle tone were nonsignificant. Subgroup analyses revealed no significant differences between poststroke phases.

b2. Wrist and finger flexors and extensors

Pooling showed nonsignificant SESs for motor function of the paretic arm (synergy) and arm-hand activities. Subgroup analyses revealed no significant differences between poststroke phases.

c. TENS

TENS of the paretic arm was investigated in four RCTs (N = 484) [200], [377], [382], [388], [415].

Pooling resulted in nonsignificant SESs for both muscle tone and basic ADL. Subgroup analyses revealed no significant differences between poststroke phases.

12. Electromyographic biofeedback of the paretic arm

Electromyographic biofeedback (EMG-BF) involves the muscle activity being registered by surface electrodes which are applied to the skin covering the muscles of interest [214], [215]. A biofeedback apparatus converts the recorded muscle activity (EMG) into visual or auditory information. EMG-BF for the paretic arm was investigated in 11 RCTs (N = 317, PEDro score range 2 [424] to 7 [425], [426]) [219], [424]–[433], including patients in the early rehabilitation phase [219], [425], [430], late rehabilitation phase [426], [429], [432], [433], or chronic phase [427], [428], [431]. The phase poststroke was unclear for one RCT [424].

Meta-analyses resulted in nonsignificant SESs for motor function of the paretic arm (synergy), active range of motion, and arm-hand activities. Subgroup analyses revealed no significant differences between poststroke phases.

13. Trunk restraint

Fixing the trunk externally during reaching and grasping prevents compensatory movements of the trunk [434]. Trunk restraint was investigated in four RCTs (N = 86, PEDro score range 4 [435] to 8 [436]) [314], [434]–[436], which all included patients in the chronic phase.

The meta-analyses showed a significant homogeneous negative SES for self-reported amount of arm-hand use in daily life. A nonsignificant SES was found for active range of motion and arm-hand activities.

14. Interventions for somatosensory functions of the paretic arm

Interventions designed to decrease or resolve impairments in somatosensory functions of the paretic arm by e.g. electrostimulation or exposure to different stimuli like texture, shape, temperature or position [183], [184] were investigated in 12 RCTs (N = 580, PEDro score range 3 [377], [388] to 9 [437]) [188], [250], [251], [255], [377], [388], [398], [437]–[443], including patients in the early rehabilitation phase [250], [440], [443], late rehabilitation phase [188], [255], [398], [437], or chronic phase [377], [438], [439], [441], [442].

Meta-analyses showed significant homogeneous positive SESs for somatosensory functions and muscle tone. The analyses resulted in nonsignificant SESs for motor function of the paretic arm (synergy), muscle strength, pain, arm-hand activities, and basic ADL. Subgroup analyses revealed no significant differences between poststroke phases.

#### Physical therapy interventions for physical fitness

**Figure 4:**
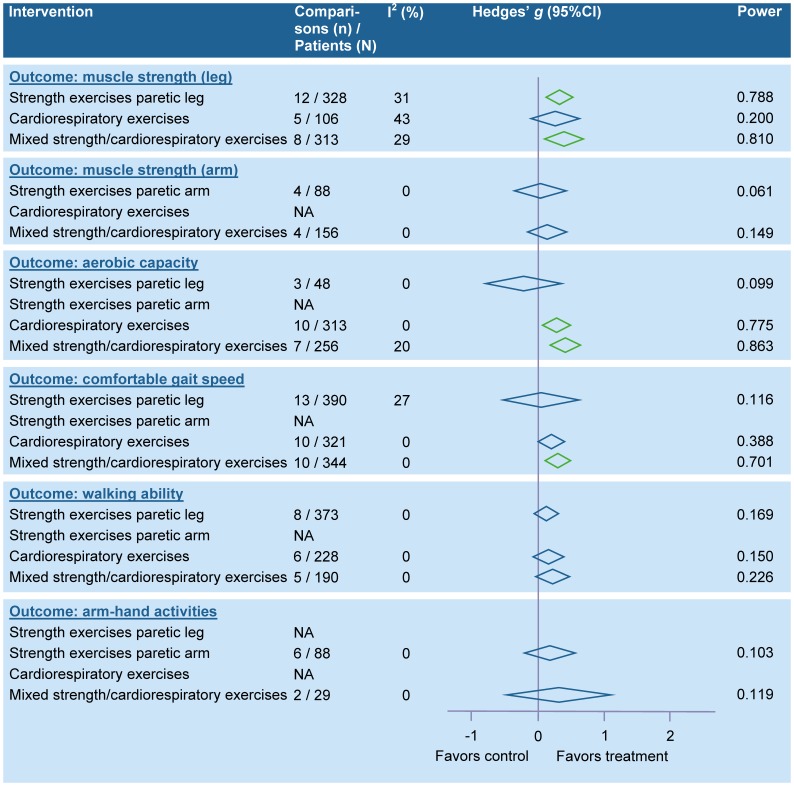
Summary effect sizes for physical therapy interventions – physical fitness. Legend: A green colored diamond indicates that the summary effect size is significant, while a blue colored diamond indicates that the summary effect size is nonsignificant; CI, Confidence interval; NA, Not applicable.

Planned and structured physical exercises aiming to improve physical fitness can be divided into programs primarily targeting (1) strength of the paretic leg; (2) strength of the paretic arm; (3) aerobic capacity; and (4) a combination of strength and aerobic capacity [8], [444], [445]. The results of the meta-analyses are summarized in figure 4 (for details see table S2C in file S1).

1. Strength exercises for the paretic leg

Progressive active exercises against resistance for the paretic leg were investigated in 19 RCTs (N = 786, PEDro score range 2 [446] to 8 [172], [447]) [172], [446]–[464], including patients in the early rehabilitation phase [448], [452], [456], [457], [461], [463], [464], late rehabilitation phase [449], or chronic phase [172], [446], [447], [450], [451], [453]–[455], [458], [460], [462].

Pooling resulted in significant homogeneous positive SESs for muscle strength, muscle tone, and spatiotemporal gait pattern parameters like cadence, stride length, and symmetry. Nonsignificant SESs were found for motor function of the paretic leg (synergy), comfortable gait speed, maximum gait speed, walking distance, aerobic capacity, heart rate work, workload, physical cost index, walking ability, basic ADL, and quality of life. Subgroup analyses revealed no significant differences between poststroke phases.

2. Strength exercises for the paretic arm

Progressive active exercises against resistance for the paretic arm were investigated in nine RCTs (N = 327, PEDro score range 2 [465] to 7 [99], [466]) [99], [446], [451], [462], [465]–[469], including patients in the early rehabilitation phase [465], [466], [468] or chronic phase [99], [446], [451], [462], [467], [469].

Pooling the data resulted in nonsignificant SESs for motor function of the paretic arm (synergy), muscle strength, range of motion, and pain. Subgroup analyses revealed no significant differences between poststroke phases.

3. Cardiorespiratory exercises

Interventions focusing on maintenance or improvement of the aerobic capacity by training large muscle groups, for example while walking overground or on a treadmill, or cycling on an ergometer, were investigated in 13 RCTs (N = 531, PEDro score range 4 [470], [471] to 8 [88], [127], [447], [459]) [88], [104], [124], [127], [132]–[135], [182], [447], [459], [470]–[477], including patients in the early rehabilitation phase [88], [127], [472], [477] or chronic phase [104], [132], [182], [447], [470], [471], [474]–[476].

Pooling resulted in significant homogeneous positive SESs for aerobic capacity and workload, and significant heterogeneous positive SESs for respiratory functions such as forced expiratory volume in 1 second (FEV_1_). Nonsignificant SESs were found for motor function of the paretic leg (synergy), muscle strength, comfortable gait speed, maximum gait speed, heart rate at rest and during work, diastolic and systolic blood pressure, physical cost index, body composition, blood variables, sitting and standing balance, and walking ability. Subgroup analyses showed significant differences between poststroke phases for resting heart rate: a significant SES was found for the early rehabilitation phase (n = 2) and a nonsignificant SES for the chronic phase (n = 2).

4. Mixed strength and cardiorespiratory exercises

Training regimes which combined both strength and cardiorespiratory exercises were investigated in 13 RCTs (N = 608, PEDro score range 3 [478] to 8 [140], [447], [479]) [140], [142], [143], [146], [171], [447], [459], [478]–[487], including patients in the early rehabilitation phase [479]–[481], [486], [487], late rehabilitation phase [140], [171], or chronic phase [142], [146], [447], [478], [482], [485].

Significant homogeneous positive SESs were found for motor function of the paretic leg (synergy), muscle strength of the leg, comfortable gait speed, maximum gait speed, walking distance, aerobic capacity, heart rate during work, balance, physical activity, and quality of life. Nonsignificant SESs were found for motor function of the paretic arm (synergy), muscle strength of the arm, physical cost index, depression, walking ability, arm-hand activities, and basic and extended ADL. Subgroup analyses revealed no significant differences between poststroke phases.

#### Physical therapy interventions related to activities of daily living

**Figure 5:**
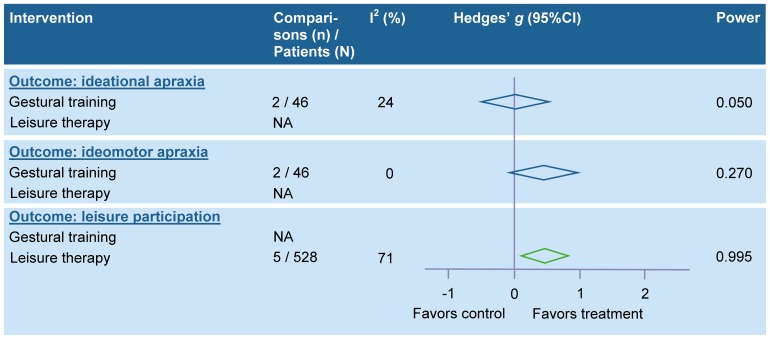
Summary effect sizes for physical therapy interventions – activities of daily living. Legend: A green colored diamond indicates that the summary effect size is significant, while a blue colored diamond indicates that the summary effect size is nonsignificant; CI, Confidence interval; NA, Not applicable.

The results of the meta-analyses for interventions related to activities of daily living are summarized in figure 5 (for details see table S2D in file S1). Pooling was not possible for strategy training for apraxia [488].

1. Interventions for apraxia: gestural training

Gestural training has been developed for patients with apraxia to teach them to regain tasks and handling of objects by using gestures [489]. This training method was investigated in two RCTs (N = 46) [489], [490], including patients in the chronic phase.

Pooling showed a significant homogeneous positive SES for gesture comprehension. Nonsignificant SESs were found for ideational and ideomotor apraxia.

2. Leisure therapy

Leisure therapy focuses on the execution of individual and social activities at home or in the home environment [491], [492]. This therapy was investigated in five RCTs (N = 641) [491]–[496], including patients who were to be discharged home or were already living at home in the early rehabilitation phase [492], [495], late rehabilitation phase [494], or chronic phase [491], [496].

The meta-analyses resulted in a significant heterogeneous positive SES for participation in leisure activities, while nonsignificant SESs were found for depression, mood, and quality of life. Subgroup analyses revealed significant differences between groups for participation in leisure activities: there was a significant homogeneous positive SES for the early rehabilitation phase (n = 1, with 2 comparisons), a nonsignificant SES size for the late rehabilitation phase (n = 1, with 2 comparisons), and a nonsignificant effect size for the chronic phase (n = 1).

#### Other physical therapy interventions

**Figure 6:**
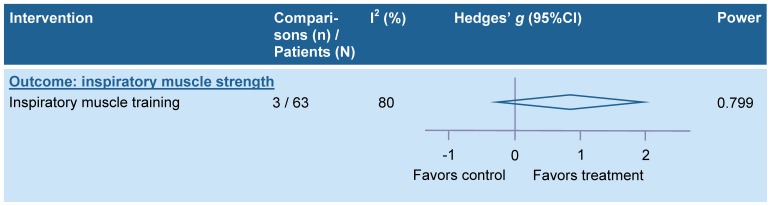
Summary effect sizes for physical therapy interventions – other: inspiratory muscle training. Legend: C, Control group; CI, Confidence interval; E, Experimental group.

The results of the meta-analyses for other physical therapy interventions are summarized in figure 6 (for details see table S2E in file S1).

1. Inspiratory muscle training

Inspiratory muscle training was investigated in two RCTs (N = 66, PEDro score range 4 [497] to 7 [498]) [497], [498], including patients in the late rehabilitation phase [498] or chronic phase [497].

Pooling was possible for maximal inspiratory pressure, which resulted in a nonsignificant SES. Subgroup analyses revealed a difference between poststroke phases. A significant positive effect size was found in the chronic phase (n = 1) and a nonsignificant SES in the late rehabilitation phase (n = 1, with 2 comparisons).

#### Intensity of practice

**Figure 7:**
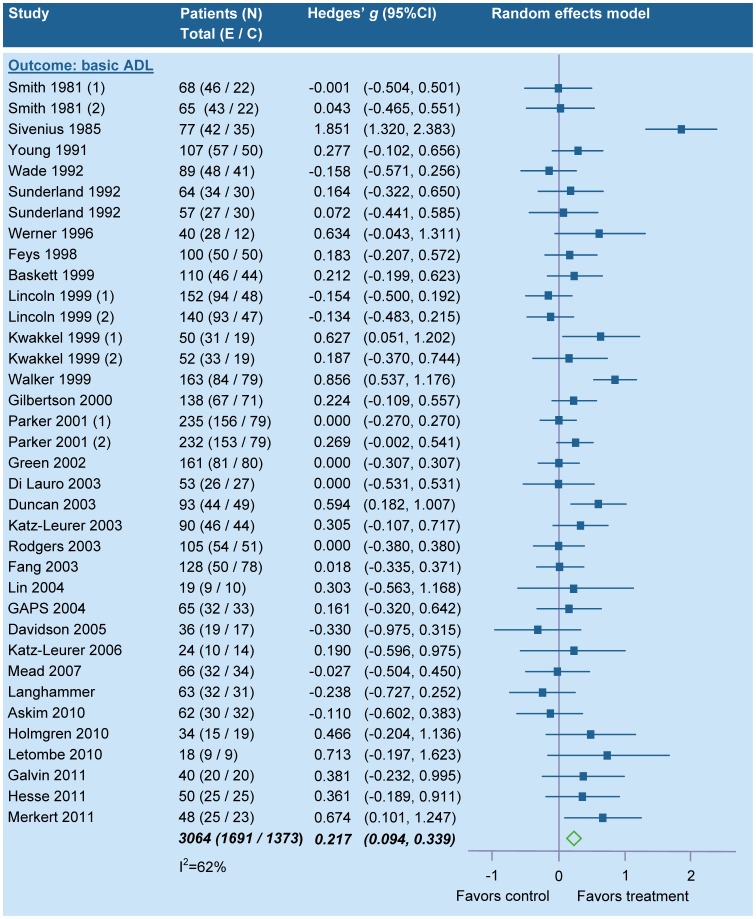
Summary effect sizes for physical therapy interventions – intensity of practice. Legend: ADL, Activities of daily living; C, Control group; CI, Confidence interval; E, Experimental group.

The analyses of high-intensity exercise therapy involved pooled data of the RCTs reporting on a treatment contrast between the experimental and control groups in terms of time spent in exercise therapy without the use of extensive equipment [19], [20]. The results of the meta-analyses for high-intensity exercise therapy are summarized in figure 7 (for details see table S2F in file S1).

#### High-intensity exercise therapy

In total, 80 RCTs were identified which used a treatment contrast in terms of time (N = 5776, PEDro score range 2 [465] to 8 [43], [44], [75], [127], [139], [171], [172], [174], [175], [443], [479], [499]–[509]) [43], [44], [51], [53]–[55], [60], [61], [74], [75], [80], [83], [84], [119], [127], [139], [144], [145], [147]–[149], [152], [156], [158], [171], [172], [174], [175], [178], [180], [189], [250], [307], [439], [440], [443], [458], [463]–[466], [468], [471]–[474], [477]–[482], [486], [494], [499]–[526], including patients in the hyper acute or acute rehabilitation phase, early rehabilitation phase, late rehabilitation phase, or chronic phase. In most of the RCTs, the interventions focused on the lower limb (n = 78). The mean treatment contrast amounted 17 hours over 10 weeks, indicating that on average the experimental groups received an additional therapy time of 17 hours when compared to the control groups.

Pooling the data resulted in significant homogeneous positive SESs for motor function of the paretic leg (synergy), motor function of the paretic arm (synergy), muscle strength of the leg, comfortable gait speed, maximum gait speed, muscle tone, and quality of life. Significant heterogeneous SESs were found for depression and anxiety, balance, and basic ADL. Meta-analyses for muscle strength of the arm, mental health of the patient, falls efficacy, walking ability, arm-hand activities, extended ADL, number of falls, and mental health of the caregiver resulted in nonsignificant SESs. The subgroup analysis for walking distance showed significantly different effects between phases, with a significant homogeneous positive SES for the chronic phase (n = 4), a nonsignificant SES for the early rehabilitation phase (n = 5), and a nonsignificant effect size for a group including patients regardless of timing poststroke (n = 1).

#### Neurological treatment approaches

Neurodevelopmental Treatment (NDT/Bobath) was delivered in 75 RCTs (N = 3502). For the purpose of the present review, the effects of NDT were analyzed in three different categories: (a) NDT vs. another intervention; (b) NDT vs. NDT plus another intervention; and (c) NDT vs. augmented NDT (for details see table S1G in file S1).

1. NDT vs. another intervention

NDT was compared with another type of intervention in 37 RCTs (N = 1670, PEDro score range 4 [108], [276], [527] to 8 [323], [366], [505]) [50], [82], [108], [118], [154], [264], [269], [270], [272], [273], [276], [278], [280]–[282], [301]–[303], [305], [313], [315], [316], [323], [326], [333], [366], [432], [457], [468], [505], [527]–[534].

Strong evidence for *equal effectiveness* compared to another intervention was found for muscle strength of the arm and depression. In addition, there was strong evidence for *unfavorable effects* of NDT on motor function (synergy), gait speed, spatiotemporal gait pattern functions, kinematics of the arm, arm-hand activities, self-reported arm-hand activities in daily life, basic ADL, and quality of life. There was moderate evidence that NDT is *equally effective* as another intervention regarding strength of the knee muscles, maximal weight bearing on the paretic leg, coordination, stability of the shoulder joint, shoulder pain, health beliefs, walking distance, and balance. Moderate evidence was found for an *unfavorable effect* of NDT on length of stay. Insufficient evidence was found for muscle strength of the leg, grip strength, muscle tone, brain activity, walking ability, and extended ADL.

2. NDT vs. NDT plus another intervention

NDT was compared with NDT plus another intervention in 33 RCTs (N = 1106, PEDro score range 2 [138] to 8 [88], [186], [191]) [49], [51], [59], [64], [66], [80], [88], [96], [123], [129], [138], [148], [151], [158], [186], [191], [199], [203], [217], [246], [331], [357], [390], [395], [399], [400], [413], [419], [433], [524], [535], [536].

There was strong evidence that NDT alone has *unfavorable effects* compared to NDT plus another intervention as regards motor function (synergy), muscle strength of the arm, walking speed, spatiotemporal gait pattern functions like stride length, muscle tone, range of motion, balance, walking ability, arm-hand activities, and basic ADL. Strong evidence was found that they are *equally effective* for gait kinematics. Moderate evidence was found for *unfavorable effect* of NDT when compared to NDT plus another intervention on muscle strength of the leg, walking distance, coordination, EMG contraction, shoulder subluxation, neglect, and aerobic capacity. Moderate evidence was found for *equal effectiveness* regarding symmetry while sitting, standing, performing sit-to-stand and reaching; depression; and ability to change posture from sit to stand and vice versa.

3. NDT vs. augmented NDT

The effect of more time spent in NDT versus less time spent in NDT was investigated in 6 RCTs (N = 786, PEDro score range 6 [513], [517] to 8 [503]–[505]) [503]–[505], [513], [517], [519].

There was strong evidence that NDT is *equally effective* as augmented NDT for the outcomes muscle strength, walking ability, arm-hand activities, basic ADL, and extended ADL. There was moderate evidence that augmented NDT is *beneficial* for motor function (synergy) and range of motion. In addition, moderate evidence was found for *equal effectiveness* regarding pain, depression, balance, sit-to-stand, handicap, and quality of life.

## Discussion

Interdisciplinary complex stroke rehabilitation is one of the fastest growing fields in stroke research [537]. With regard to physical therapy interventions, the present review shows that the number of RCTs has almost quadrupled in the past 10 years. Our meta-analyses suggest that there is strong evidence for 30 out of 53 interventions for beneficial effects on one or more outcomes. For a large proportion of the outcomes there is strong evidence that experimental interventions accomplish equal results when compared to ‘conventional therapy’, suggesting that the same results can be obtained with the control intervention, while no adverse events were reported. The generally small to medium SESs, indicating differential effects between 5 and 15%, mainly relate to those functions and activities specifically trained in the intervention, and are restricted to the period of intervention alone. While these findings were – globally – similar to the review from 2004, a comparison of the present results with the results of our previous review shows clear changes [12]. The main change lies in the increased number of interventions to which ‘strong evidence’ could be assigned and an increase in the number of outcomes for which the findings are statistically significant. In addition, shifts are observed for a few ‘strong evidence’ interventions with significant positive effects in 2004. For example, speed dependent treadmill training now shows neutral results for walking ability; rhythmic auditory cueing of gait currently shows neutral results for gait speed and stride length; and training of standing balance now also shows neutral results. In contrast to the 2004 review which reported no significant effects at the participation level, now mixed strength and cardiovascular exercises and leisure therapy show a favorable effect at the participation level. In general, exploring the possible moderator effect of poststroke timing largely did not show significant differences in effects. Higher intensity of practice proves to be an important aspect of effective physical therapy. This review also highlights that well controlled, dose-matched trials with significant effects in favor of the experimental intervention have been rather scarce (e.g. [76], [81], [110]). The above findings suggest that intensity of practice is a key factor in meaningful training after stroke, and that more practice is better [8]. This implies that our previous conclusion that high-intensity practice is better still holds [12], and that an additional therapy time of 17 hours over 10 weeks is necessary to find significant positive effects at both the body function level and activities and participation level of the ICF. In national clinical guidelines for stroke in the United Kingdom and the Netherlands, it is recommended that patients should be enabled to exercise at least 45 minutes on each weekday as long as there are rehabilitation goals and the patient tolerates this intensity [184], [538]. However, there is a big contrast between the recommended and actual applied therapy time. A survey in the Netherlands showed that patients with stroke admitted to a hospital stroke unit only received a mean of 22 minutes of physical therapy on weekdays [539]. Similarly, in the United Kingdom inpatients received 30.6 minutes physical therapy per day on which this therapy was given [540]. Contrary to previous reviews which concluded that neurological treatment approaches (NDT/Bobath) were not superior [12], [541], the present review demonstrates that neurological treatment approaches are less effective when compared to focused interventions such as mCIMT, bilateral arm training, or strengthening when applied in a task-specific way.

Repetition is an important principle in motor learning which reflects the Hebbian learning rule that connections between neurons are strengthened when they are simultaneously active (i.e., long term potentiation) [542]. An earlier review has shown that repetitive task training is a key modality of effective training in stroke [543]. This repetition aspect relates to “an active motor sequence performed repetitively within a single training session, with practice aiming towards a clear functional goal” [543]. However, this does not mean that each repetition should be identical to the previous ones. Instead, is suggested that implementing slight variation between repetitions is more successful [544]. Although we did not analyze ‘repetition’ separately, this modality is a feature included in many focused interventions for which strong evidence was found in the present review. For example, CIMT and gait training are both characterized by a high number of repetitions executed within a single treatment session, serving a functional goal.

**Figure 8:**
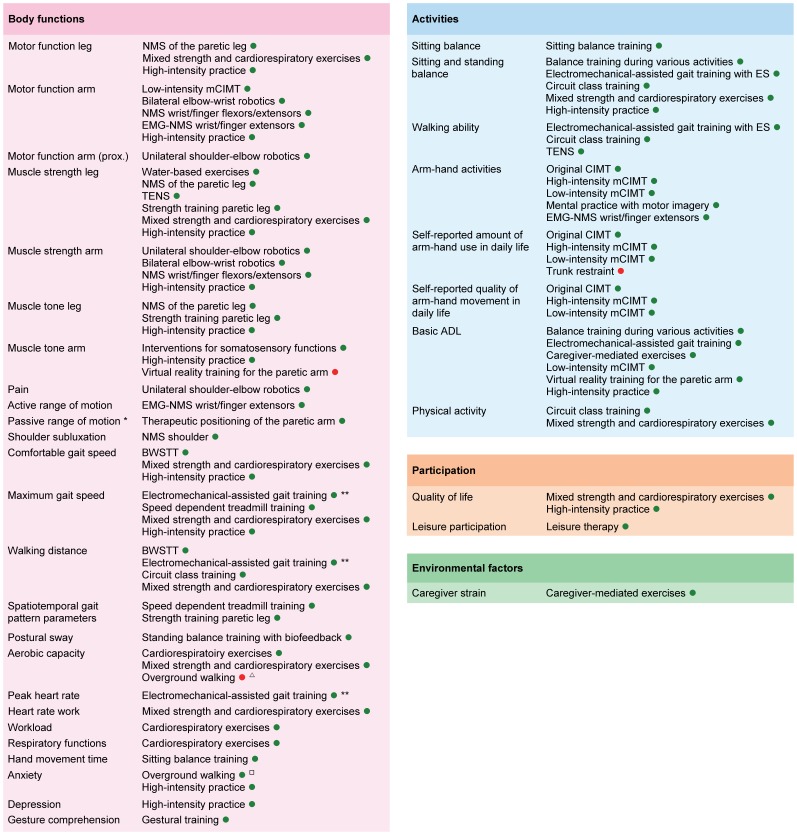
Overview of outcomes for which interventions are available with significant summarized effects. Legend: A green point indicates that the intervention has a significant positive effect on the outcome, while a red point indicates that the intervention has a significant negative effect on the outcome; *, shoulder external rotation; **, dependent walking patients in the early rehabilitation phase; ^▵^, dependent walking patients when compared to electromechanical-assisted gait training or BWSTT; ^□^, independent walking patients; BWSTT, Body-weight supported treadmill training; CIMT, Constraint-induced movement therapy; EMG-NMS, Electromyography-triggered neuromuscular stimulation; ES, Electrostimulation; mCIMT, modified Constraint-induced movement therapy; NMS, Neuromuscular stimulation; prox., Proximal; TENS, Transcutaneous electrical nerve stimulation.

To facilitate application of the findings presented in the current review in daily practice, it is necessary to further specify for which interventions there is strong evidence that patients benefit from this therapeutic intervention and for which outcome this evidence is valid. Therefore, figure 8 graphically displays the outcomes classified according to the ICF, with corresponding interventions for which is strong evidence that they significantly affect those outcomes. It should be noted that the clinical applicability of some interventions like electromechanical-assisted gait training and robot-assisted arm training is questionable, due to the accompanying high costs of the equipment. For these interventions, there are often alternative ‘strong evidence’ interventions available.

The large number of interventions and outcomes for which nonsignificant SESs were found in the meta-analyses (i.e. neutral results) suggests that for many forms of exercise therapy the same patient outcomes can be obtained with the control intervention. This implies that the physical therapist, in cooperation with the patient, has to decide for each individual patient which of these interventions is the optimal treatment option. In this clinical decision-making process, that preferably should be based on existing knowledge about the functional prognosis for outcome [22], [545], also resource use and possible alternative interventions should be taken into account.

It should also be noted that we found three significant negative SESs. The first being for overground walking (aerobic capacity; for dependent walking patients in the early rehabilitation phase when compared to electromechanical-assisted gait training or body-weight supported treadmill training), the second for virtual reality training for the paretic arm (muscle tone), and the third for trunk restraint (self-reported amount of arm-hand use in daily life). However, the meta-analysis for all these outcomes showed insufficient statistical power, suggesting that more trials are needed. Furthermore, although a negative SES was found for both overground walking and virtual reality training for the upper paretic limb, these interventions also show beneficial effects on one or more other outcomes. Therefore, we recommend that when physical therapists select one of these interventions, they should regularly monitor the outcomes which are at risk for being adversely affected by the intervention.

### (In)stability of Results in Trials

A comparison between the current results and those of our previous meta-analyses [12] shows that some interventions for which strong evidence was reported in 2004, such as rhythmic auditory cueing of gait, no longer have the same level of evidence, whereas other interventions with initially only indicative findings or no evidence, such as EMG-NMS for the paretic arm, now show significant positive small to moderate effect sizes. This finding reflects a lack of robustness of existing evidence favoring or disfavoring an intervention when new trials are added to the current pool of studies. In our opinion, this (in)stability of current evidence depends on several factors. First, differential effects seem to be largely dependent on the content and dose-matching of the therapy given in the control group [6], [546]. In a number of trials, the content and dosage of therapy applied in the control group is poorly defined. ‘Usual care’ frequently reflects the existing guidelines, suggesting that the patients in the control group received treatment according to the best available evidence at that moment. Obviously, researchers hypothesize that the added value of the experimental intervention will considerably exceed the existing standards of care, acknowledging that comparison of an experimental intervention with a real ‘sham’ or placebo intervention is not desirable in stroke rehabilitation, and is in most Western countries not allowed for medical ethical reasons. Second, many primary outcome measures do not appropriately reflect the underlying biological rationale for the content of the experimental therapy [547], whereas other outcomes may be rather insensitive to the changes introduced by physical therapy [548]. To improve comparability between trials applying the same intervention, international consensus about outcomes and timing of (follow-up) measurements is urgently needed [8], [549]. Third, of the 326 meta-analyses we performed, the statistical power was sufficient for only 58 meta-analyses divided over 28 interventions (e.g. training of sitting balance and (m)CIMT) and intensity of practice. The instability of SESs over time and hence the current level of evidence is mainly due to the low number of small-sized phase II trials [550]. The dominance of rather positive phase II trials in physical therapy may well reflect publication bias, since low-powered negative trials are less likely to be published [551]. In contrast, recent sufficiently powered phase III and IV trials in physical therapy, such as those on the impact of shoulder-elbow robotics [335] and body-weight supported treadmill training [91] yielded less positive findings than the previously published phase II trials on these type of interventions [552]. However, one may also argue that in small numbered monocenter trials, therapists are more committed to the trial than in multicenter trials. Fourth, heterogeneity of patient samples could have played a role [553]–[555]. Not only can differences between studies in inclusion criteria, resulting in between-study heterogeneity, play a role, but also within-study heterogeneity, especially in larger trials which tend to have less strict inclusion criteria. As referred to above, the therapeutic content of the experimental intervention applied was often poorly defined, since most journals do not allow publication of treatment protocols [556], preventing researchers from properly reporting on treatment content due to word limitations, replicating studies, or judging if interventions are sufficiently comparable to allow meta-analyses. Finally, the observed shifts in evidence may reflect the improved methodological quality of studies due to the introduction of the CONSORT Statement for reporting RCTs [557]. In the present review, the median PEDro score was found to have increased from 5 (IQR 4–6) for RCTs published before 2004 [12] to 6 (IQR 5–7) in the subsequent period. This finding suggests increased efforts by researchers to reduce bias in clinical trials [558], [559].

### Deficiencies in the Focus of Trials

Remarkably, only three RCTs started their intervention within the first days poststroke, despite evidence that most patients are physical inactive early poststroke [560] as well as the growing evidence of a greater potential for neuroplasticity in the first three to four weeks poststroke [561]. One may assume that giving appropriate training within this window of increased homeostatic neuroplasticity may enhance motor recovery. Although our subgroup analyses suggest that timing poststroke is only a significant moderator of effect sizes in a small number of interventions, this is based on very few trials that started in this critical phase of the first days or weeks poststroke.

While the strength of evidence is growing for certain physical therapy interventions, the cost-effectiveness of these interventions has so far hardly been subject of investigation [562], [563], and long-term outcomes have often not been systematically measured at fixed times post intervention. In addition, even though the main effects of intensity of practice are in favor of high-intensity training, there is still a paucity of well-controlled dose-response RTCs in the field of physical therapy directly investigating the impact of intensity of practice [19], [353].

### How to Proceed?

While acknowledging that interdisciplinary collaboration is a key aspect of stroke rehabilitation [3], we think it is important that each discipline should take responsibility to further extend the specific contribution of different types of therapy in the interdisciplinary care, in terms of evidence and implementation. Therefore, a roadmap is needed to prioritize research in the domain of physical therapy. In determining research priorities, different perspectives ought to be considered, like those of patients and their caregivers, clinicians, researchers and policy-makers [564], [565].

In our opinion, this roadmap should contain the following elements: (1) investigating dose-response relations in exercise therapy, in which the experimental and control groups receive the same type of intervention but with different dosage [566]; (2) investigating resource-efficient interventions to augment physical therapy and allow early supported discharge such as telerehabilitation [567] and caregiver-mediated exercises [174]; (3) investigating the benefits of an (very) early start of physical therapy poststroke [560] and continuation of poststroke therapy in the weekends; (4) investigating the cost-effectiveness of interventions and numbers needed to treat; (5) investigating the effectiveness of interventions which have so far only been investigated in phase II trials and from which patients may benefit; (6) investigating interventions that are used by physical therapists but have not been investigated in RCTs, like the effectiveness of falls prevention programs and physical fitness training in the context of secondary prevention. Finally, (7) investigating the mechanisms behind motor learning and stroke recovery, which are still poorly understood. Only translational research is able to bridge the gap between the effects of an intervention that have been found and the underlying mechanisms that may contribute to therapy-induced poststroke recovery. In order to understand what actually changes during stroke recovery, we need to discriminate between recovery of body functions (restitution) and learning to use compensation strategies in accomplishing tasks [568], [569]. In this respect, new therapeutic approaches in which physical exercise is combined with innovative treatments enhancing neuroplasticity in crucial (early) time windows, such as transcranial Direct Current Stimulation [570], [571], repetitive Transcranial Magnetic Stimulation [572], or neuropharmacological interventions [573], may be promising.

Stroke rehabilitation intervention research in the domain of physical therapy can be organized using a step-wise approach [6], [546]: interventions with positive effects in the first explorative stages on relevant consensus-based outcomes should become the subject of high-quality phase III and IV trials. In all cases, subgroups of patients should be selected which, from a biological perspective, would benefit the most from the intervention, while taking into account “the sensitive period for response to intervention” [574].

Implementation of research findings into daily practice is essential to improve quality of care, but is also challenging. First of all, because physical therapy as part of complex interdisciplinary stroke rehabilitation, contains several interrelated components that may be targeted at different levels (i.e., at service, operator, and/or treatment level) [8], [575], [576]. Second, physical therapy typically entails a cyclical process involving (1) assessment, to identify and quantify the patient’s needs; (2) goal setting, to define realistic and attainable goals for improvement; (3) intervention, to assist in the achievement of goals; and (4) reassessment, to assess progress against agreed goals [8]. For all of these four steps, a broad scientific base is available but the evidence is dynamic. Due to this complexity and it’s dynamics, a country wide postbachelor physical therapy course was started in 2008 in the Netherlands in which the different aspects of evidence-based practice in stroke are educated [541]. This one year course includes themes such as: (1) how to make clinical decisions; (2) how to measure outcome and clinical change; (3) how to estimate the individual prognosis for outcome at the activities level; and (4) how to select the best intervention. In addition, in this course special attention is paid to assumed pathophysiology and underlying working mechanisms of recovery poststroke. However, effective but efficient methods for physical therapists to keep their knowledge and skill level up-to-date in the long term needs further investigation.

### Limitations

Although this systematic review was performed with the greatest of care, there are some methodological limitations like the language restriction, not hand-searching conference proceedings, missing outcome data [577], not performing meta-analyses of individual patient data [578], and the lack of both a correction for multiple testing and systematic investigation of reporting bias. In addition, the observational nature of the subgroup analyses means they should be interpreted with caution, as it is known that subgroup analyses in meta-analyses can be less highly powered than analyses for main effects [29], [579], [580].

## Conclusion

In summary, the body of knowledge about physical therapy in stroke rehabilitation is still growing. This is evident both from the increased number of published RCTs with a low risk of bias, resulting in strong evidence for many physical therapy modalities, and from the exploration of innovative ways for efficient use of resources like circuit class training. This endorses the central role of physical therapy in interdisciplinary evidence-based stroke rehabilitation. Further confirmation of the evidence for physical therapy after stroke, and facilitating the transfer to clinical practice, requires a better understanding of (neurophysiological) mechanisms, including neuroplasticity, that drive stroke recovery, as well as the impact of physical therapy interventions on these underlying mechanisms. Thus, well-designed RCTs should address questions like: Which patients benefit most from a specific intervention? At what time poststroke should interventions be initiated? What are the underlying mechanisms that drive improvement of sensorimotor control? What are the preferred intervention characteristics, including the optimal dosage? And are interventions cost-effective? Subsequent meta-analyses should analyze the evidence using individual participant data. Finally, implementation strategies should be further explored in order to optimize the transfer of scientific knowledge into clinical practice.

The high growth in the number of RCTs on physical therapy stroke rehabilitation makes it virtually impossible for individual physical therapists to identify and ascertain the content of each relevant science citation indexed study. There is therefore a need for a worldwide continuing – online – update of the summarized evidence, discussed in the context of interdisciplinary stroke care.

## Supporting Information

File S1 **Contains Supporting Tables.** TABLE S1A. Title: Summary of physical therapy interventions – gait and mobility-related functions and activities. Legend: +, significant positive SES; = , nonsignficant SES; –, significant negative SES; ADL, Activities of daily living; C, Chronic phase; d, day(s); ER, Early rehabilitation phase; EMG, Electromyographic (H)AR, (hyper)acute rehabilitation phase; min, minutes; LR, Late rehabilitation phase; mos, months; RCTs, Randomized controlled trials; SES, Summary effect size; wk, week(s). TABLE S1B. Title: Summary of physical therapy interventions – arm-hand activities. Legend: +, significant positive SES; = , nonsignficant SES; –, significant negative SES; ?, unclear; ADL, Activities of daily living; C, Chronic phase; d, day(s); CIMT, Constraint-induced movement therapy; ER, Early rehabilitation phase; (H)AR, (hyper)acute rehabilitation phase; LR, Late rehabilitation phase; min, minutes; mCIMT, modified Constraint-induced movement therapy; mos, months; RCTs, Randomized controlled trials; wk, week(s); SES, Summary effect size. TABLE S1C. Title: Summary of physical therapy interventions – physical fitness. Legend: +, significant positive SES; = , nonsignficant SES; ?, unclear; ADL, Activities of daily living; C, Chronic phase; d, day(s); ER, Early rehabilitation phase; (H)AR, (hyper)acute rehabilitation phase; LR, Late rehabilitation phase; RCTs, Randomized controlled trials; min, minutes; mos, months; SES, Summary effect size; wk, week(s). TABLE S1D. Title: Summary of physical therapy interventions – activities of daily living. Legend: +, significant positive SES; = , nonsignficant SES; C, Chronic phase; d, day(s); ER, Early rehabilitation phase; (H)AR, (hyper)acute rehabilitation phase; LR, Late rehabilitation phase; min, minutes; mos, months; RCTs, Randomized controlled trials; SES, Summary effect size; wk, week(s). TABLE S1E. Title: Summary of physical therapy interventions – other. Legend: = , nonsignficant SES; C, Chronic phase; d, day(s); ER, Early rehabilitation phase; (H)AR, (hyper)acute rehabilitation phase; LR, Late rehabilitation phase; min, minutes; mos, months; RCTs, Randomized controlled trials; SES, Summary effect size; wk, week(s). TABLE S1F. Title: Summary of physical therapy interventions – intensity of practice. Legend: ?, unclear; +, significant positive SES; = , nonsignficant SES; ADL, Activities of daily living; C, Chronic phase; ER, Early rehabilitation phase; (H)AR, (hyper)acute rehabilitation phase; h, hours; LR, Late rehabilitation phase; RCTs, Randomized controlled trials; SES, Summary effect size; wk, weeks. TABLE S1G. Title: Summary of physical therapy interventions – neurological treatment approaches. Legend: +, significant positive effect; = , nonsignficant effect; –, significant negative effect; ?, unclear; ADL, Activities of daily living; BWSTT, Body-weight supported treadmill training; C, Chronic phase; EMG-BF, Electromyographic biofeedback; EMG, Electromyograpic; EMG-NMS, Electromyography-triggered neuromuscular stimulation; ER, Early rehabilitation phase; (H)AR, (hyper)acute rehabilitation phase; LR, Late rehabilitation phase; mCIMT, modified Constraint-induced movement therapy; NDT, Neurodevelopmental treatment; NMS, Neuromuscular stimulation; RCTs, Randomized controlled trials; SES, Summary effect size; TENS, Transcutaneous electrical nerve stimulation. TABLE S2A. Title: Summary of the evidence for physical therapy interventions – gait and mobility-related functions and activities. Legend: 10MWT, 10-meter walk test; 12MWT, 12-minute walk test; 3MWT, 3-minute walk test; 5MWT, 5-meter walk test; 6MWT, 6-minute walk test; 8MWT, 8-meter walk test; AAP, Adelaide activities profile; ABC, Activities-specific balance confidence scale; ADL, Activities of daily living; BBA, Brunel balance assessment; BBS, Berg balance scale; BI, Barthel index; BP, Blood pressure; BWD, Body-weight distribution; C, Chronic phase; CI, Confidence interval; CSS, Composite spasticity scale; CNS, Canadian neurological scale; DB, Dynamic balance; DST, Double support time; EFAP, Emory functional ambulation profile; EMS, Elderly mobility scale; ER, Early rehabilitation phase; FAC, Functional ambulation categories; FAI, Frenchay activities index; FES-I, Falls-efficacy scale; FIM, Functional independence measure; FMA, Fugl-meyer assessment; FR, Functional reach; FSST, Four square step test; GDS-15, Geriatric depression scale - 15;GRF, Ground reaction force; HADS, Hospital anxiety and depression scale; HR, Heart rate; LLFDI, Late life function and disability instrument; LR, Late rehabilitation phase; LRT, Lateral reach test; MAS, Modified ashworth scale; MAS*, Motor assessment scale; mEFAP, modified Emory functional ambulation profile; MI, Motricity index; MMAS*, modified Motor assessment scale; MRC, Medical research council; mRS, modified Rankin scale; NA, Not applicable; NEADL, Nottingham extended ADL index; NHP, Nottingham health profile; NIHSS, National institutes of health stroke scale; NS, Not significant; PADS, Physical activity and disability scale; PASIPD, Physical activity scale for individuals with physical disabilities; PASS, Postural assessment scale for stroke; QoL, Quality of life; RMI, Rivermead mobility index; RMA, Rivermead motor assessment; RMA GF, RMA gross function; RMA LT, RMA leg and trunk; ROM, Range of motion; RPE, Rating of perceived exertion; S, Significant; SA-SIP30, Stroke-adapted 30-item version of the sickness impact profile; SAS, Stroke activities scale; SES, Summary effect size; SF-36, 36-Item short form health survey; SB, Static balance; SI, Spasticity index; SIS, Stroke impact scale; SPPB, Short physical performance battery; SST, Single-support time; ST, Step test; STREAM, Stroke rehabilitation assessment of movement instrument; STS, Sit-to-stand; TCT, Trunk control test; TIS, Trunk impairment scale; TMS, Toulouse motor scale; TUG, Timed up and go test; WAQ, Walking ability questionnaire; WD, Walking distance; WQ, Walking quality; WS, Walking speed gait analysis. TABLE S2B. Title: Summary of the evidence for physical therapy interventions – arm-hand activities. Legend: 10CMT, 10-cup moving test; ADL, Activities of daily living; AFT, Arm function test; AMAT, Arm motor ability test; ARAT, Action research arm test; BBT, Box and block test; BI, Barthel index; C, Chronic phase; CAHAI, Chedoke arm and hand activity inventory; CI, Confidence interval; CMMSA, Chedoke-McMaster stroke assessment; ER, Early rehabilitation phase;FE, Functional evaluation; FIM, Functional independence measure; FMC, Fine motor control; FMA, Fugl-meyer assessment; FTHUE, Functional test for the hemiplegic upper extremity; GP, Grip power; GS, Grip strength; JTHFT, Jebsen-Taylor hand function test; LR, Late rehabilitation phase; MAL, Motor activity log; MAS, Modified ashworth scale; MAS*, Motor assessment scale; mFMA, modified Fugl-meyer assessment; MP, Motor power; MRC, Medical research council; MSS, Motor status scale; mBI, modified Barthel index; NA, Not applicable; NS, Not significant; NSA, Nottingham sensory assessment; PPT, Perdue pegboard test; PS, Pinch strength; ROM, Range of motion; S, Significant; SES, Summary effect size; SIS, Stroke impact scale; TEMPA, Test d’evaluation des membres supérieurs de personnes agéés; UEFT, Upper extremity function test; VAS, Visual analogue scale; WFMT, Wolf motor function test. TABLE S2C. Title: Summary of the evidence for physical therapy interventions – physical fitness. Legend: 10MWT, 10-meter walk test; 12MWT, 12-minute walk test; 5MWT, 5-meter walk test; 6MWT, 6-minute walk test; ADL, Activities of daily living; ARAT, Action research arm test; BBS, Berg balance scale; BI, Barthel index; BMI, Body mass index; BP, Blood pressure; C, Chronic phase; CI, Confidence interval; CMMSA, Chedoke-McMaster stroke assessment; EQ, EuroQoL 5D; ER, Early rehabilitation phase; FAP, Functional ambulation profile; FEV1, Forced expiratory volume in 1 second; FIM, Functional independence measure; FMA, Fugl-meyer assessment; FR, Functional reach; FSST, Four square step test; FTHUE, Functional test for the hemiplegic upper extremity; GF, Grip force; GS, Grip strength; HADS, Hospital anxiety and depression scale; HR, Heart rate; IADL, Instrumental ADL; JTHFT, Jebsen-Taylor hand function test; LLFDI, Late life function and disability instrument; LR, Late rehabilitation phase; MAS, Modified ashworth scale; NA, Not applicable; NEADL, Nottingham extended ADL index; NHPT, Nine hole peg test; NS, Not significant; O2cost, Oxygen cost; PADS, physical activities and disability scale; PASIPD, Physical activity scale for individuals with physical disabilities; PF, Pinch force; PPT, Perdue pegboard test; RER, Respiratory exchange ratio; RMA GF, Rivermead motor assessment gross function; RMI, Rivermead mobility index; S, Significant; SES, Summary effect size; SF-36, 36-item Short form health survey; SLC90, Symptom checklist-90-R; STS, Sit-to-stand; TMS, Tolouse motor scale; TUG, Timed up and go test; VE, Ventilatory exchange; VO2max, Ventilatory oxygen uptake, WD, Walking distance; WS, WQ, Walking questionnaire; Walking speed gait analysis. TABLE S2D. Title: Summary of the evidence for physical therapy interventions – activities of daily living. Legend: *1 RCT with 2 comparisons; NLQ, Nottingham leisure questionnaire; BDI, Beck depression inventory; C, Chronic phase; CES-D, Centre for epidemiologic studies for depression scale; CI, Confidence interval; ER, Early rehabilitation phase; GHQ, General health questionnaire; GWBS, General well-being scale; LR, Late rehabilitation phase; NA, Not applicable; NS, Not significant; TLAS, Total leisure activities score; TLS, Total leisure score; S, Significant; SES, Summary effect size; SIP, Stroke impact profile; SA-SIP30, Stroke-adapted 30-item version of the sickness impact profile; WDI, Wakefield depression inventory. TABLE S2E. Title: Summary of the evidence for physical therapy interventions – other. Legend: CI, Confidence interval; C, Chronic phase; LR, Late rehabilitation phase; MIP, Maximal inspiratory pressure; S, Significant; SES, Summary effect size. TABLE S2F. Title: Summary of the evidence for physical therapy interventions – intensity of practice. Legend: 10MWT, 10-meter walk test; 5MWT, 5-meter walk test; ABC, Activities-specific balance confidence scale; ADL, Activities of daily living; ARAT, Action research arm test; BBS, Berg balance scale; BDI, Beck depression inventory; BI, Barthel index; C, Chronic phase; CI, Confidence interval; COOP scale, Dartmouth primary care cooperative information functional health assessment; ER, Early rehabilitation phase; FAC, Functional ambulation categories; FAI, Frenchay activities index; FES-I, Falls-efficacy scale; FIM, Functional independence measure; FMA, Fugl-meyer assessment; FR, Functional reach; FSST, Four square step test; FTHUE, Functional test for the hemiplegic upper extremity; GDS, Geriatric depression scale –15; GHQ, General health questionnaire; GS, Grip strength; HADS, Hospital anxiety and depression scale; IADL, Instrumental ADL; LHS, London handicap scale; LR, Late rehabilitation phase; MAS, Modified ashworth scale; mBI, modified Barthel index; MI, Motricity index; mRMI, modified Rivermead mobility index; NA, Not applicable; NEADL, Nottingham extended ADL index; NHP, Nottingham health profile; NHPT, Nine hole peg test; NS, Not significant; PASS, Postural assessment scale for stroke; POR, profile of recovery; PS, Pinch strength; RMA, Rivermead motor assessment; RMI, Rivermead mobility index; S, Significant; SCL-90, Symptom checklist-90-R; SES, Summary effect size; SF-36, 36-item Short form health survey; SIP, Stroke impact scale, SIS, Stroke impact scale; ST, Step test; STREAM, Stroke rehabilitation assessment of movement instrument; WD, Walking distance; WS, Walking speed gait analysis.
(PDF)

Checklist S1 **PRISMA Checklist.**
(DOC)
