# Supplementary material for: What Is the Evidence for Physical Therapy Poststroke? A Systematic Review and Meta-Analysis
Source: PLoS One. 2014 Feb 4;9(2):e87987. doi: 10.1371/journal.pone.0087987 (PMC3913786; doi:10.1371/journal.pone.0087987)
Supplement: File S1 — Contains Supporting Tables. TABLE S1A. Title: Summary of physical therapy interventions – gait and mobility-related functions and activities. Legend: +, significant positive SES; = , nonsignficant SES; –, significant negative SES; ADL, Activities of daily living; C, Chronic phase; d, day(s); ER, Early rehabilitation phase; EMG, Electromyographic (H)AR, (hyper)acute rehabilitation phase; min, minutes; LR, Late rehabilitation phase; mos, months; RCTs, Randomized controlled trials; SES, Summary effect size; wk, week(s). TABLE S1B. Title: Summary of physical therapy interventions – arm-hand activities. Legend: +, significant positive SES; = , nonsignficant SES; –, significant negative SES; ?, unclear; ADL, Activities of daily living; C, Chronic phase; d, day(s); CIMT, Constraint-induced movement therapy; ER, Early rehabilitation phase; (H)AR, (hyper)acute rehabilitation phase; LR, Late rehabilitation phase; min, minutes; mCIMT, modified Constraint-induced movement therapy; mos, months; RCTs, Randomized controlled trials; wk, week(s); SES, Summary effect size. TABLE S1C. Title: Summary of physical therapy interventions – physical fitness. Legend: +, significant positive SES; = , nonsignficant SES; ?, unclear; ADL, Activities of daily living; C, Chronic phase; d, day(s); ER, Early rehabilitation phase; (H)AR, (hyper)acute rehabilitation phase; LR, Late rehabilitation phase; RCTs, Randomized controlled trials; min, minutes; mos, months; SES, Summary effect size; wk, week(s). TABLE S1D. Title: Summary of physical therapy interventions – activities of daily living. Legend: +, significant positive SES; = , nonsignficant SES; C, Chronic phase; d, day(s); ER, Early rehabilitation phase; (H)AR, (hyper)acute rehabilitation phase; LR, Late rehabilitation phase; min, minutes; mos, months; RCTs, Randomized controlled trials; SES, Summary effect size; wk, week(s). TABLE S1E. Title: Summary of physical therapy interventions – other. Legend: = , nonsignficant SES; C, Chronic phase; d, [file pone.0087987.s001.pdf]

TABLE S1A. Summary of physical therapy interventions – gait and mobility-related functions and activities

Intervention	n RCTs	N Patients	Start study time poststroke, n RCTs ([H]AR/ER/LR/C/?)	Intensity of intervention (ranges between studies given only)	Main results meta-analyses	Risk of bias (PEDro) of RCTs (ranges between studies given only)	References
Early mobilization	2	103	2/0/0/0/1	2-4 times/d, ≤2 wk	Strong evidence (=): complications, neurological deterioration early poststroke, fatigue, basic ADL, discharge home	8	[1,2]
Sitting balance training	6	150	0/5/0/1/0	30-60 min/d, 5 d/wk, 2-4 wk	Strong evidence (+): sitting balance, hand movement time<br>Strong evidence (=): ground reaction force, symmetry while sitting or standing, walking ability, basic ADL	4 – 8	[3-8]
Sit-to-stand training	5	163	0/4/0/1/0	15-45 min/d, 3-5 d/wk, 1-6 wk	Strong evidence (=): body weight distribution, sit-to-stand	4 – 6	[9-13]
Standing balance training without biofeedback	4	199	0/3/0/1/0	45-60 min/d, 2-5 d/wk, 2-8 wk	Strong evidence (=): postural sway, balance, sit-to-stand, walking ability	4 – 8	[14-17]
Standing balance training with biofeedback – force and position feedback	12	333	0/6/5/1/0	15-60 min, 2-5 d/wk, 2-4 wk	Strong evidence (+): postural sway<br>Strong evidence (=): motor function leg (synergy), comfortable gait speed, cadence, step length, mono and bipedal phase, balance, walking ability, basic ADL	3 – 6	[13,18-29]
Balance training during various activities	11	419	0/5/3/3/0	30-60 min/d, 3-7 d/wk, 3-10 wk	Strong evidence (+): balance, basic ADL<br>Strong evidence (=): comfortable gait speed, falls-efficacy, walking ability, quality of life	4 – 8	[30-40]
Body-weight supported treadmill training	18	1158	0/11/0/8/0	15-90 min/d, 3-6 d/wk, 2-6 wk	Strong evidence (+): comfortable gait speed, walking distance<br>Strong evidence (=): motor function leg (synergy), maximum gait speed, cadence, stride length, aerobic capacity, energy expenditure, balance, walking ability, quality of life	4 – 8	[41-61]
Electromechanical-assisted gait training	16	785	0/11/1/4/0	15-60 min/d, 3-7 d/wk, 2-10 wk		4 – 8	[48,55,62-77]
Electromechanical-assisted gait training without functional electrostimulation	16	785	0/11/1/4/0	15-60 min/d, 3-7 d/wk, 2-10 wk	Strong evidence (+): maximum gait speed, walking distance, peak heart rate, basic ADL<br>Strong evidence (=): neurological functions, motor function paretic leg (synergy), muscle strength, comfortable gait speed, cadence, step length, heart rate rest, balance, walking ability, quality of life	4 – 8	[48,55,62-67,69-77]
Electromechanical-assisted gait training with functional electrostimulation	3	144	0/2/0/1/0	20 min/d, 5-7 d/wk, 3-4 wk	Strong evidence (+): balance, walking ability<br>Strong evidence (=): maximum gait speed, basic ADL	6	[62,63,71]
Speed dependent treadmill training (without body-weight support)	13	610	0/3/1/9/0	8-60 min/d, 3-5 d/wk, 2 wk – 6 mos	Strong evidence (+): maximum gait speed, step width<br>Strong evidence (=): comfortable gait speed, gait speed endurance, walking distance, cadence, stride length, aerobic capacity, balance, walking ability	4 – 8	[41,78-90]
Overground walking	19	1008	0/5/4/10/0	15-60 mind, 1-5 d/wk, 2 wk – 6 mos	Strong evidence (+): anxiety (independent walking patients)<br>Strong evidence (=): comfortable gait speed, maximum gait speed, walking distance, cadence, stride length, stride time, symmetry gait pattern, number of falls, peak heart rate, diastolic blood pressure, systolic blood pressure, depression, balance, walking ability, basic ADL, extended ADL<br>Strong evidence (–): aerobic capacity (dependent walking patients)	2 – 8	[44,45,56,57,62,72,73,76,77,90-103]
Rhythmic gait cueing	6	231	0/4/0/2/0	30 min/d, 5 d/wk, 3-12 wk	Strong evidence (=): gait speed, cadence, stride length, symmetry gait pattern	3 – 7	[104-109]
Community walking	3	94	0/1/0/2/0	30-60 min/d, 2-5 d/wk, 2.5-7 wk	Strong evidence (=): maximum gait speed, walking distance, balance confidence	6 – 8	[87,110,111]
Virtual reality mobility training	6	150	0/6/0/0/0	20-60 min/d, 3-5 d/wk, 2-4 wk	Strong evidence (=): comfortable gait speed, maximum gait speed, step length, walking ability	5 – 7	[112-118]
Circuit class training (gait and mobility-related functions and activities)	8	359	0/1/3/4/0	30-75 min/d, 3-5 d/wk, 4-19 wk	Strong evidence (+): walking distance, balance, walking ability, physical activity<br>Strong evidence (=): muscle strength, gait speed, number of falls, falls-efficacy, depression, basic ADL, extended ADL, quality of life	5 – 8	[34,38,93,98,99,119-122]
Caregiver-mediated exercises (gait and mobility-related functions and activities)	3	350	0/2/0/1/0	35-60 min/d, 2-7 d/wk, 8 wk – 6 mos	Strong evidence (+): basic ADL, caregiver strain<br>Strong evidence (=): extended ADL	4 – 8	[91,123,124]
Orthosis for walking	4	137	0/1/0/1/2	24 wk – 6 mos	Strong evidence (=): comfortable gait speed	2 – 7	[43,125-127]
Water-based exercises	3	65	0/0/0/3/0	45-60 min/d, 2-3 d/wk, 8-12 wk	Strong evidence (+): muscle strength<br>Strong evidence (=): balance	5 – 6	[128-130]
Interventions for somatosensory functions of the paretic leg	7	211	0/3/2/1/0	20-48 min/d, 3-5 d/wk, 2-8 wk	Strong evidence (=): motor function leg (synergy), gait speed, balance	5 – 8	[14,131-135]
Electrostimulation of the paretic leg	26	814	0/13/4/7/2	9-60 min/d, 2-7 d/wk, 3-8 wk		2 – 8	[63,71,126,132,136-158]
– NMS	18				Strong evidence (+): motor function leg (synergy), muscle tone, muscle strength		[63,71,126,136,137,139-141,144-147,149-152,157,158]

					Strong evidence (=): active range of motion, gait speed, cadence, step length, symmetry gait, balance, walking ability, basic ADL		
– EMG-NMS	2				Strong evidence (=): muscle tone, basic ADL		[142,153]
– TENS	5				Strong evidence (+): muscle strength, walking ability<br>Strong evidence (=): muscle tone, active range of motion ankle, gait speed, walking distance		[132,143,148,154-156]
Electromyographic biofeedback for the paretic leg	11	254	0/3/0/7/1	20-60 min/d, 2-5 d/wk, 2-12 wk	Strong evidence (=): range of motion, step length, gait speed, EMG activity	2 – 7	[104,137,159-167]

+, significant positive SES; =, nonsignficant SES; –, significant negative SES; ADL, Activities of daily living; C, Chronic phase; d, day(s); ER, Early rehabilitation phase; EMG, Electromyographic (H)AR, (hyper)acute rehabilitation phase; min, minutes; LR, Late rehabilitation phase; mos, months; RCTs, Randomized controlled trials; SES, Summary effect size; wk, week(s)

TABLE S1B. Summary of physical therapy interventions – arm-hand activities

Intervention	n RCTs	N Patients	Start study time poststroke, n RCTs ([H]AR/ER/LR/C/?)	Intensity of intervention (ranges between studies given only)	Main results meta-analyses	Risk of bias (PEDro) of RCTs (ranges between studies given only)	References
Therapeutic positioning of the paretic arm	5	140	0/1/0/0/0	2-4 times/d, 20-30 min/session, 5-7 d/wk	Strong evidence (+): passive range of motion shoulder external rotation<br>Strong evidence (=):passive range of motion shoulder internal rotation, contracture shoulder, pain (rest and movement), basic ADL	6 – 7	[168-172]
Reflex-inhibiting positions and immobilization techniques for the paretic wrist and hand	8	197	0/2/1/5/0	2-22h/d, 5-7 d/wk, 1-6 mos	Strong evidence (=): muscle tone, pain, passive range of motion	3 – 8	[173-180]
Air-splints around the paretic arm	5	285	0/3/1/1/0	30-45 min/d, 5-7 d/wk, 3-6 wk	Strong evidence (=): motor function arm (synergy), muscle tone, somatosensory function, pain, arm-hand activities	4 – 8	[181-186]
Supportive techniques or devices for the prevention or treatment of glenohumeral subluxation and/or hemiplegic shoulder pain	3	142	0/3/0/0/0	4-6 wk	Strong evidence (=): motor function arm (synergy), pain	4 – 7	[187-189]
Bilateral arm training	22	823	0/3/1/18/0	20 min – 6 h/d, 2-6 d/wk, 1-8 wk	Strong evidence (=): motor function arm, muscle strength, arm-hand activities, self-reported arm-hand use in daily life, basic ADL	2 – 8	[190-211]
(modified) Constraint-induced movement therapy	41	1342	0/11/3/27/0			2 – 8	[190,196,205,207,209,211-249]
– Original CIMT	1	222	0/0/1/0/0	6 h/d, 5 d/wk, 2 wk	Strong evidence (+): arm-hand activities, self-reported amount of arm-hand use in daily life, self-reported quality of arm-hand movement in daily life		[225,226]
– High-intensity mCIMT	15			3- 6 h/d, 1-7 d/wk, 2-8 wk	Strong evidence (+): arm-hand activities, self-reported amount of arm-hand use in daily life, self-reported quality of arm-hand movement in daily life<br>Strong evidence (=): motor function arm (synergy), basic ADL		[196,207,212,216,217,223,224,227,232-235,238,240-242,245,249]
– Low-intensity mCIMT	22			30 min – 2 h/d, 3-7 d/wk, 2-12 wk	Strong evidence (+): motor function arm (synergy), arm-hand activities, self-reported amount of arm-hand use in daily life, self-reported quality of arm-hand movement in daily life, basic ADL<br>Strong evidence (=): arm-hand related quality of life		[190,205,208,209,211,213-215,218,219,221,222,228-231,237,239-242,244,246,248]
– Mitt use	2			2 wk	[pooling not possible]		[220,243]
Robot-assisted arm training	22	648	0/8/1/13/0	20-120 min/d, 3-5 d/wk, 3-12 wk		4 – 8	[199,250-271]
– Unilateral shoulder-elbow robotics	15				Strong evidence (+): motor function arm proximal (synergy), muscle strength, muscle strength arm proximal, pain<br>Strong evidence (=): motor function arm (synergy), motor function arm distal, muscle strength arm distal, muscle tone, arm-hand activities, basic ADL, quality of life		[199,250,252-255,257,258,260,262,263,267,269-271]
– Bilateral elbow-wrist robotics	2				Strong evidence (+): motor function arm (synergy), muscle strength		[256,268]
– Shoulder-elbow-wrist-hand robotics	2				Strong evidence (=): motor function arm (synergy), muscle strength distal		[259,264]
– Wrist robotics	2				[pooling not possible]		[265,266]
– Wrist-hand robotics	1				[pooling not possible]		[261]
Mental practice with motor imagery	14	424	0/6/0/8/0	10-60 min/d, 2-5 d/wk, 3-10 wk	Strong evidence (+): arm-hand activities<br>Strong evidence (=): motor function arm (synergy), muscle strength, basic ADL	4 – 7	[272-284]
Mirror therapy for the paretic arm	7	255	0/1/2/4/0	30-60 min/d, 2-6 d/wk, 4-6 wk	Strong evidence (=): motor function arm (synergy), muscle tone, pain, arm-hand activities	5 – 8	[278,285-290]
Virtual reality training for the paretic arm	15	357	0/4/2/9/0	20-60 min/d, 3-7 d/wk, 10 d – 7 wk	Strong evidence (+): basic ADL<br>Strong evidence (=): motor function arm (synergy), arm-hand activities<br>Strong evidence (–): muscle tone	3 – 8	[291-306]
Electrostimulation of the paretic arm	49	1521	0/23/6/19/1	20 min – 8 h/d, 2-7 d/wk, 2 wk – 5 mnd		3 – 8	[143,193,197,255,259,307-354]
– NMS wrist/finger extensors					Strong evidence (=): motor function arm (synergy), active range of motion, muscle strength, arm-hand activities		
– NMS wrist/finger flexors/extensors					Strong evidence (+): motor function arm (synergy), muscle strength<br>Strong evidence (=): arm-hand activities		
– NMS shoulder					Strong evidence (+): subluxation<br>Strong evidence (=): motor function arm (synergy), range of motion, pain		
– EMG-NMS wrist/finger extensors					Strong evidence (+): motor function arm (synergy), active range of motion, arm-hand activities<br>Strong evidence (=): muscle strength, muscle tone		
– EMG-NMS wrist/finger flexors/extensors					Strong evidence (=): motor function arm (synergy), arm-hand activities		
– TENS					Strong evidence (=): muscle tone, basic ADL		
Electromyographic biofeedback of the	11	317	0/3/4/3/1	20-60 min/d, 2-5 d/wk, 2-6 wk	Strong evidence (=): motor function arm (synergy), active range of motion,	2 – 7	[160,355-364]

paretic arm					arm-hand activities		
Trunk restraint	4	86	0/0/0/4/0	40 min – 6 h/d, 2-5 d/wk, 2-5 wk	Strong evidence (=): active range of motion, arm-hand activities<br>Strong evidence (–): self-reported amount of arm-hand use in daily life	4 – 8	[245,365-367]
Interventions for somatosensory functions of the paretic arm	12	580	0/3/4/5/0	30-180 min, 2-6 d/wk, 2 wk – 3 mos	Strong evidence (+):muscle tone, somatosensory functions<br>Strong evidence (=): motor function arm (synergy), muscle strength, pain, arm-hand activities, basic ADL	3 – 9	[134,182,183,185,315,316,328,368-374]

+, significant positive SES; =, nonsignficant SES; –, significant negative SES; ?, unclear; ADL, Activities of daily living; C, Chronic phase; d, day(s); CIMT, Constraint-induced movement therapy; ER, Early rehabilitation phase; (H)AR, (hyper)acute rehabilitation phase; LR, Late rehabilitation phase; min, minutes; mCIMT, modified Constraint-induced movement therapy; mos, months; RCTs, Randomized controlled trials; wk, week(s); SES, Summary effect size

TABLE S1C. Summary of physical therapy interventions – physical fitness

Intervention	n RCTs	N Patients	Start study time poststroke, n RCTs ([H]AR/ER/LR/C)	Intensity of intervention (ranges between studies given only)	Main results meta-analyses	Risk of bias (PEDro) of RCTs (ranges between studies given only)	References
Strength exercises for the paretic leg	19	786	0/6/2/11	30-60 min/d, 2-5 d/wk, 4-16 wk	Strong evidence (+): muscle strength leg, muscle tone, spatiotemporal gait pattern parameters<br>Strong evidence (=): motor function leg (synergy), comfortable gait speed, maximum gait speed, walking distance, aerobic capacity, heart rate work, workload, physiological cost index, walking ability, basic ADL, quality of life	2 – 8	[120,375-393]
Strength exercises for the paretic arm	9	327	0/3/0/6	5-60 min/d, 2-5 d/wk, 2-6 wk	Strong evidence (=): motor function arm (synergy), muscle strength arm, pain, range of motion, arm-hand activities	2 – 7	[52,378,379,391,394-398]
Cardiorespiratory exercises	13	531	0/4/0/9	10-60 min/d, 2-5 d/wk, 4 wk – 6 mos	Strong evidence (+): aerobic capacity, workload, respiratory functions<br>Strong evidence (=): motor function leg (synergy), muscle strength leg, comfortable gait speed, maximum gait speed, walking distance, heart rate rest, heart rate work, blood pressure rest, physiological cost index, body composition, blood variables, balance, walking ability	4 – 8	[49,58,82-86,88,128,387,388,399-406]
Mixed strength and cardiorespiratory exercises	13	608	0/5/2/6	45-100 min/d, 3-4 d/wk, 4-19 wk	Strong evidence (+): motor function leg (synergy), muscle strength leg, comfortable gait speed, maximum gait speed, walking distance, aerobic capacity, heart rate work, balance, physical activity, quality of life<br>Strong evidence (=): motor function arm (synergy), muscle strength arm, physiological cost index, depression, walking ability, arm-hand activities, basic ADL, extended ADL	3 – 8	[93,96,98,99,121,387,388,407-416]

+, significant positive SES; =, nonsignficant SES; ?, unclear; ADL, Activities of daily living; C, Chronic phase; d, day(s); ER, Early rehabilitation phase; (H)AR, (hyper)acute rehabilitation phase; LR, Late rehabilitation phase; RCTs, Randomized controlled trials; min, minutes; mos, months; SES, Summary effect size; wk, week(s)

TABLE S1D. Summary of physical therapy interventions – activities of daily living

Intervention	n RCTs	N Patients	Start study time poststroke, n RCTs ([H]AR/ER/LR/C)	Intensity of intervention (ranges between studies given only)	Main results meta-analyses	Risk of bias (PEDro) of RCTs (ranges between studies given only)	References
Interventions for apraxia – gestural training	2	46	0/0/0/2	50 min/d, 3 d/wk, 30-35 sessions	Strong evidence (+): gesture comprehension<br>Strong evidence (=): ideational apraxia, ideomotor apraxia	4 – 5	[417,418]
Leisure therapy	6	928	0/3/1/2	30-60 min/d, <1 – 1 d/wk, 5 wk – 6 mos	Strong evidence (+):leisure participation<br>Strong evidence (=): depression, mood, quality of life	4 – 7	[419-424]

+, significant positive SES; =, nonsignficant SES; C, Chronic phase; d, day(s); ER, Early rehabilitation phase; (H)AR, (hyper)acute rehabilitation phase; LR, Late rehabilitation phase; min, minutes; mos, months; RCTs, Randomized controlled trials; SES, Summary effect size; wk, week(s)

TABLE S1E. Summary of physical therapy interventions – other

Intervention	n RCTs	N Patients	Start study time poststroke, n RCTs ([H]AR/ER/LR/C)	Intensity of intervention (ranges between studies given only)	Main results meta-analyses	Risk of bias (PEDro) of RCTs (ranges between studies given only)	References
Inspiratory muscle training	2	66	0/0/1/1	30 min/d, 5-7 d/wk, 6-8 wk	Strong evidence (=): maximal inspiratory pressure	4 – 7	[425,426]

=, nonsignficant SES; C, Chronic phase; d, day(s); ER, Early rehabilitation phase; (H)AR, (hyper)acute rehabilitation phase; LR, Late rehabilitation phase; min, minutes; mos, months; RCTs, Randomized controlled trials; SES, Summary effect size; wk, week(s)

TABLE S1F. Summary of physical therapy interventions – intensity of practice

**Intervention**	**n RCTs**	**N Patients**	**Start study time poststroke, n RCTs ([H]AR/ER/LR/C/?)**	**Intensity difference between experimental and control group (mean)**	**Main results meta-analyses**	**Risk of bias (PEDro) of RCTs (ranges between studies given only)**	**References**
Intensity of practice	80	5775	3/48/5/21/3	≈17 h during 10 wk	Strong evidence (+): motor function leg (synergy), motor function arm (synergy), muscle strength leg, comfortable gait speed, maximum gait speed, muscle tone, depression and anxiety, balance, basic ADL, quality of life<br>Strong evidence (=): muscle strength arm, walking distance, mental health patient, falls-efficacy, walking ability, arm-hand activities, extended ADL, falls, mental health caregiver	2 – 8	[1,2,6,10-12,14,17,33,36-38,40,73,88,91,92,94,95,100,101,104,108,111,120,121,123,124,127,129,135,182,237,369,370,374,386,392-394,396,398,400-403,406-411,415,422,427-454]

?, unclear; +, significant positive SES; =, nonsignficant SES; ADL, Activities of daily living; C, Chronic phase; ER, Early rehabilitation phase; (H)AR, (hyper)acute rehabilitation phase; h, hours; LR, Late rehabilitation phase; RCTs, Randomized controlled trials; SES, Summary effect size; wk, weeks

TABLE S1G. Summary of physical therapy interventions – neurological treatment approaches

Intervention	n RCTs	N Patients	Start study time poststroke, n RCTs ([H]AR/ER/LR/C/?)	Control intervention	Main results best-evidence synthesis	Risk of bias (PEDro) of RCTs (ranges between studies given only)	References
NDT versus another intervention	37	1670	0/16/3/17/1	strength training, training balance during various activities, training standing balance with biofeedback – force and position feedback, overground walking, rhythmic auditory cueing, electromechanical-assisted gait training, mCIMT, bilateral arm training, robotics, virtual reality training for the paretic arm, somatosensory training, EMG-BF, strength training, motor relearning programme, orthopaedic programme, movement science-based therapy or problem-willing oriented movement	Strong evidence (=): muscle strength arm, depression<br>Strong evidence (–): motor function (synergy), walking speed, spatiotemporal gait parameters, kinematics arm, arm-hand activities, self-reported arm-hand activities in daily life, basic ADL, quality of life<br>Moderate evidence (=): muscle strength leg, maximal weight bearing paretic leg, coordination, stability shoulder joint, shoulder pain, walking distance, health belief, balance<br>Moderate evidence (–): length of stay<br>Insufficient evidence: grip strength, muscle tone, brain activity, walking ability, extended ADL	4 – 8	[5,35,64,71,109,190,195,196,198,199,202,205,208,210,211,229-231,235,244,246,247,253,262,268,300,360,385,396,448,455-462]
NDT versus NDT + another intervention	33	1106	0/19/7/8/1	NDT plus: training standing balance with biofeedback – force and position feedback, training sitting balance, training standing balance, training balance during various activities, overground walking, community walking, rhythmic auditory cueing, treadmill training, BWSTT, electromechanical-assisted gait training, aerobic training, robotics, virtual reality arm, NMS, EMG-NMS, TENS, EMG-BF or passive movement of the wrist with an apparatus	Strong evidence (=): kinematics gait<br>Strong evidence (–): motor function (synergy), muscle strength arm, gait speed, spatiotemporal gait parameters, muscle tone, range of motion, balance, walking ability, arm-hand activities, basic ADL<br>Moderate evidence (=): symmetry (sitting, standing, sit-to-stand and wile reaching), depression, stand-to-sit, sit-to-stand<br>Moderate evidence (–): muscle strength leg, walking distance, coordination, EMG contraction, shoulder subluxation, neglect, aerobic capacity	2 – 8	[4,6,15,26,28,33,48,49,77,79,100,102,105,111,132,142,146,149,167,179,258,287,318,324,329,330,344,350,364,449,463,464]
Augmented NDT versus NDT (intensity difference)	6	786	0/5/0/0/1	NDT	Strong evidence (=): strength, walking ability, arm-hand activities, basic ADL, extended ADL<br>Moderate evidence (+): motor function (synergy), range of motion<br>Moderate evidence (=): pain, depression, balance, sit-to-stand, handicap, quality of life	6 – 8	[432,437,439,445,446,448]

+, significant positive effect; =, nonsignficant effect; –, significant negative effect; ?, unclear; ADL, Activities of daily living; BWSTT, Body-weight supported treadmill training; C, Chronic phase; EMG-BF, Electromyographic biofeedback; EMG, Electromyograpic; EMG-NMS, Electromyography-triggered neuromuscular stimulation; ER, Early rehabilitation phase; (H)AR, (hyper)acute rehabilitation phase; LR, Late rehabilitation phase; mCIMT, modified Constraint-induced movement therapy; NDT, Neurodevelopmental treatment; NMS, Neuromuscular stimulation; RCTs, Randomized controlled trials; SES, Summary effect size; TENS, Transcutaneous electrical nerve stimulation

TABLE S2A. Summary of the evidence for physical therapy interventions – gait and mobility-related functions and activities

Intervention	Pooling possible for:	Outcome measures	N (pooling)	Type effects model	SES (95%CI) Hedges' *g*	Heterogeneity I^2 (%)	Statistical power	Difference between poststroke phases
Early mobilization	a. complications	a. complications	a. 210	a. Fixed	a. 0.090 (-0.484, 0.663)	a. 0	a. 0.088	a. NA
	b. neurological deterioration	b. deterioration (5 d, 0-7d)	b. 103	b. Fixed	b. -0.338 (-0.828, 0.152)	b. 0	b. 0.317	b. NA
	early poststroke	c. Borg RPE	c. 103	c. Fixed	c. 0.178 (-0.204, 0.561)	c. 0	c. 0.123	c. NA
	c. fatigue	d. mRS	d. 103	d. Fixed	d. 0.376 (-0.071, 0.824)	d. 4	d. 0.378	d. NA
	d. basic ADL	e. discharge home	e. 103	e. Fixed	e. 0.276 (-0.216, 0.768)	e. 0	e. 0.229	e. NA
	e. discharge home							
Sitting balance training	a. symmetry while sitting	a. symmetry sitting	a. 59	a. Fixed	a. -0.141 (-0.715, 0.433)	a. 26	a. 0.074	a. NA
	b. symmetry while standing	b. symmetry standing	b. 59	b. Fixed	b. -0.333 (-0.911, 0.246)	b. 38	b. 0.189	b. NA
*, only RCTs investigating	c. ground reaction force*	c. GRF	c. 50	c. Random	c. 4.180 (-0.171, 8.532)	c. 94	c. 1.000	c. NS
reaching beyond arm's	d. hand movement time*	d. hand movement time reaching	d. 50	d. Fixed	d. 1.751 (1.114, 2.388)	d. 0	d. 0.996	d. NS
length while sitting	e. sitting balance	e. reach distance, sitting equilibrium test	e. 70	e. Random	e. 1.726 (-0.093, 3.546)	e. 90	e. 1.000	e. S = ER:
	f. sitting balance*	f. reach distance, sitting equilibrium test	f. 50	f. Random	f. 2.472 (0.836, 4.108)	f. 77	f. 1.000	beyond arm's
	g. walking ability	g. BI mobility, FAC	g. 59	g. Fixed	g. -0.081 (-0.617, 0.455)	g. 0	g. 0.058	length
	h. basic ADL	h. BI, FIM	h. 59	h. Fixed	h. -0.237 (-0.775, 0.300)	h. 0	h. 0.119	f. NS
								g. NA
								h. NA
Sit-to-stand training	a. body weight distribution	a. BWD sitting, BWD rising, BWD sitting down	a. 13	a. Random	a. 0.264 (-0.474, 1.002)	a. 77	a. 0.391	a. NS
	b. sit-to-stand	b. STS	b. 50	b. Fixed	b. 0.405 (-0.138, 0.948)	b. 0	b. 0.359	b. NS
Standing balance training	a. postural sway	a. posturography	a. 42	a. Fixed	a. 0.225 (-0.364, 0.815)	a. 33	a. 0.099	a. NS
without biofeedback	b. balance	b. BBS, MAS* sitting	b. 149	b. Fixed	b. -0.126 (-0.444, 0.193)	b. 0	b. 0.102	b. NA
	c. sit-to-stand	c. MAS* STS, STS	c. 149	c. Fixed	c. 0.060 (-0.259, 0.378)	c. 0	c. 0.062	c. NA
	d. walking ability	d. MAS* STS, RMI, RMA, RMA GF, TCT	d. 148	d. Fixed	d. 0.116 (-0.203, 0.435)	d. 0	d. 0.134	d. NS
Standing balance training	a. motor function leg (synergy)	a. FMA, Brunnstrom stages	a. 97	a. Fixed	a. 0.121 (-0.269, 0.511)	a. 0	a. 0.081	a. NS
with biofeedback – force	b. comfortable gait speed	b. 10MWT, WS	b. 184	b. Fixed	b. -0.220 (-0.504, 0.063)	b. 0	b. 0.248	b. NS
and position feedback	c. cadence	c. gait analysis	c. 67	c. Fixed	c. -0.042 (-0.511, 0.427)	c. 0	c. 0.053	c. NS
	d. step length	d. gait analysis	d. 67	d. Fixed	d. 0.062 (-0.407, 0.530)	d. 0	d. 0.056	d. NS
	e. mono and bipedal phase	e. DST, SST	e. 67	e. Fixed	e. -0.121 (-0.595, 0.353)	e. 0	e. 0.072	e. NS
	f. postural sway	f. posturography	f. 151	f. Fixed	f. 0.366 (0.049, 0.683)	f. 0	f. 0.497	f. S
	phase: ER	g. BBS, FMA balance, PASS, standing affected leg,	121	Fixed	0.209 (-0.140, 0.559)	0	0.168	
	phase: C (n=1)	standing nonaffected leg	30	Fixed	1.090 (0.339, 1.842)	0	0.676	
	g. balance	h. FAC, FIM locomotion, stair climbing, walking, RMA	g. 186	g. Fixed	g. -0.208 (-0.489, 0.072)	g. 0	g. 0.228	g. NS
	h. walking ability	GF, RMI, TUG	h. 251	h. Random	h. -0.010 (-0.432, 0.413)	h. 60	h. 0.051	h. NS
	i. basic ADL	i. BI, FIM, NEADL, FIM motor	i. 191	i. Fixed	i. -0.048 (-0.328, 0.232)	i. 34	i. 0.060	i. NS
Balance training during	a. comfortable gait speed	a. 10MWT, WS	a. 88	a. Fixed	a. 0.313 (-0.100, 0.726)	a. 0	a. 0.244	a. NA
various activities	b. falls-efficacy	b. ABC, FES-I	b. 82	b. Fixed	b. 0.181 (-0.247, 0.609)	b. 0	b. 0.111	b. NS
	c. balance	c. BBA, BBS, FR, LRT, static standing, step reaction	c. 397	c. Random	c. 0.355 (0.067, 0.642)	c. 51	c. 0.863	c. NS
	d. walking ability	time, ST, TIS	d. 271	d. Fixed	d. 0.137 (-0.099, 0.374)	d. 24	d. 0.162	d. NS
	e. basic ADL	d. MAS* walking, STS, stand-to-sit, stair climb, Tinetti	e. 211	e. Fixed	e. 0.383 (0.113, 0.653)	e. 48	e. 0.660	e. NS
	f. quality of life	gait, TUG	f. 252	f. Fixed	f. -0.149 (-0.393, 0.096)	f. 8	f. 0.176	f. NS
		e. BI, FIM						
		f. NHP, QoL, SA-SIP30, SF-36						
Body-weight supported	a. motor function leg (synergy)	a. FMA	a. 448	a. Fixed	a. -0.194 (-0.387, 0.000)	a. 12	a. 0.433	a. NS
treadmill training	b. comfortable gait speed	b. 10MWT, WS	b. 858	b. Random	b. 0.468 (0.107, 0.829)	b. 82	b. 1.000	b. NS
	c. maximum gait speed	c. 10MWT, WS	c. 179	c. Fixed	c. 0.245 (-0.041, 0.532)	c. 0	c. 0.298	c. NS
	d. walking distance	d. 12MWT, 6MWT, walking distance	d. 832	d. Random	d. 0.606 (0.173, 1.039)	d. 87	d. 1.000	d. NS
	e. cadence	e. gait analysis	e. 56	e. Fixed	e. -0.285 (-0.793, 0.222)	e. 25	e. 0.147	e. NS
	f. stride length	f. gait analysis	f. 142	f. Fixed	f. 0.148 (-0.173, 0.470)	f. 0	f. 0.118	f. NS
	g. aerobic capacity	g. VO_2max	g. 32	g. Fixed	g. 0.631 (-0.051, 1.314)	g. 40	g. 0.327	g. NS
	h. energy expenditure	h. O_2-cost	h. 33	h. Fixed	h. 0.563 (-0.102, 1.228)	h. 0	h. 0.273	h. NS
	i. balance	i. BBS, FMA balance	i. 540	i. Random	i. 0.609 (-0.118, 1.336)	i. 92	i. 1.000	i. NS
	j. walking ability	j. FAC, FIM locomotion, FIM gait, RMA GF, RMA LT,	j. 357	j. Fixed	j. 0.215 (0.000, 0.431)	j. 42	j. 0.422	j. NS
	k. quality of life	RMI, TCT, TUG, SPPB	k. 514	k. Fixed	k. 0.054 (-0.126, 0.234)	k. 0	k. 0.083	k. NS
		k. AAP, SA-SIP30, SIS, LLFDI						
Electromechanical-	a. neurological functions	a. CNS, NIHSS	a. 115	a. Random	a. 0.192 (-0.492, 0.877)	a. 67	a. 0.144	a. NA
assisted gait training	b. motor function leg (synergy)	b. FMA	b. 53	b. Random	b. 0.028 (-1.074, 1.129)	b. 73	b. 0.051	b. NS
	c. muscle strength	c. MI leg, MRC	c. 313	c. Fixed	c. 0.209 (-0.012, 0.429)	c. 35	c. 0.358	c. NS
*, only for dependent	d. comfortable gait speed*	d. 10MWT, 8MWT, WS	d. 122	d. Random	d. 0.261 (-0.300, 0.821)	d. 58	d. 0.235	d. S
patients	*phase: ER (dependent)*	e. 10MWT, 5MWT, WS	38	Fixed	0.983 (0.319, 1.647)	0	0.699	
	phase: C (independent)	f. 3MWT, 6MWT	64	Fixed	-0.066 (-0.547, 0.415)	0	0.056	
	Dependent & independent	g. gait analysis	20	Fixed	0.014 (-0.826, 0.853)	0	0.050	

	e. maximum gait speed	h. gait analysis	e. 383	e. Fixed	e. 0.215 (0.016, 0.413)	e. 0	e. 0.439	e. NS
	f. walking distance	i. HR rest	f. 394	f. Fixed	f. 0.246 (0.051, 0.442)	f. 8	f. 0.566	f. NS
	g. cadence	j. HR peak	g. 163	g. Random	g. -0.058 (-0.811, 0.695)	g. 83	g. 0.062	g. S
	phase: ER	k. BBS, DB, SST affected leg, SB, Tinetti balance, ST	60	Fixed	0.403 (-0.098, 0.904)	21	0.268	
	phase: LR	l. EMS, EU walking scale, FAC, mEFAP, RMA GF,	63	Fixed	-1.021 (-1.654, -0.388)	0	0.915	
	phase: C	RMA LT, RMI, SAS disability, SPPB, TCT, TMS	40	Fixed	0.068 (-0.540, 0.676)	0	0.141	
	h. step length	m. BI, FIM, FIM motor	h. 68	h. Fixed	h. 0.177 (-0.234, 0.589)	h. 0	h. 0.108	h. NS
	i. heart rate rest	n. SF-36, LLFDI	i. 95	i. Fixed	i. -0.320 (-0.719, 0.079)	i. 0	i. 0.480	i. NA
	j. peak heart rate		j. 95	j. Fixed	j. 0.520 (0.116, 0.923)	j. 0	j. 0.869	j. NA
	k. balance		k. 320	k. Random	k. -0.039 (-0.603, 0.525)	k. 84	k. 0.061	k. S
	phase: ER		123	Fixed	0.484 (0.131, 0.836)	0	0.620	
	phase: LR (n=1)		63	Fixed	-1.926 (-2.519, -1.333)	0	1.000	
	phase: C		134	Fixed	0.004 (-0.329, 0.336)	0	0.050	
	l. walking ability*		l. 669	l. Random	l. 0.186 (-0.329, 1.333)	l. 82	l. 0.550	l. S
	phase: ER (dependent)		502	Fixed	0.421 (0.237, 0.605)	0	0.982	
	phase: LR (independent)		63	Fixed	-2.245 (-2.873, -1.487)	0	1.000	
	phase: C (independent)		104	Fixed	-0.051 (-0.428, 0.326)	0	0.437	
	m. basic ADL		m. 403	m. Fixed	m. 0.433 (0.237, 0.630)	m. 39	m. 0.960	m. NS
	n. quality of life		n. 112	n. Random	n. 0.122 (-0.594, 0.838)	n. 71	n. 0.087	n. NS
Electromechanical-	a. maximum gait speed	a. 10MWT, 5MWT	a. 65	a. Random	a. 0.496 (-0.782, 1.774)	a. 85	a. 0.395	a. S
assisted gait training with	*phase: ER (dependent)*	b. BBS, DB, SB	35	Fixed	1.150 (0.442, 1.857)	0	0.791	
functional	*phase: C (independent)*	c. EMS, FAC	30	Fixed	-0.154 (-0.852, 0.543)	0	0.065	
electrostimulation	b. balance	d. BI, FIM	b. 102	b. Fixed	b. 0.397 (0.008, 0.786)	b. 44	b. 0.682	b. NS
* only early rehabilitation	c. walking ability*		c. 72	c. Fixed	c. 1.060 (0.570, 1.551)	c. 0	c. 0.955	c. NA
phase	d. basic ADL		d. 102	d. Random	d. 0.137 (-0.669, 0.943)	d. 76	d. 0.092	d. NS
Speed dependent	a. comfortable gait speed	a. 10MWT, WS	a. 192	a. Fixed	a. -0.010 (-0.292, 0.273)	a. 0	a. 0.050	a. NS
treadmill training (without	b. maximum gait speed	b. 10MWT, WS	b. 304	b. Fixed	b. 0.236 (0.009, 0.462)	b. 0	b. 0.428	b. NS
body-weight support)	c. gait speed endurance	c. 6MWT	c. 110	c. Fixed	c. -0.038 (-0.407, 0.331)	c. 0	c. 0.053	c. NA
	d. walking distance	d. 6MWT, walking endurance	d. 213	d. Random	d. 0.143 (-0.126, 0.412)	d. 0	d. 0.149	d. NS
	e. cadence	e. gait analysis	e. 190	e. Fixed	e. 0.104 (-0.184, 0.393)	e. 9	e. 0.097	e. NS
	f. step width	f. gait analysis	f. 82	f. Fixed	f. 0.567 (0.135, 0.999)	f. 0	f. 0.598	f. NA
	g. stride length	g. gait analysis	g. 221	g. Fixed	g. 0.234 (-0.034, 0.502)	g. 15	g. 0.328	g. NS
	h. aerobic capacity	h. VO_2max, VO_2peak	h. 178	h. Fixed	h. 0.243 (-0.049, 0.536)	h. 0	h. 0.291	h. NA
	i. balance	i. BBS, balance	i. 44	i. Fixed	i. -0.325 (-0.899, 0.249)	i. 0	i. 0.153	i. NS
	j. walking ability	j. FAC, RMI, STS, S-test complex gait, gait	j. 133	j. Random	j. 0.632 (-0.126, 1.391)	j. 73	j. 0.877	j. NS
Overground walking	a. comfortable gait speed	a. 10MWT, 5MWT, WS	a. 541	a. Random	a. 0.267 (-0.019, 0.554)	a. 56	a. 0.771	a. NS
	b. maximum gait speed	b. 10MWT, 5MWT	b. 159	b. Fixed	b. 0.238 (-0.074, 0.550)	b. 0	b. 0.255	b. NS
* independent patients	c. walking distance	c. 6MWT, walking distance	c. 407	c. Fixed	c. -0.008 (-0.203, 0.186)	c. 38	c. 0.051	c. S
** dependent patients	*phase: ER (dependent)*	d. gait analysis	139	Fixed	-0.379 (-0.710, -0.047)	0	0.476	
	phase: C (independent)	e. gait analysis	223	Fixed	0.278 (0.015, 0.540)	0	0.428	
	d. cadence	f. gait analysis	d. 113	d. Random	d. 0.105 (-1.053, 1.264)	d. 88	d. 0.920	d. S
	phase: LR	g. gait analysis	88	Random	0.646 (-0.177, 1.469)	69	0.726	
	phase: C (n=1)	h. falls	25	Fixed	-0.974 (-1.779, -0.168)	0	0.530	
	e. stride length	i. VO_2max, VO_2peak	e. 120	e. Random	e. 0.588 (-0.362, 1.538)	e. 82	e. 0.781	e. S
	phase: ER	j. HR peak	70	Fixed	-0.212 (-0.678, 0.255)	0	0.120	
	phase: LR (n=1)	k. diastolic BP	25	Fixed	0.986 (0.180, 1.793)	0	0.539	
	phase: C (n=1)	l. systolic BP	25	Fixed	1.136 (0.314, 1.957)	0	0.644	
	f. stride time	m. HADS anxiety	f. 50	f. Fixed	f. 0.309 (-0.232, 0.850)	f. 0	f. 0.160	f. NS
	g. symmetry gait pattern	n. HADS depression	g. 50	g. Fixed	g. 0.374 (-0.167, 0.916)	g. 0	g. 0.213	g. NS
	h. number of falls*	o. BBS, DB, SB	h. 256	h. Fixed	h. 0.052 (-0.260, 0.364)	h. 0	h. 0.065	h. NS
	i. aerobic capacity**	p. BI walking, FAC, FIM locomotion, RMA GF, RMA	i. 49	i. Fixed	i. -0.645 (-1.215, -0.093)	i. 24	i. 0.513	i. NA
	j. peak heart rate*	LT, RMI, STREAM mobility, TCT, TUG, ST	j. 49	j. Fixed	j. -0.529 (-1.084, 0.027)	j. 14	j. 0.369	j. NA
	k. diastolic blood pressure*	q. BI, FIM	k. 49	k. Fixed	k. -0.051 (-0.593, 0.490)	k. 0	k. 0.053	k. NA
	l. systolic blood pressure*	r. AAP, FAI, mRS, NEADL	l. 49	l. Fixed	l. -0.451 (-1.005, 0.103)	l. 44	l. 0.286	l. NA
	m. anxiety*		m. 224	m. Fixed	m. 0.356 (0.093, 0.619)	m. 0	m. 0.630	m. NA
	n. depression*		n. 224	n. Fixed	n. 0.232 (-0.029, 0.494)	n. 0	n. 0.323	n. NA
	o. balance		o. 262	o. Random	o. 0.462 (-0.261, 1.184)	o. 86	o. 0.890	o. S
	phase: LR (n=1)		63	Fixed	1.926 (1.333, 2.519)	0	1.000	
	phase: C		199	Fixed	0.137 (-0.141, 0.415)	0	0.134	
	p. walking ability		p. 576	p. Random	p. 0.266 (-0.171, 0.702)	p. 82	p. 0.787	p. S
	phase: ER		144	Random	-0.086 (-0.664, 0.493)	67	0.073	
	phase: LR (n=1)		63	Fixed	2.245 (1.348, 3.142)	0	1.000	
	phase: C		369	Fixed	0.225 (0.011, 0.439)	0	0.464	
	q. basic ADL		q. 362	q. Fixed	q. -0.076 (-0.282, 0.130)	q. 41	q. 0.096	q. NS
	r. extended ADL		r. 391	r. Random	r. -0.228 (-0.554, 0.009)	r. 56	r. 0.588	r. NS

Rhythmic gait cueing	a. gait speed	a. WS	a. 118	a. Random	a. 0.543 (-1.831, 2.918)	a. 97	a. 0.717	a. NA
	b. cadence	b. gait analysis	b. 118	b. Random	b. 0.633 (-1.799, 3.066)	b. 97	b. 0.835	b. NA
	c. stride length	c. gait analysis	c. 118	c. Random	c. 0.154 (-1.393, 1.700)	c. 94	c. 0.113	c. NA
	d. symmetry gait pattern	d. gait analysis	d. 118	d. Random	d. 1.012 (-0.908, 2.933)	d. 96	d. 0.995	d. NA
Community walking	a. maximum gait speed	a. 10MWT	a. 64	a. Random	a. 0.301 (-0.824, 1.426)	a. 80	a. 0.263	a. NA
	b. walking distance	b. 6MWT	b. 94	b. Fixed	b. 0.155 (-0.243, 0.553)	b. 13	b. 0.101	b. NS
	c. balance confidence	c. ABC	c. 55	c. Fixed	c. 0.346 (-0.173, 0.865)	c. 0	c. 0.200	c. NS
Virtual reality mobility training	a. comfortable gait speed	a. 10MWT, WS	a. 42	a. Random	a. -0.466 (-1.348, 0.415)	a. 54	a. 0.262	a. NA
	b. maximum gait speed	b. 10MWT, WS, top speed	b. 58	b. Fixed	b. 0.213 (-0.284, 0.710)	b. 0	b. 0.110	b. NA
	c. step length	c. gait analysis	c. 42	c. Fixed	c. 0.185 (-0.402, 0.772)	c. 31	c. 0.083	c. NA
	d. walking ability	d. FAC, WAQ, MMAS* walking, MMAS* balance and gait	d. 30	d. Fixed	d. 0.165 (-0.514, 0.844)	d. 0	d. 0.069	d. NA
Circuit class training (gait and mobility-related functions and activities)	a. muscle strength	a. strength flexor/extensor: ankle, hip, knee	a. 177	a. Random	a. 0.430 (-0.007, 0.868)	a. 53	a. 0.425	a. NS
	b. gait speed	b. 10MWT, WS	b. 181	b. Random	b. 0.477 (-0.006, 0.960)	b. 58	b. 0.790	b. NS
	c. walking distance	c. 6MWT	c. 208	c. Fixed	c. 0.568 (0.295, 0.841)	c. 0	c. 0.938	c. NS
	d. number of falls	d. falls	d. 82	d. Fixed	d. 0.065 (-0.389, 0.518)	d. 0	d. 0.058	d. NS
	e. falls-efficacy	e. ABC, FES-I	e. 140	e. Fixed	e. 0.316 (-0.014, 0.646)	e. 0	e. 0.359	e. NS
	f. depression	f. GDS-15, HADS depression	f. 99	f. Random	f. 0.239 (-0.654, 1.132)	f. 78	f. 0.178	f. NA
	g. balance	g. BBS, FR, ST	g. 298	g. Fixed	g. 0.299 (0.075, 0.524)	g. 0	g. 0.599	g. NS
	h. walking ability	h. RMI, TUG	h. 259	h. Fixed	h. 0.278 (0.037, 0.520)	h. 0	h. 0.496	h. NS
	i. basic ADL	i. BI, FIM	i. 100	i. Fixed	i. 0.139 (-0.250, 0.527)	i. 27	i. 0.092	i. NS
	j. extended ADL	j. FAI, NEADL	j. 100	j. Fixed	j. -0.134 (-0.284, 0.359)	j. 0	j. 0.089	j. NA
	k. physical activity	k. PADS, PASIPD, activity monitor	k. 121	k. Fixed	k. 0.538 (0.401, 0.758)	k. 0	k. 0.709	k. NS
	l. quality of life	l. NHP, SF-36	l. 147	l. Fixed	l. 0.038 (-0.284, 0.359)	l. 0	l. 0.055	l. NS
Caregiver-mediated exercises (gait and mobility-related functions and activities)	a. basic ADL	a. BI	a. 340	a. Fixed	a. 0.389 (0.135, 0.625)	a. 0	a. 0.869	a. NA
	b. extended ADL	b. NEADL, FAI	b. 340	b. Random	b. 0.135 (-0.441, 0.712)	b. 69	b. 0.190	b. NA
	c. caregiver strain	c. CSI, CBS	c. 340	c. Fixed	c. 0.517 (0.301, 0.732)	c. 0	c. 0.982	c. NA
Orthosis for walking	a. comfortable gait speed	a. WS	a. 111	a. Random	a. 0.596 (-0.550, 1.742)	a. 87	a. 0.770	a. NS
Water-based exercises	a. muscle strength	a. strength: knee, leg	a. 32	a. Fixed	a. 0.779 (0.089, 1.468)	a. 0	a. 0.741	a. NA
	b. balance	b. BBS	b. 32	b. Random	b. 0.599 (-0.622, 1.821)	b. 67	b. 0.529	b. NA
Interventions for somatosensory functions of the paretic leg	a. motor function leg (synergy)	a. FMA, Brunnstrom stages	a. 63	a. Random	a. 1.017 (-0.024, 2.058)	a. 75	a. 0.913	a. NA
	b. gait speed	b. 10MWT, WS	b. 51	b. Fixed	b. -0.127 (-0.659, 0.405)	b. 0	b. 0.068	b. NS
	c. balance	c. BBS	c. 54	c. Random	c. 1.243 (-0.255, 2.741)	c. 85	c. 0.958	c. NS
Electrostimulation of the paretic leg								
– NMS	a. motor function leg (synergy)	a. FMA, Brunnstrom stages	a. 76	a. Fixed	a. 0.448 (0.004, 0.891)	a. 0	a. 0.395	a. NS
	b. muscle tone	b. CSS, H-reflex, MAS, SI	b. 259	b. Fixed	b. 0.274 (0.034, 0.514)	b. 36	b. 0.479	b. NS
	c. muscle strength	c. strength: flexion/extension knee, ankle	c. 133	c. Fixed	c. 0.384 (0.045, 0.722)	c. 28	c. 0.469	c. NS
	d. active range of motion ankle	d. ROM	d. 112	d. Fixed	d. 0.284 (-0.088, 0.656)	d. 0	d. 0.247	d. NS
	e. active range of motion knee	e. ROM	e. 57	e. Random	e. 0.064 (-0.891, 1.018)	e. 64	e. 0.055	e. NA
	f. gait speed	f. 10MWT, 50MWT, EFAP floor, WS	f. 215	f. Random	f. 0.342 (-0.082, 0.766)	f. 59	f. 0.581	f. NS
	g. cadence	g. gait analysis	g. 70	g. Fixed	g. 0.131 (-0.340, 0.601)	g. 0	g. 0.076	g. NS
	h. step length	h. gait analysis	h. 77	h. Fixed	h. -0.305 (-0.757, 0.146)	h. 0	h. 0.217	h. NS
	i. symmetry gait	i. gait analysis	i. 40	i. Fixed	i. -0.074 (-0.671, 0.524)	i. 0	i. 0.065	i. NS
	j. balance	j. BBS, balance	j. 125	j. Fixed	j. 0.206 (-0.140, 0.551)	j. 40	j. 0.174	j. NS
	k. walking ability	k. EFAP up and go, FAC, MGH FAC, RMI, TCT, TUG	k. 237	k. Random	k. 0.132 (-0.303, 0.568)	k. 65	k. 0.141	k. NS
	l. basic ADL	l. BI	l. 114	l. Fixed	l. 0.293 (-0.068, 0.655)	l. 25	l. 0.063	l. NA
– EMG-NMS	a. muscle tone	a. SI, MAS	a. 68	a. Fixed	a. 0.368 (-0.101, 0.836)	a. 0	a. 0.257	a. NS
	b. basic ADL	b. BI, FIM	b. 68	b. Fixed	b. 0.351 (-0.118, 0.820)	b. 0	b. 0.238	b. NS
– TENS	a. muscle strength	a. strength: flexion/extension ankle, knee	a. 135	a. Fixed	a. 0.471 (0.115, 0.827)	a. 0	a. 0.651	a. NS
	b. muscle tone	b. CSS, MAS	b. 178	b. Random	b. 0.357 (-0.715, 1.430)	b. 86	b. 0.540	b. NS
	c. active range of motion ankle plantar flexion	c. ROM	c. 91	c. Fixed	c. 0.245 (-0.174. 0.664)	c. 0	c. 0.171	c. NS
		d. ROM	d. 91	d. Random	d. 0.053 (-0.621, 0.727)	d. 61	d. 0.055	d. NS
	d. active range of motion ankle dorsiflexion	e. WS	e. 170	e. Fixed	e. 0.232 (-0.052, 0.516)	e. 0	e. 0.257	e. NS
		f. 6MWT	f. 79	f. Fixed	f. 0.451 (-0.019, 0.921)	f. 0	f. 0.408	f. NA
	e. gait speed	g. BI mobility, TUG	g. 195	g. Fixed	g. 0.616 (0.320, 0.911)	g. 0	g. 0.958	g. NS
	f. walking distance							
	g. walking ability							
Electromyographic biofeedback for the paretic leg	a. range of motion ankle dorsiflexion	a. ROM	a. 103	a. Fixed	a. -0.011 (-0.399, 0.376)	a. 0	a. 0.050	a. NS
		b. ROM	b. 52	b. Random	b. 0.192 (-0.754, 1.138)	b. 61	b. 0.095	b. NA
	b. range of motion knee flexion	c. gait analysis	c. 87	c. Fixed	c. 0.416 (-0.005, 0.838)	c. 0	c. 0.000	c. NS
	c. step length	d. WS	d. 34	d. Random	d. 0.514 (-0.444, 1.472)	d. 52	d. 0.247	d. NA
	d. gait speed	e. EMG registration	e. 50	e. Fixed	e. 0.268 (-0.284, 0.821)	e. 0	e. 0.000	e. NS
	e. EMG activity							

10MWT, 10-meter walk test; 12MWT, 12-minute walk test; 3MWT, 3-minute walk test; 5MWT, 5-meter walk test; 6MWT, 6-minute walk test; 8MWT, 8-meter walk test; AAP, Adelaide activities profile; ABC, Activities-specific balance confidence scale; ADL, Activities of daily living; BBA, Brunel balance assessment; BBS, Berg balance scale; BI, Barthel index; BP, Blood pressure; BWD, Body-weight distribution; C, Chronic phase; CI, Confidence interval; CSS, Composite spasticity scale; CNS, Canadian neurological scale; DB, Dynamic balance; DST, Double support time; EFAP, Emory functional ambulation profile; EMS, Elderly mobility scale; ER, Early rehabilitation phase; FAC, Functional ambulation categories; FAI, Frenchay activities index; FES-I, Falls-efficacy scale; FIM, Functional independence measure; FMA, Fugl-meyer assessment; FR, Functional reach; FSST, Four square step test; GDS-15, Geriatric depression scale - 15;GRF, Ground reaction force; HADS, Hospital anxiety and depression scale; HR, Heart rate; LLFDI, Late life function and disability instrument; LR, Late rehabilitation phase; LRT, Lateral reach test; MAS, Modified ashworth scale; MAS*, Motor assessment scale; mEFAP, modified Emory functional ambulation profile; MI, Motricity index; MMAS*, modified Motor assessment scale; MRC, Medical research council; mRS, modified Rankin scale; NA, Not applicable; NEADL, Nottingham extended ADL index; NHP, Nottingham health profile; NIHSS, National institutes of health stroke scale; NS, Not significant; PADS, Physical activity and disability scale; PASIPD, Physical activity scale for individuals with physical disabilities; PASS, Postural assessment scale for stroke; QoL, Quality of life; RMI, Rivermead mobility index; RMA, Rivermead motor assessment; RMA GF, RMA gross function; RMA LT, RMA leg and trunk; ROM, Range of motion; RPE, Rating of perceived exertion; S, Significant; SA-SIP30, Stroke-adapted 30-item version of the sickness impact profile; SAS, Stroke activities scale; SES, Summary effect size; SF-36, 36-Item short form health survey; SB, Static balance; SI, Spasticity index; SIS, Stroke impact scale; SPPB, Short physical performance battery; SST, Single-support time; ST, Step test; STREAM, Stroke rehabilitation assessment of movement instrument; STS, Sit-to-stand; TCT, Trunk control test; TIS, Trunk impairment scale; TMS, Toulouse motor scale; TUG, Timed up and go test; WAQ, Walking ability questionnaire; WD, Walking distance; WQ, Walking quality; WS, Walking speed gait analysis

TABLE S2B. Summary of the evidence for physical therapy interventions – arm-hand activities

Intervention	Pooling possible for:	Outcome measures	N (pooling)	Type effects model	SES (95%CI) Hedges' *g*	Heterogeneity I^2 (%)	Statistical power	Difference between poststroke phases
Therapeutic positioning of the paretic arm	a. passive range of motion shoulder external rotation	a. ROM	a. 132	a. Fixed	a. 0.171 (0.031, 0.700)	a. 0	a. 0.135	a. NA
		b. ROM	b. 72	b. Random	b. -0.065 (-1.097, 0.967)	b. 69	b. 0.057	b. NA
	b. passive range of motion shoulder internal rotation	c. ROM	c. 55	c. Fixed	c. 0.366 (-0.153, 0.885)	c. 0	c. 0.218	c. NA
		d. VAS	d. 60	d. Random	d. 0.114 (-0.869, 1.097)	d. 74	d. 0.067	d. NA
	c. contracture shoulder	e. VAS	e. 60	e. Random	e. -0.332 (-1.067, 0.404)	e. 54	e. 0.198	e. NA
	d. pain (rest)	f. BI, mBI	f. 49	f. Fixed	f. -0.156 (-0.700, 0.388)	f. 0	f. 0.082	f. NA
	e. pain (movement)							
	f. basic ADL							
Reflex-inhibiting positions and immobilization techniques for the paretic wrist and hand	a. muscle tone	a. MAS, Tardieu, resistance	a. 93	a. Fixed	a. 0.089 (-0.321, 0.499)	a. 0	a. 0.067	a. NS
	b. pain	b. pain	b. 62	b. Random	b. -0.582 (-2.200, 1.037)	b. 81	b. 0.507	b. NA
	c. passive range of motion	c. ROM	c. 93	c. Fixed	c. -0.088 (-0.523, 0.347)	c. 34	c. 0.066	c. NA
Air-splints around the paretic arm	a. motor function arm (synergy)	a. FMA	a. 205	a. Random	a. 0.036 (-0.496, 0.567)	a. 68	a. 0.056	a. NS
		b. MAS	b. 167	b. Random	b. -0.104 (-0.839, 0.632)	b. 79	b. 0.090	b. S
	b. muscle tone	c. NSA, FMA	144	Fixed	-0.495 (-0.841, -0.149)	0	0.718	
	phase: ER	d. VAS, FMA	22	Fixed	0.914 (0.082, 1.746)	0	0.424	
	phase: LR	e. ARAT, TEMPA	c. 41	c. Random	c. 0.305 (-1.078, 1.687)	c. 80	c. 0.132	c. NS
	c. somatosensory function		d. 41	d. Random	d. -0.367 (-1.431, 0.698)	d. 67	d. 0.170	d. NS
	d. pain		e. 180	e. Fixed	e. 0.001 (-0.296, 0.298)	e. 0	e. 0.050	e. NA
	e. arm-hand activities							
Supportive techniques or devices for the prevention or treatment of glenohumeral subluxation and/or hemiplegic shoulder pain	a. motor function arm	a. MAS*	a. 140	a. Fixed	a. -0.186 (-0.517, 0.145)	a. 20	a. 0.162	a. NA
	b. pain	b. VAS, pain free days	b. 130	b. Random	b. 0.274 (-0.539, 1.086)	b. 67	b. 0.274	b. NA
Bilateral arm training	a. motor function arm (synergy)	a. FMA, MSS	a. 274	a. Random	a. 0.194 (-0.359, 0.746)	a. 80	a. 0.281	a. NS
		b. GS, PS, strength: elbow, shoulder, WFMT weight	b. 163	b. Fixed	b. -0.018 (-0.321, 0.285)	b. 0	b. 0.051	b. NS
	b. muscle strength	c. ARAT, BBT, PPT, TEMPA, WMFT	c. 417	c. Fixed	c. 0.035 (-0.157, 0.226)	c. 40	c. 0.061	c. NS
	c. arm-hand activities	d. MAL	d. 241	d. Random	d. -0.030 (-0.418, 0.357)	d. 54	d. 0.061	d. NA
	d. self-reported amount of arm-hand use daily life	e. FIM, BI, mBI	e. 245	e. Fixed	e. -0.054 (-0.305, 0.197)	e. 0	e. 0.065	e. NS
	e. basic ADL							
(modified) Constraint-induced movement therapy								
– Original CIMT	a. arm-hand activities (n=1)	a. WMFT	a. 222	a. Fixed	a. 0.538 (0.271, 0.806)	a. 0	a. 0.927	a. NA
	b. self-reported amount of arm-hand use in daily life (n=1)	b. MAL	b. 222	b. Fixed	b. 0.671 (0.402, 0.941)	b. 0	b. 0.988	b. NA
	c. self-reported quality of arm-hand movement in daily life (n=1)	c. MAL	c. 222	c. Fixed	c. 0.807 (0.534, 1.080)	c. 0	c. 0.999	c. NA
– High-intensity mCIMT	a. motor function arm (synergy)	a. FMA	a. 50	a. Random	a. 0.211 (-0.763, 1.185)	a. 67	a. 0.097	a. S
		b. ARAT, WMFT	28	Random	1.032 (-0.361, 2.425)	62	0.604	
	phase: ER	c. MAL	22	Fixed	-0.566 (-1.351, 0.219)	0	0.218	
	phase: C	d. MAL	b. 348	b. Fixed	b. 0.304 (0.098, 0.511)	b. 11	b. 0.676	b. NS
	b. arm-hand activities	e. FIM, mBI	c. 114	c. Random	c. 0.727 (0.051, 1.403)	c. 66	c. 0.898	c. NS
	c. self-reported amount of arm-hand use in daily life		d. 187	d. Fixed	d. 0.511 (0.224, 0.797)	d. 33	d. 0.856	d. NS
			e. 78	e. Random	e. 0.062 (-1.054, 1.178)	e. 83	e. 0.057	e. S
	d. self-reported quality of arm-hand movement in daily life							
	e. basic ADL							
– Low-intensity mCIMT	a. motor function arm (synergy)	a. FMA	a. 333	a. Fixed	a. 0.407 (0.190, 0.623)	a. 39	a. 0.887	a. S
		b. ARAT, WFMT	10	Fixed	2.211 (0.730, 3.692)	0	0.666	
	phase: ER	c. MAL	14	Random	-0.225 (-1.851, 1.402)	60	0.068	
	phase: LR	d. MAL	309	Fixed	0.394 (0.170, 0.618)	18	0.848	
	phase: C	e. BI, FIM	b. 337	b. Fixed	b. 0.597 (0.379, 0.815)	b. 41	b. 0.997	b. NS
	b. arm-hand activities	f. SIS	c. 441	c. Random	c. 0.764 (0.452, 1.075)	c. 57	c. 1.000	c. S
	c. self-reported amount of		10	Fixed	6.786 (3.608, 9.963)	0	0.947	

	arm-hand use in daily life		14	Fixed	1.455 (0.339, 2.570)	0	0.612	
	phase: ER		417	Fixed	0.651 (0.453, 0.849)	35	1.000	
	phase: LR		d. 442	d. Random	d. 0.647 (0.345, 0.949)	d. 55	d. 1.000	d. S
	phase: C		10	Fixed	4.251 (2.077, 6.424)	0	1.000	
	d. self-reported quality of arm-		14	Fixed	1.435 (0.322, 2.548)	0	0.602	
	hand movement in daily life		418	Fixed	0.550 (0.354, 0.746)	40	0.998	
	phase: ER		e. 233	e. Fixed	e. 0.449 (0.192, 0.706)	e. 0	e. 0.846	e. NS
	phase: LR		f. 36	f. Fixed	f. -0.261 (-0.891, 0.368)	f. 0	f. 0.104	f. NS
	phase: C							
	e. basic ADL							
	f. arm-hand related quality of							
	life							
Robotic-assisted arm								
training								
– Unilateral shoulder-	a. motor function arm	a. FMA, CMMSA, MSS	a. 327	a. Fixed	a. 0.195 (-0.013, 0.404)	a. 0	a. 0.343	a. NS
elbow robotics	(synergy)	b. FMA, AMAT	b. 283	b. Fixed	b. 0.339 (0.115, 0.563)	b. 0	b. 0.697	b. NS
	b. motor function arm proximal	c. FMA, AMAT, CMMSA, MSS	c. 224	c. Fixed	c. 0.063 (-0.188, 0.313)	c. 0	c. 0.069	c. NS
	(synergy)	d. MP, MRC, strength: elbow, shoulder	d. 278	d. Fixed	d. 0.475 (0.244, 0.706)	d. 42	d. 0.931	d. NS
	c. motor function arm distal	e. MP, MRC, strength: elbow, shoulder	e. 147	e. Fixed	e. 0.641 (0.312, 0.969)	e. 48	e. 0.913	e. NS
	(synergy)	f. GS, MRC	f. 56	f. Random	f. 0.289 (-0.544, 1.122)	f. 60	f. 0.154	f. NA
	d. muscle strength	g. MAS	g. 326	g. Random	g. -0.085 (-0.585, 0.415)	g. 78	g. 0.101	g. NS
	e. muscle strength proximal	h. Pain, FMA, CMMSA	h. 171	h. Fixed	h. 0.397 (0.091, 0.703)	h. 0	h. 0.604	h. NS
	f. muscle strength distal	i. ARAT, WMFT, BBT	i. 261	i. Fixed	i. 0.220 (-0.022, 0.461)	i. 0	i. 0.335	i. NS
	g. muscle tone	j. FIM	j. 133	j. Fixed	j. 0.037 (-0.279, 0.353)	j. 0	j. 0.054	j. NS
	h. pain	k. SIS	k. 198	k. Fixed	k. 0.180 (-0.098, 0.458)	k. 0	k. 0.199	k. NA
	i. arm-hand activities							
	j. basic ADL							
	k. quality of life							
– Bilateral elbow-wrist	a. motor function arm	a. FMA	a. 62	a. Fixed	a. 0.894 (0.408, 1.381)	a. 0	a. 0.841	a. NS
robotics	(synergy)	b. MRC	b. 62	b. Fixed	b. 1.072 (0.570, 1.574)	b. 49	b. 0.935	b. S
	b. muscle strength		44	Fixed	1.556 (0.891, 2.222)	0	0.975	
	phase: ER		18	Fixed	0.433 (-0.332, 1.197)	1	0.123	
	phase: C							
– Shoulder-elbow-wrist-	a. motor function arm	a. FMA, CMMSA	a. 36	a. Random	a. 0.067 (-1.397, 1.531)	a. 75	a. 0.053	a. NS
hand robotics	(synergy)	b. GS	b. 36	b. Fixed	b. 0.570 (-0.062, 1.202)	b. 0	b. 0.307	b. NS
	b. muscle strength distal							
Mental practice with	a. motor function arm	a. FMA	a. 149	a. Fixed	a. 0.174 (-0.118, 0.466)	a. 29	a. 0.154	a. NS
motor imagery	(synergy)	b. MI, GS, JTHFT, strength	b. 149	b. Random	b. 0.778 (-0.162, 1.719)	b. 83	b. 0.980	b. NA
	b. muscle strength	c. ARAT, AFT, JTHFT	c. 246	c. Random	c. 0.553 (0.110, 0.997)	c. 63	c. 0.954	c. NS
	c. arm-hand activities	d. BI, FIM	d. 151	d. Random	d. 0.074 (-0.244, 0.392)	d. 0	d. 0.068	d. NS
	d. basic ADL							
Mirror therapy for the	a. motor function arm	a. FMA	a. 112	a. Random	a. 0.391 (-0.146, 0.928)	a. 52	a. 0.434	a. NS
paretic arm	(synergy)	b. MAS, Tardieu	b. 76	b. Fixed	b. 0.410 (-0.035, 0.856)	b. 0	b. 0.335	b. NS
	b. muscle tone	c. VAS	c. 112	c. Random	c. 0.362 (-0.245, 0.969)	c. 52	c. 0.378	c. NS
	c. pain	d. ARAT, WMFT	d. 104	d. Random	d. 0.293 (-0.727, 1.312)	d. 82	d. 0.252	d. S
	d. arm-hand activities		48	Fixed	1.570 (0.931, 2.208)	0	0.994	
	phase: LR (n=1)		56	Fixed	-0.007 (-0.505, 0.492)	21	0.050	
	phase: C							
Virtual reality training for	a. motor function arm	a. FMA	a. 158	a. Fixed	a. 0.192 (-0.100, 0.485)	a. 0	a. 0.183	a. NS
the paretic arm	(synergy)	b. MAS, spasticity	b. 44	b. Fixed	b. -0.642 (-1.159, -0.124)	b. 0	b. 0.421	b. NA
	b. muscle tone	c. WMFT, BBT	c. 89	c. Fixed	c. 0.162 (-0.212, 0.535)	c. 0	c. 0.098	c. NS
	c. arm-hand activities	d. ABILHAND, CAHAI	d. 38	d. Random	d. 0.472 (-0.427, 1.370)	d. 50	d. 0.248	d. NS
	(unilateral)	e. BI, FIM	e. 63	e. Fixed	e. 0.801 (0.293, 1.309)	e. 48	e. 0.735	e. NS
	d. arm-hand activities							
	(bilateral)							
	e. basic ADL							
Electrostimulation of the								
paretic arm								
– NMS wrist/finger	a. motor function arm	a. FMA	a. 49	a. Random	a. 0.053 (-1.354, 1.461)	a. 84	a. 0.053	a. NS
extensors	(synergy)	b. ROM	b. 48	b. Random	b. 0.822 (-0.391, 2.036)	b. 76	b. 0.918	b. NS
	b. active range of motion	c. GS, PS, MI	c. 69	c. Random	c. -0.429 (-1.324, 0.465)	c. 71	c. 0.321	c. NA
	c. muscle strength	d. ARAT	d. 82	d. Random	d. 0.148 (-0.807, 1.103)	d. 79	d. 0.090	d. NS
	d. arm-hand activities							

– NMS wrist/finger flexors/extensors	a. motor function arm (synergy)<br>b. muscle strength<br>c. arm-hand activities	a. mFMA<br>b. JTHFT<br>c. BBT	a. 41<br>b. 23<br>c. 41	a. Fixed<br>b. Fixed<br>c. Fixed	a. 0.905 (0.285, 1.525)<br>b. 0.878 (0.046, 1.711)<br>c. 0.577 (-0.027, 1.181)	a. 0<br>b. 0<br>c. 13	a. 0.657<br>b. 0.429<br>c. 0.341	a. NA<br>b. NA<br>c. NA
– NMS shoulder	a. motor function arm (synergy)<br>b. range of motion<br>c. pain<br>d. subluxation	a. FMA<br>b. ROM<br>c. VAS<br>d. subluxation (x-ray)	a. 32<br>b. 58<br>c. 223<br>d. 190	a. Fixed<br>b. Fixed<br>c. Random<br>d. Random	a. 0.489 (-0.185, 1.163)<br>b. -0.152 (-0.649, 0.346)<br>c. 0.094 (-0.498, 0.687)<br>d. 0.579 (0.151, 1.008)	a. 33<br>b. 18<br>c. 68<br>d. 50	a. 0.219<br>b. 0.080<br>c. 0.094<br>d. 0.924	a. NS<br>b. NS<br>c. NS<br>d. NS
– EMG-NMS wrist/finger extensors	a. motor function arm (synergy)<br>b. active range of motion<br>c. muscle strength<br>d. muscle tone<br>e. arm-hand activities	a. FMA, CMMSA<br>b. ROM<br>c. GP, MI, strength, GS, JTHFT<br>d. MAS<br>e. ARAT, BBT, JTHFT, 10CMT, FTHUE	a. 49<br>b. 61<br>c. 79<br>d. 28<br>e. 162	a. Fixed<br>b. Random<br>c. Random<br>d. Fixed<br>e. Fixed	a. 0.578 (0.036, 1.119)<br>b. 1.156 (0.282, 2.030)<br>c. 0.508 (-0.192, 1.208)<br>d. 0.518 (-0.184, 1.221)<br>e. 0.718 (0.407, 1.028)	a. 0<br>b. 60<br>c. 60<br>d. 0<br>e. 49	a. 0.398<br>b. 0.960<br>c. 0.783<br>d. 0.215<br>e. 0.971	a. NS<br>b. NS<br>c. NS<br>d. NS<br>e. NS
– EMG-NMS wrist/finger flexors/extensors	a. motor function arm (synergy)<br>b. arm-hand activities	a. FMA, AMAT<br>b. WMFT, AMAT	a. 31<br>b. 31	a. Fixed<br>b. Fixed	a. 0.617 (-0.066, 1.301)<br>b. 0.578 (-0.108, 1.263)	a. 0<br>b. 22	a. 0.315<br>b. 0.284	a. NS<br>b. NS
– TENS	a. muscle tone<br>b. basic ADL	a. MAS<br>b. BI	a. 104<br>b. 88	a. Random<br>b. Random	a. 0.470 (-0.432, 1.372)<br>b. 0.972 (-0.450, 2.394)	a. 81<br>b. 89	a. 0.536<br>b. 0.962	a. NS<br>b. NS
Electromyographic biofeedback of the paretic arm	a. motor function arm (synergy)<br>b. active range of motion<br>c. arm-hand activities	a. FMA, Brunnstrom stages<br>b. ROM<br>c. UEFT	a. 69<br>b. 147<br>c. 102	a. Fixed<br>b. Fixed<br>c. Fixed	a. 0.390 (-0.075, 0.856)<br>b. 0.269 (-0.051, 0.589)<br>c. 0.211 (-0.166, 0.587)	a. 0<br>b. 0<br>c. 0	a. 0.282<br>b. 0.286<br>c. 0.149	a. NS<br>b. NS<br>c. NS
Trunk restraint	a. active range of motion<br>b. arm-hand activities<br>c. self-reported amount of arm-hand use in daily life	a. ROM<br>b. WMFT, BBT<br>c. MAL	a. 46<br>b. 58<br>c. 28	a. Fixed<br>b. Fixed<br>c. Fixed	a. 0.069 (-0.489, 0.626)<br>b. 0.066 (-0.427, 0.559)<br>c. -0.903 (-1.636, -0.169)	a. 0<br>b. 0<br>c. 0	a. 0.055<br>b. 0.056<br>c. 0.505	a. NA<br>b. NA<br>c. NA
Interventions for somatosensory functions of the paretic arm	a. motor function arm (synergy)<br>b. muscle strength<br>c. muscle tone<br>d. somatosensory function<br>e. pain<br>f. arm-hand activities<br>g. basic ADL	a. FMA, Brunnstrom stages<br>b. GS, PS, MI, grasping<br>c. MAS<br>d. NSA, FMA, sensory tests<br>e. VAS, FMA<br>f. ARAT, FMC<br>g. BI, FE	a. 170<br>b. 150<br>c. 65<br>d. 273<br>e. 41<br>f. 266<br>g. 168	a. Random<br>b. Fixed<br>c. Fixed<br>d. Fixed<br>e. Random<br>f. Fixed<br>g. Random	a. 0.456 (-0.028, 0.939)<br>b. 0.219 (-0.088, 0.526)<br>c. 0.548 (0.099, 0.997)<br>d. 0.358 (0.115, 0.660)<br>e. -0.367 (-1.431, 0.698)<br>f. 0.172 (-0.062, 0.405)<br>g. 0.118 (-0.426, 0.662)	a. 51<br>b. 0<br>c. 0<br>d. 47<br>e. 67<br>f. 0<br>g. 55	a. 0.716<br>b. 0.224<br>c. 0.474<br>d. 0.731<br>e. 0.170<br>f. 0.308<br>g. 0.102	a. NS<br>b. NS<br>c. NS<br>d. NS<br>e. NA<br>f. NS<br>g. NS

10CMT, 10-cup moving test; ADL, Activities of daily living; AFT, Arm function test; AMAT, Arm motor ability test; ARAT, Action research arm test; BBT, Box and block test; BI, Barthel index; C, Chronic phase; CAHAI, Chedoke arm and hand activity inventory; CI, Confidence interval; CMMSA, Chedoke-McMaster stroke assessment; ER, Early rehabilitation phase;FE, Functional evaluation; FIM, Functional independence measure; FMC, Fine motor control; FMA, Fugl-meyer assessment; FTHUE, Functional test for the hemiplegic upper extremity; GP, Grip power; GS, Grip strength; JTHFT, Jebsen-Taylor hand function test; LR, Late rehabilitation phase; MAL, Motor activity log; MAS, Modified ashworth scale; MAS*, Motor assessment scale; mFMA, modified Fugl-meyer assessment; MP, Motor power; MRC, Medical research council; MSS, Motor status scale; mBI, modified Barthel index; NA, Not applicable; NS, Not significant; NSA, Nottingham sensory assessment; PPT, Perdue pegboard test; PS, Pinch strength; ROM, Range of motion; S, Significant; SES, Summary effect size; SIS, Stroke impact scale; TEMPA, Test d'evaluation des membres supérieurs de personnes agéés; UEFT, Upper extremity function test; VAS, Visual analogue scale; WFMT, Wolf motor function test

TABLE S2C. Summary of the evidence for physical therapy interventions – physical fitness

Intervention	Pooling possible for:	Outcome measures	N (pooling)	Type effects model	SES (95%CI) Hedges' *g*	Heterogeneity I^2 (%)	Statistical power	Difference between poststroke phases
Strength training for the paretic leg	a. motor function leg (synergy)<br>b. muscle strength leg<br>c. muscle tone<br>d. comfortable gait speed<br>e. maximum gait speed<br>f. walking distance<br>g. spatiotemporal gait pattern parameters<br>h. aerobic capacity<br>i. peak heart rate<br>j. workload<br>k. physiological cost index<br>l. walking ability<br>m. basic ADL<br>n. quality of life	a. FMA<br>b. strength: ankle, hip, knee<br>c. MAS, TMS spasticity<br>d. 10MWT, WS<br>e. 10MWT<br>f. 6MWT, 12MWT<br>g. gait analysis<br>h. VO_2max<br>i. HR peak<br>j. peak power output<br>k. O_2-cost<br>l. CMMSA disability, FAP, mRMI, TUG, STS<br>m. BI, ADL<br>n. EQ, SF-36, LLFDM	a. 32<br>b. 328<br>c. 70<br>d. 390<br>e. 276<br>f. 350<br>g. 101<br>h. 48<br>i. 48<br>j. 48<br>k. 48<br>l. 373<br>m. 113<br>n. 158	a. Random<br>b. Fixed<br>c. Fixed<br>d. Fixed<br>e. Fixed<br>f. Fixed<br>g. Fixed<br>h. Fixed<br>i. Fixed<br>j. Fixed<br>k. Fixed<br>l. Fixed<br>m. Fixed<br>n. Fixed	a. 0.189 (-1.363, 1.740)<br>b. 0.350 (0.127, 0.573)<br>c. 0.894 (0.406, 1.381)<br>d. 0.087 (-0.117, 0.291)<br>e. 0.017 (-0.268, 0.302)<br>f. 0.064 (-0.148, 0.276)<br>g. 0.447 (0.050, 0.843)<br>h. -0.215 (-0.837, 0.407)<br>i. -0.523 (-1.152, 0.106)<br>j. -0.097 (-0.724, 0.531)<br>k. 0.382 (-0.241, 1.006)<br>l. 0.120 (-0.083, 0.322)<br>m. 0.369 (-0.039, 0.778)<br>n. 0.175 (-0.140, 0.489)	a. 74<br>b. 31<br>c. 42<br>d. 27<br>e. 0<br>f. 0<br>g. 0<br>h. 0<br>i. 0<br>j. 32<br>k. 0<br>l. 0<br>m. 36<br>n. 0	a. 0.075<br>b. 0.788<br>c. 0.880<br>d. 0.116<br>e. 0.051<br>f. 0.082<br>g. 0.489<br>h. 0.099<br>i. 0.473<br>j. 0.065<br>k. 0.286<br>l. 0.169<br>m. 0.395<br>n. 0.160	a. NA<br>b. NS<br>c. NS<br>d. NS<br>e. NS<br>f. NS<br>g. NS<br>h. NA<br>i. NA<br>j. NA<br>k. NA<br>l. NS<br>m. NS<br>n. NS
Strength training for the paretic arm	a. motor function arm (synergy)<br>b. muscle strength<br>c. pain<br>d. range of motion<br>e. arm-hand activities	a. FMA<br>b. GF, PF, strength: elbow, wrist<br>c. FMA<br>d. FMA<br>e. ARAT, NHPT, FTHUE	a. 96<br>b. 88<br>c. 39<br>d. 39<br>e. 88	a. Fixed<br>b. Fixed<br>c. Fixed<br>d. Fixed<br>e. Fixed	a. 0.176 (-0.224, 0.577)<br>b. 0.075 (-0.351, 0.500)<br>c. -0.181 (-0.707, 0.345)<br>d. 0.096 (-0.429, 0.621)<br>e. 0.196 (-0.248, 0.585)	a. 0<br>b. 0<br>c. 0<br>d. 0<br>e. 0	a. 0.116<br>b. 0.061<br>c. 0.093<br>d. 0.062<br>e. 0.103	a. NS<br>b. NA<br>c. NA<br>d. NA<br>e. NA
Cardiorespiratory exercises	a. motor function leg (synergy)<br>b. muscle strength leg<br>c. comfortable gait speed<br>d. maximum gait speed<br>e. walking distance<br>f. aerobic capacity<br>g. heart rate rest<br>*phase: ER*<br>*phase: C*<br>h. heart rate work<br>i. blood pressure rest diastolic<br>j. blood pressure rest systolic<br>k. workload<br>l. physiological cost index<br>m. respiratory functions<br>n. body composition<br>o. blood variables<br>p. balance<br>q. walking ability	a. FMA<br>b. (peak) power, strength leg<br>c. 10MWT, WS<br>d. 10MWT, WS<br>e. 2MWT, 6MWT, 12MWT, WD<br>f. VO_2max<br>g. HR rest<br>h. HR work<br>i. BP measurement<br>j. BP measurement<br>k. (peak) power output<br>l. O_2-cost<br>m. FEV1, VE, RER<br>n. BMI, body weight, body fat, waist girth, fat free mass<br>o. glucose, insulin, cholesterol<br>p. BBS, FSST<br>q. RMA GF, RMI, WQ, TUG	a. 80<br>b. 106<br>c. 321<br>d. 269<br>e. 339<br>f. 313<br>g. 206<br>118<br>88<br>h. 117<br>i. 116<br>j. 116<br>k. 192<br>l. 68<br>m. 88<br>n. 134<br>o. 92<br>p. 129<br>q. 228	a. Fixed<br>b. Fixed<br>c. Fixed<br>d. Fixed<br>e. Fixed<br>f. Fixed<br>g. Fixed<br>Fixed<br>Fixed<br>h. Random<br>i. Fixed<br>j. Fixed<br>k. Fixed<br>l. Fixed<br>m. Random<br>n. Fixed<br>o. Fixed<br>p. Fixed<br>q. Fixed	a. -0.175 (-0.607, 0.256)<br>b. 0.246 (-0.152, 0.644)<br>c. 0.210 (-0.009, 0.430)<br>d. 0.145 (-0.094, 0.384)<br>e. 0.210 (0.000, 0.420)<br>f. 0.353 (0.130, 0.577)<br>g. 0.154 (-0.117, 0.425)<br>0.416 (0.056, 0.776)<br>-0.189 (-0.601, 0.223)<br>h. 0.026 (-0.711, 0.763)<br>i. -0.031 (-0.388, 0.327)<br>j. -0.184 (-0.542, 0.173)<br>k. 0.638 (0.342, 0.934)<br>l. 0.072 (-0.428, 0.572)<br>m. 0.578 (0.157, 0.999)<br>n. -0.262 (0.599, 0.075)<br>o. 0.395 (-0.014, 0.803)<br>p. 0.118 (-0.220, 0.456)<br>q. 0.140 (-0.115, 0.395)	a. 0<br>b. 43<br>c. 0<br>d. 0<br>e. 0<br>f. 0<br>g. 41<br>0<br>0<br>h. 71<br>i. 0<br>j. 0<br>k. 44<br>l. 0<br>m. 0<br>n. 0<br>o. 0<br>p. 19<br>q. 0	a. 0.104<br>b. 0.200<br>c. 0.388<br>d. 0.178<br>e. 0.384<br>f. 0.775<br>g. 0.161<br>0.498<br>0.120<br>h. 0.054<br>i. 0.052<br>j. 0.141<br>k. 0.962<br>l. 0.058<br>m. 0.903<br>n. 0.255<br>o. 0.370<br>p. 0.090<br>q. 0.150	a. NA<br>b. NA<br>c. NS<br>d. NS<br>e. NS<br>f. NA<br>g. S<br><br><br>h. NS<br>i. NS<br>j. NS<br>k. NS<br>l. NA<br>m. NA<br>n. NA<br>o. NA<br>p. NA<br>q. NS
Mixed strength and cardiorespiratory exercises	a. motor function leg (synergy)<br>b. motor function arm (synergy)<br>c. muscle strength leg<br>d. muscle strength arm<br>e. comfortable gait speed<br>f. maximum gait speed<br>g. walking distance<br>h. aerobic capacity<br>i. heart rate work<br>j. physiological cost index<br>k. depression<br>l. balance<br>m. walking ability<br>n. arm-hand activities<br>o. basic ADL<br>p. extended ADL<br>q. physical activity<br>r. quality of life	a. FMA<br>b. FMA<br>c. strength: ankle, knee, leg<br>d. GS, JTHFT objects<br>e. 10WMT, 5MWT, WS<br>f. 10MWT, 5MWT<br>g. 6MWT<br>h. VO_2max<br>i. HR work<br>j. O_2-cost<br>k. HADS, GDS, SCL-90<br>l. BBS, FMA balance, FR<br>m. BI ambulation, RMI, TUG, STS<br>n. JTHFT, PPT<br>o. BI, FIM, Katz scale<br>p. Lawton IADL, NEADL<br>q. PADS, PASIPD<br>r. SF-36	a. 136<br>b. 136<br>c. 313<br>d. 156<br>e. 344<br>f. 182<br>g. 466<br>h. 256<br>i. 48<br>j. 114<br>k. 194<br>l. 399<br>m. 190<br>n. 29<br>o. 197<br>p. 179<br>q. 98<br>r. 307	a. Fixed<br>b. Fixed<br>c. Fixed<br>d. Fixed<br>e. Fixed<br>f. Fixed<br>g. Fixed<br>h. Fixed<br>i. Fixed<br>j. Fixed<br>k. Random<br>l. Fixed<br>m. Fixed<br>n. Fixed<br>o. Fixed<br>p. Fixed<br>q. Fixed<br>r. Fixed	a. 0.300 (-0.031, 0.631)<br>b. 0.451 (0.116, 0.785)<br>c. 0.369 (0.144, 0.595)<br>d. 0.166 (-0.155, 0.488)<br>e. 0.313 (0.101, 0.525)<br>f. 0.361 (0.064, 0.659)<br>g. 0.215 (0.009, 0.421)<br>h. 0.449 (0.199, 0.700)<br>i. 0.802 (0.162, 1.442)<br>j. 0.214 (-0.168, 0.596)<br>k. 0.361 (-0.259, 0.981)<br>l. 0.275 (0.080, 0.470)<br>m. 0.202 (-0.077, 0.482)<br>n. 0.335 (-0.518, 1.187)<br>o. 0.095 (-0.183, 0.372)<br>p. 0.169 (-0.122, 0.460)<br>q. 0.434 (0.039, 0.830)<br>r. 0.438 (0.210, 0.666)	a. 0<br>b. 0<br>c. 29<br>d. 0<br>e. 0<br>f. 0<br>g. 0<br>h. 20<br>i. 0<br>j. 13<br>k. 73<br>l. 0<br>m. 0<br>n. 0<br>o. 25<br>p. 15<br>q. 0<br>r. 0	a. 0.326<br>b. 0.615<br>c. 0.810<br>d. 0.149<br>e. 0.701<br>f. 0.549<br>g. 0.520<br>h. 0.863<br>i. 0.640<br>j. 0.165<br>k. 0.576<br>l. 0.660<br>m. 0.226<br>n. 0.119<br>o. 0.090<br>p. 0.166<br>q. 0.460<br>r. 0.911	a. NA<br>b. NA<br>c. NS<br>d. NS<br>e. NS<br>f. NS<br>g. NS<br>h. NS<br>i. NA<br>j. NS<br>k. NS<br>l. NS<br>m. NS<br>n. NS<br>o. NS<br>p. NS<br>q. NS<br>r. NS

10MWT, 10-meter walk test; 12MWT, 12-minute walk test; 5MWT, 5-meter walk test; 6MWT, 6-minute walk test; ADL, Activities of daily living; ARAT, Action research arm test; BBS, Berg balance scale; BI, Barthel index; BMI, Body mass index; BP, Blood pressure; C, Chronic phase; CI, Confidence interval; CMMSA, Chedoke-McMaster stroke assessment; EQ, EuroQoL 5D; ER, Early rehabilitation phase; FAP, Functional ambulation profile; FEV1, Forced expiratory volume in 1 second; FIM, Functional independence measure; FMA, Fugl-meyer assessment; FR, Functional reach; FSST, Four square step test; FTHUE, Functional test for the hemiplegic upper extremity; GF, Grip force; GS, Grip strength; HADS, Hospital anxiety and depression scale; HR, Heart rate; IADL, Instrumental ADL; JTHFT, Jebsen-Taylor hand function test; LLFDI, Late life function and disability instrument; LR, Late rehabilitation phase; MAS, Modified ashworth scale; NA, Not applicable; NEADL, Nottingham extended ADL index; NHPT, Nine hole peg test; NS, Not significant; O_2cost, Oxygen cost; PADS, physical activities and disability scale; PASIPD, Physical activity scale for individuals with physical disabilities; PF, Pinch force; PPT, Perdue pegboard test; RER, Respiratory exchange ratio; RMA GF, Rivermead motor assessment gross function; RMI, Rivermead mobility index; S, Significant; SES, Summary effect size; SF-36, 36-item Short form health survey; SLC90, Symptom checklist-90-R; STS, Sit-to-stand; TMS, Tolouse motor scale; TUG, Timed up and go test; VE, Ventilatory exchange; VO_2max, Ventilatory oxygen uptake, WD, Walking distance; WS, WQ, Walking questionnaire; Walking speed gait analysis

TABLE S2D. Summary of the evidence for physical therapy interventions – activities of daily living

Intervention	Pooling possible for:	Outcome measures	N (pooling)	Type effects model	SES (95%CI) Hedges' *g*	Heterogeneity I^2 (%)	Statistical power	Difference between poststroke phases
Interventions for apraxia – gestural training	a. gesture comprehension	a. gesture comprehension test	a. 46	a. Fixed	a. 0.631 (0.057, 1.205)	a. 0	a. 0.456	a. NA
	b. ideational apraxia	b. ideational apraxia test	b. 46	b. Fixed	b. 0.018 (-0.545, 0.580)	b. 24	b. 0.050	b. NA
	c. ideomotor apraxia	c. de Renzi test	c. 46	c. Fixed	c. 0.454 (-0.112, 1.020)	c. 0	c. 0.270	c. NA
Leisure therapy	a. leisure participation	a. NLQ, TLAS, TLS, participation in leisure	a. 528	a. Random	a. 0.459 (0.068, 0.851)	a. 71	a. 0.995	a. S
	*phase: ER (n=1)**	b. CES-D, BDI, WDI	62	Fixed	1.236 (0.679, 1.794)	0	b. 0.975	b. NS
	*phase: LR (n=1)**	c. GHQ, GWBS	410	Fixed	0.106 (-0.103, 0.314)	0	0.154	c. NS
	phase: C (n=1)	d. SIP, SA-SIP30	56	Fixed	0.323 (-0.209, 0.855)	0	0.180	d. NS
	b. depression		b. 131	b. Fixed	b. 0.346 (-0.020, 0.713)	b. 47	c. 0.392	
	c. mood		c. 466	c. Fixed	c. 0.151 (-0.043, 0.345)	c. 0	d. 1.000	
	d. quality of life		d. 69	d. Random	d. 1.802 (-0.267, 3.870)	d. 85		

*1 RCT with 2 comparisons; NLQ, Nottingham leisure questionnaire; BDI, Beck depression inventory; C, Chronic phase; CES-D, Centre for epidemiologic studies for depression scale; CI, Confidence interval; ER, Early rehabilitation phase; GHQ, General health questionnaire; GWBS, General well-being scale; LR, Late rehabilitation phase; NA, Not applicable; NS, Not significant; TLAS, Total leisure activities score; TLS, Total leisure score; S, Significant; SES, Summary effect size; SIP, Stroke impact profile; SA-SIP30, Stroke-adapted 30-item version of the sickness impact profile; WDI, Wakefield depression inventory

TABLE S2E. Summary of the evidence for physical therapy interventions – other

Intervention	Pooling possible for:	Outcome measures	N (pooling)	Type effects model	SES (95%CI) Hedges' *g*	Heterogeneity I^2 (%)	Statistical power	Difference between poststroke phases
Inspiratory muscle training	a. maximal inspiratory pressure<br>*phase: LR*<br>*phase: C (n=1)*	a. MIP	a. 63<br>45<br>18	a. Random<br>Fixed<br>Fixed	a. 0.842 (-0.365, 1.367)<br>0.221 (-0.380, 0.821)<br>2.235 (1.092, 3.379)	a. 80<br>0<br>0	a. 0.799<br>0.097<br>0.897	a. S

CI, Confidence interval; C, Chronic phase; LR, Late rehabilitation phase; MIP, Maximal inspiratory pressure; S, Significant; SES, Summary effect size

TABLE S2F. Summary of the evidence for physical therapy interventions – intensity of practice

Intervention	Pooling possible for:	Outcome measures	N (pooling)	Type effects model	SES (95%CI) Hedges' *g*	Heterogeneity I^2 (%)	Statistical power	Difference between poststroke phases
High-intensity practice	a. motor function leg (synergy)	e. FMA	a. 324	a. Fixed	a. 0.400 (0.180, 0.620)	a. 49	a. 0.869	a. S
	phase: ER	f. FMA	284	Fixed	0.273 (0.040, 0.506)	0	0.505	b. NA
	phase: LR (n=1)	g. strength: hip, knee	33	Fixed	1.559 (0.180, 0.620)	0	0.932	c. NS
	phase: C (n=1)	h. GS, PS, MI, grasping, strength: arm, elbow	7	Fixed	1.095 (-0.290, 2.480)	0	0.240	d. NS
	b. motor function arm	i. 10MWT, 5MWT, WS	b. 454	b. Fixed	b. 0.207 (0.022, 0.393)	b. 6	b. 0.476	e. NS
	(synergy)	j. 10MWT, 5MWT	c. 404	c. Fixed	c. 0.613 (0.411, 0.815)	c. 41	c. 0.999	f. NS
	c. muscle strength leg	k. 6MWT, WD	d. 997	d. Fixed	d. 0.038 (-0.087, 0.163)	d. 5	d. 0.082	g. S
	d. muscle strength arm	l. MAS	e. 1097	e. Fixed	e. 0.288 (0.169, 0.407)	e. 45	e. 0.992	h. S
	e. comfortable gait speed	m. BDI, GDS-15, HADS, SCL-90	f. 283	f. Fixed	f. 0.366 (0.132, 0.600)	f. 0	f. 0.761	i. NS
	f. maximum gait speed	n. GHQ	g. 512	g. Fixed	g. 0.086 (-0.086, 0.259)	g. 44	g. 0.134	j. NA
	g. walking distance	o. ABC, FES-I	278	Fixed	0.067 (-0.166, 0.299)	0	0.077	k. NS
	phase: ER	p. BBS, PASS, FR, ST, FSST	159	Fixed	0.422 (0.133, 0.730)	0	0.629	l. NS
	phase: C	q. FAC, RMI, mRMI, STREAM, RMA, POR, BI	75	Fixed	-0.576 (-0.120, 0.236)	0	0.569	m. NS
	Phase: all (n=1)	r. ARAT, NHPT, FTHUE, RMA	h. 277	h. Fixed	h. 0.294 (0.056, 0.531)	h. 45	h. 0.564	n. NS
	h. muscle tone	s. BI, mBI, FIM, ADL-index, RMA	227	Fixed	0.137 (-0.123, 0.397)	0	0.167	o. NS
	phase: ER	t. NEADL, FAI, Lawton IADL, RMA, LHS	50	Fixed	1.094 (0.508, 1.680)	0	0.879	p. NS
	phase: C (n=1)	u. falls	i. 732	i. Random	i. 0.376 (0.133, 0.619)	i. 52	i. 0.992	q. NS
	i. depression and anxiety	v. SIS, SIP, MOS-36, EuroQoL, LSQ, SF-36, COOP scale, NHP	j. 555	j. Fixed	j. 0.117 (-0.056, 0.291)	j. 0	j. 0.224	r. NS
	j. mental health patient		k. 59	k. Fixed	k. 0.241 (-0.262, 0.743)	k. 0	k. 0.127	s. NS
	k. falls-efficacy	w. GHQ	l. 957	l. Random	l. 0.257 (0.001, 0.513)	l. 71	l. 0.930	
	l. balance		m. 1130	m. Random	m. 0.170 (-0.048, 0.388)	m. 66	m. 0.704	
	m. walking ability		n. 1122	n. Fixed	n. 0.097 (-0.021, 0.215)	n. 0	n. 0.286	
	n. arm-hand activities		o. 3064	o. Random	o. 0.217 (0.094, 0.339)	o. 62	o. 0.999	
	o. basic ADL		p. 1821	p. Random	p. 0.071 (-0.073, 0.216)	p. 55	p. 0.260	
	p. extended ADL		q. 209	q. Fixed	q. 0.112 (-0.323, 0.547)	q. 0	q. 0.108	
	q. falls		r. 780	r. Fixed	r. 0.413 (0.269, 0.556)	r. 30	r. 0.998	
	r. quality of life		s. 537	s. Fixed	s. 0.082 (-0.100, 0.263)	s. 0	s. 0.131	
	s. mental health caregiver							

10MWT, 10-meter walk test; 5MWT, 5-meter walk test; ABC, Activities-specific balance confidence scale; ADL, Activities of daily living; ARAT, Action research arm test; BBS, Berg balance scale; BDI, Beck depression inventory; BI, Barthel index; C, Chronic phase; CI, Confidence interval; COOP scale, Dartmouth primary care cooperative information functional health assessment; ER, Early rehabilitation phase; FAC, Functional ambulation categories; FAI, Frenchay activities index; FES-I, Falls-efficacy scale; FIM, Functional independence measure; FMA, Fugl-meyer assessment; FR, Functional reach; FSST, Four square step test; FTHUE, Functional test for the hemiplegic upper extremity; GDS, Geriatric depression scale – 15; GHQ, General health questionnaire; GS, Grip strength; HADS, Hospital anxiety and depression scale; IADL, Instrumental ADL; LHS, London handicap scale; LR, Late rehabilitation phase; MAS, Modified ashworth scale; mBI, modified Barthel index; MI, Motricity index; mRMI, modified Rivermead mobility index; NA, Not applicable; NEADL, Nottingham extended ADL index; NHP, Nottingham health profile; NHPT, Nine hole peg test; NS, Not significant; PASS, Postural assessment scale for stroke; POR, profile of recovery; PS, Pinch strength; RMA, Rivermead motor assessment; RMI, Rivermead mobility index; S, Significant; SCL-90, Symptom checklist-90-R; SES, Summary effect size; SF-36, 36-item Short form health survey; SIP, Stroke impact scale, SIS, Stroke impact scale; ST, Step test; STREAM, Stroke rehabilitation assessment of movement instrument; WD, Walking distance; WS, Walking speed gait analysis

REFERENCES SUPPORTING INFORMATION TABLES

1. Bernhardt J, Dewey H, Thrift A, Collier J, Donnan G (2008) A very early rehabilitation trial for stroke (AVERT): phase II safety and feasibility. Stroke 39: 390-396.
2. Langhorne P, Stott D, Knight A, Bernhardt J, Barer D, et al. (2010) Very early rehabilitation or intensive telemetry after stroke: a pilot randomised trial. Cerebrovasc Dis 29: 352-360.
3. Dean C, Shepherd R (1997) Task-related training improves performance of seated reaching tasks after stroke. A randomized controlled trial. Stroke 28: 722-728.
4. De Sèze M, Wiart L, Bon-Saint-Côme A, Debelleix X, de Sèze M, et al. (2001) Rehabilitation of postural disturbances of hemiplegic patients by using trunk control retraining during exploratory exercises. Arch Phys Med Rehabil 82: 793-800.
5. Mudie M, Winzeler-Mercay U, Radwan S, Lee L (2002) Training symmetry of weight distribution after stroke: a randomized controlled pilot study comparing task-related reach, Bobath and feedback training approaches. Clin Rehabil 16: 582-592.
6. Pollock A, Durward B, Rowe P, Paul J (2002) The effect of independent practice of motor tasks by stroke patients: a pilot randomized controlled trial. Clin Rehabil 16: 473-480.
7. Dean C, Channon E, Hall J (2007) Sitting training early after stroke improves sitting ability and quality and carries over to standing up but not to walking: a randomised trial. Aust J Physiother 53: 97-102.
8. Ibrahimi N, Tufel S, Singh H, Maurya M (2010) Effect of sitting balance training under varied sensory input on balance and quality of life in stroke patients. Ind J Physiother Occup Ther 4: 40-45.
9. Engardt M, Ribbe T, Olsson E (1993) Vertical ground reaction force feedback to enhance stroke patients' symmetrical body-weight distribution while rising/sitting down. Scand J Rehabil Med 25: 41-48.
10. Barreca S, Sigouin C, Lambert C, Ansley B (2004) Effects of extra training on the ability of stroke survivors tp perform an independent ist-to-stand: a randomized controlled trial. J Geriatr Phys Ther 27: 59-68.
11. Britton E, Harris N, Turton A (2008) An exploratory randomized controlled trial of assisted practice for improving sit-to-stand in stroke patients in the hospital setting. Clin Rehabil 22: 458-468.
12. Tung F, Yang Y, Lee C, Wang R (2010) Balance outcomes after additional sit-to-stand training in subjects with stroke: a randomized controlled trial. Clin Rehabil 24: 533-542.
13. Varoqui D, Froger J, Pelissier J, Bardy B (2011) Effect of coordination biofeedback on (re)learning preferred postural patterns in post-stroke patients. Motor Control 15: 187-205.
14. Morioka S, Yagi F (2003) Effects of perceptual learning exercises on standing balance using a hardness discrimination task in hemiplegic patients following stroke: a randomized controlled pilot trial. Clin Rehabil 17: 600-607.
15. Bagley P, Hudson M, Forster A, Smith J, Young J (2005) A randomized trial evaluation of the Oswestry Standing Frame for patients after stroke. Clin Rehabil 19: 354-364.
16. Bayouk J, Boucher J, Leroux A (2006) Balance training following stroke: effects of task-oriented exercises with and without altered sensory input. Int J Rehabil Res 29: 51-59.
17. Allison R, Dennett R (2007) Pilot randomized controlled trial to assess the impact of additional supported standing practice on functional ability post stroke. Clin Rehabil 21: 614-619.
18. Shumway-Cook A, Anson D, Haller S (1988) Postural sway biofeedback: its effect on reestablishing stance stability in hemiplegic patients. Arch Phys Med Rehabil 69: 395-400.
19. Grant T, Brouwer B, Culham E (1997) Balance retraining following acute stroke: a comparison of two methods. Can J Rehabil 11: 69-73.
20. Sackley C, Lincoln N (1997) Single blind randomized controlled trial of visual feedback after stroke: effects on stance symmetry and function. Disabil Rehabil 19: 536-546.
21. Walker C, Brouwer B, Culham E (2000) Use of visual feedback in retraining balance following acute stroke. Phys Ther 80: 886-895.
22. Geiger R, Allen J, O'Keefe J, Hicks R (2001) Balance and mobility following stroke: effects of physical therapy interventions with and without biofeedback/forceplate training. Phys Ther 81: 995-1005.
23. Chen I, Cheng P, Chen C, Chen S, Chung C, et al. (2002) Effects of balance training on hemiplegic stroke patients. Chang Gung Med J 25: 583-590.
24. Kerdoncuff V, Durufle A, Petrilli S, Nicolas B, Robineau S, et al. (2004) [Interest of visual biofeedback training in rehabilitation of balance after stroke]. Ann Readapt Med Phys 47: 169-176.
25. Heller F, Beuret-Blanquart F, Weber J (2005) [Postural biofeedback and locomotion reeducation in stroke patients]. Ann Readapt Med Phys 48: 187-195.

26. Yavuzer G, Eser F, Karakus D, Karaoglan B, Stam H (2006) The effects of balance training on gait late after stroke: a randomized controlled trial. Clin Rehabil 20: 960-969.
27. Eser F, Yavuzer G, Karakus D, Karaoglan B (2008) The effect of balance training on motor recovery and ambulation after stroke: a randomized controlled trial. Eur J Phys Rehabil Med 44: 19-25.
28. Gok H, Alptekin N, Geler-Kulcu D, Dincer G (2008) Efficacy of treatment with a kinaesthetic ability training device on balance and mobility after stroke: a randomized controlled study. Clin Rehabil 22: 922-930.
29. Goljar N, Burger H, Rudolf M, Stanonik I (2010) Improving balance in subacute stroke patients: a randomized controlled study. Int J Rehabil Res 33: 205-210.
30. Cheng P, Wu S, Liaw M, Wong A, Tang F (2001) Symmetrical body-weight distribution training in stroke patients and its effect on fall prevention. Arch Phys Med Rehabil 82: 1650-1654.
31. Bonan I, Yelnik A, Colle F, Michaud C, Normand E, et al. (2004) Reliance on visual information after stroke. Part II: Effectiveness of a balance rehabilitation program with visual cue deprivation after stroke: a randomized controlled trial. Arch Phys Med Rehabil 85: 274-278.
32. McClellan R, Ada L (2004) A six-week, resource-efficient mobility program after discharge from rehabilitation improves standing in people affected by stroke: placebo-controlled, randomised trial. Aust J Physiother 50: 163-167.
33. Howe T, Taylor I, Finn P, Jones H (2005) Lateral weight transference exercises following acute stroke: a preliminary study of clinical effectiveness. Clin Rehabil 19: 45-53.
34. Marigold D, Eng J, Dawson A, Inglis J, Harris J, et al. (2005) Exercise leads to faster postural reflexes, improved balance and mobility, and fewer falls in older persons with chronic stroke. J Am Geriatr Soc 53: 416-423.
35. Yelnik A, le Breton F, Colle F, Bonan I, Hugeron C, et al. (2008) Rehabilitation of balance after stroke with multisensorial training: a single-blind randomized controlled study. Neurorehabil Neural Repair 22: 468-476.
36. Verheyden G, Vereeck L, Truijen S, Troch M, Lafosse C, et al. (2009) Additional exercises improve trunk performance after stroke: a pilot randomized controlled trial. Neurorehabil Neural Repair 23: 281-286.
37. Askim T, Morkved S, Engen A, Roos K, Aas T, et al. (2010) Effects of a community-based intensive motor training program combined with early supported discharge after treatment in a comprehensive stroke unit: a randomized, controlled trial. Stroke 41: 1697-1703.
38. Holmgren E, Gosman-Hedström G, Lindström B, Wester P (2010) What is the benefit of a high-intensive exercise program on health-related quality of life and depression after stroke? A randomized controlled trial. Adv Physiother 12: 115-124.
39. Karthikbabu S, Nayak A, Vijayakumar K, Misri Z, Suresh B, et al. (2011) Comparison of physio ball and plinth trunk exercises regimens on trunk control and functional balance in patients with acute stroke: a pilot randomized controlled trial. Clin Rehabil 25: 709-719.
40. Merkert J, Butz S, Nieczaj R, Steinhagen-Thiessen E, Eckardt R (2011) Combined whole body vibration and balance training using Vibrosphere(R) : Improvement of trunk stability, muscle tone, and postural control in stroke patients during early geriatric rehabilitation. Z Gerontol Geriatr.
41. Visintin M, Barbeau H, Korner-Bitensky N, Mayo N (1998) A new approach to retrain gait in stroke patients through body weight support and treadmill stimulation. Stroke 29: 1122-1128.
42. Barbeau H, Visintin M (2003) Optimal outcomes obtained with body-weight support combined with treadmill training in stroke subjects. Arch Phys Med Rehabil 84: 1458-1465.
43. Kosak M, Reding M (2000) Comparison of partial body weight-supported treadmill gait training versus aggressive bracing assisted walking post stroke. Neurorehabil Neural Repair 14: 13-19.
44. Teixeira da Cunha Filho I, Lim P, Qureshy H, Henson H, Monga T, et al. (2001) A comparison of regular rehabilitation and regular rehabilitation with supported treadmill ambulation training for acute stroke patients. J Rehabil Res Dev 38: 245-255.
45. Da Cunha Jr I, Lim P, Qureshy H, Henson H, Monga T, et al. (2002) Gait outcomes after acute stroke rehabilitation with supported treadmill ambulation training: a randomized controlled pilot study. Arch Phys Med Rehabil 83: 1258-1265.
46. Nilsson L, Carlsson J, Danielsson A, Fugl-Meyer A, Hellström K, et al. (2001) Walking training of patients with hemiparesis at an early stage after stroke: a comparison of walking training on a treadmill with body weight support and walking training on the ground. Clin Rehabil 15: 515-527.

47. Sullivan K, Knowlton B, Dobkin B (2002) Step training with body weight support: effect of treadmill speed and practice paradigms on poststroke locomotor recovery. Arch Phys Med Rehabil 83: 683-691.
48. Werner C, von Frankenberg S, Treig T, Konrad M, Hesse S (2002) Treadmill training with partial body weight support and an electromechanical gait trainer for restoration of gait in subacute stroke patients: a randomized crossover study. Stroke 33: 2895-2901.
49. Eich H, Mach H, Werner C, Hesse S (2004) Aerobic treadmill plus Bobath walking training improves walking in subacute stroke: a randomized controlled trial. Clin Rehabil 18: 640-651.
50. Suputtitada A, Yooktanan P, Rarerng-Ying T (2004) Effect of partial body weight support treadmill training in chronic stroke patients. J Med Assoc Thai 87 Suppl 2: S107-S111.
51. Yagura H, Hatakenaka M, Miyai I (2006) Does therapeutic facilitation add to locomotor outcome of body weight--supported treadmill training in nonambulatory patients with stroke? A randomized controlled trial. Arch Phys Med Rehabil 87: 529-535.
52. Sullivan K, Brown D, Klassen T, Mulroy S, Ge T, et al. (2007) Effects of task-specific locomotor and strength training in adults who were ambulatory after stroke: results of the STEPS randomized clinical trial. Phys Ther 87: 1580-1602.
53. Yen C, Wang R, Liao K, Huang C, Yang Y (2008) Gait training induced change in corticomotor excitability in patients with chronic stroke. Neurorehabil Neural Repair 22: 22-30.
54. Franceschini M, Carda S, Agosti M, Antenucci R, Malgrati D, et al. (2009) Walking after stroke: what does treadmill training with body weight support add to overground gait training in patients early after stroke?: a single-blind, randomized, controlled trial. Stroke 40: 3079-3085.
55. Westlake K, Patten C (2009) Pilot study of Lokomat versus manual-assisted treadmill training for locomotor recovery post-stroke. J Neuroeng Rehabil 6: 18.
56. Dean C, Ada L, Bampton J, Morris M, Katrak P, et al. (2010) Treadmill walking with body weight support in subacute non-ambulatory stroke improves walking capacity more than overground walking: a randomised trial. J Physiother 56: 97-103.
57. Ada L, Dean C, Morris M, Simpson J, Katrak P (2010) Randomized trial of treadmill walking with body weight support to establish walking in subacute stroke: the MOBILISE trial. Stroke 41: 1237-1242.
58. Moore J, Roth E, Killian C, Hornby T (2010) Locomotor training improves daily stepping activity and gait efficiency in individuals poststroke who have reached a "plateau" in recovery. Stroke 41: 129-135.
59. Takami A, Wakayama S (2010) Effects of partial body weight support while training acute stroke patients to walk backwards on a treadmill - a controlled clinical trial using randomized allocation. J Phys Ther Sci 22: 177-187.
60. Yang Y, Chen I, Liao K, Huang C, Wang R (2010) Cortical reorganization induced by body weight-supported treadmill training in patients with hemiparesis of different stroke durations. Arch Phys Med Rehabil 91: 513-518.
61. Duncan P, Sullivan K, Behrman A, zen S, u S, et al. (2011) Body-weight-supported treadmill rehabilitation after stroke. N Engl J Med 364: 2026-2036.
62. Peurala S, Tarkka I, Pitkänen K, Sivenius J (2005) The effectiveness of body weight-supported gait training and floor walking in patients with chronic stroke. Arch Phys Med Rehabil 86: 1557-1564.
63. Tong R, Ng M, Li L (2006) Effectiveness of gait training using an electromechanical gait trainer, with and without functional electric stimulation, in subacute stroke: a randomized controlled trial. Arch Phys Med Rehabil 87: 1298-1304.
64. Dias D, Lains J, Pereira A, Nunes R, Caldas J, et al. (2007) Can we improve gait skills in chronic hemiplegics? A randomised control trial with gait trainer. Eura Medicophys 43: 499-504.
65. Husemann B, Muller F, Krewer C, Heller S, Koenig E (2007) Effects of locomotion training with assistance of a robot-driven gait orthosis in hemiparetic patients after stroke: a randomized controlled pilot study. Stroke 38: 349-354.
66. Mayr A, Kofler M, Quirbach E, Matzak H, Frohlich K, et al. (2007) Prospective, blinded, randomized crossover study of gait rehabilitation in stroke patients using the Lokomat gait orthosis. Neurorehabil Neural Repair 21: 307-314.
67. Pohl M, Werner C, Holzgraefe M, Kroczek G, Mehrholz J, et al. (2007) Repetitive locomotor training and physiotherapy improve walking and basic activities of daily living after stroke: a single-blind, randomized multicentre trial (DEutsche GAngtrainerStudie, DEGAS). Clin Rehabil 21: 17-27.

68. Mehrholz J, Werner C, Hesse S, Pohl M (2008) Immediate and long-term functional impact of repetitive locomotor training as an adjunct to conventional physiotherapy for non-ambulatory patients after stroke. Disabil Rehabil 30: 830-836.
69. Hornby T, Campbell D, Kahn J, Demott T, Moore J, et al. (2008) Enhanced gait-related improvements after therapist- versus robotic-assisted locomotor training in subjects with chronic stroke: a randomized controlled study. Stroke 39: 1786-1792.
70. Lewek M, Cruz T, Moore J, Roth H, Dhaher Y, et al. (2009) Allowing intralimb kinematic variability during locomotor training poststroke improves kinematic consistency: a subgroup analysis from a randomized clinical trial. Phys Ther 89: 829-839.
71. Ng M, Tong R, Li L (2008) A pilot study of randomized clinical controlled trial of gait training in subacute stroke patients with partial body-weight support electromechanical gait trainer and functional electrical stimulation: six-month follow-up. Stroke 39: 154-160.
72. Hidler J, Nichols D, Pelliccio M, Brady K, Campbell D, et al. (2009) Multicenter randomized clinical trial evaluating the effectiveness of the Lokomat in subacute stroke. Neurorehabil Neural Repair 23: 5-13.
73. Peurala S, Airaksinen O, Huuskonen P, Jakala P, Juhakoski M, et al. (2009) Effects of intensive therapy using gait trainer or floor walking exercises early after stroke. J Rehabil Med 41: 166-173.
74. Schwartz I, Sajin A, Fisher I, Neeb M, Shochina M, et al. (2009) The effectiveness of locomotor therapy using robotic-assisted gait training in subacute stroke patients: a randomized controlled trial. PM R 1: 516-523.
75. Fisher S, Lucas L, Thrasher T (2011) Robot-assisted gait training for patients with hemiparesis due to stroke. Top Stroke Rehabil 18: 269-276.
76. Morone G, Bragoni M, Iosa M, de Angelis D, Venturiero V, et al. (2011) Who may benefit from robotic-assisted gait training? A randomized clinical trial in patients with subacute stroke. Neurorehabil Neural Repair.
77. Chang W, Kim M, Huh J, Lee P, Kim Y (2012) Effects of robot-assisted gait training on cardiopulmonary fitness in subacute stroke patients: a randomized controlled study. Neurorehabil Neural Repair 26: 318-324.
78. Liston R, Mickelborough J, Harris B, Hann A, Tallis R (2000) Conventional physiotherapy and treadmill re-training for higher-level gait disorders in cerebrovascular disease. Age Ageing 29: 311-318.
79. Laufer Y, Dickstein R, Chefez Y, Marcovitz E (2001) The effect of treadmill training on the ambulation of stroke survivors in the early stages of rehabilitation: a randomized study. J Rehabil Res Dev 38: 69-78.
80. Pohl M, Mehrholz J, Ritschel C, Ruckriem S (2002) Speed-dependent treadmill training in ambulatory hemiparetic stroke patients: a randomized controlled trial. Stroke 33: 553-558.
81. Ada L, Dean C, Hall J, Bampton J, Crompton S (2003) A treadmill and overground walking program improves walking in persons residing in the community after stroke: a placebo-controlled, randomized trial. Arch Phys Med Rehabil 84: 1486-1491.
82. Macko R, Ivey F, Forrester L, Hanley D, Sorkin J, et al. (2005) Treadmill exercise rehabilitation improves ambulatory function and cardiovascular fitness in patients with chronic stroke: a randomized, controlled trial. Stroke 36: 2206-2211.
83. Ivey F, Ryan A, Hafer-Macko C, Goldberg A, Macko R (2007) Treadmill aerobic training improves glucose tolerance and indices of insulin sensitivity in disabled stroke survivors: a preliminary report. Stroke 38: 2752-2758.
84. Luft A, Macko R, Forrester L, Villagra F, Ivey F, et al. (2008) Treadmill exercise activates subcortical neural networks and improves walking after stroke: a randomized controlled trial. Stroke 39: 3341-3350.
85. Ivey F, Hafer-Macko C, Ryan A, Macko R (2010) Impaired leg vasodilatory function after stroke: adaptations with treadmill exercise training. Stroke 41: 2913-2917.
86. Ivey F, Ryan A, Hafer-Macko C, Macko R (2011) Improved cerebral vasomotor reactivity after exercise training in hemiparetic stroke survivors. Stroke 42: 1994-2000.
87. Langhammer B, Stanghelle J (2010) Exercise on a treadmill or walking outdoors? A randomized controlled trial comparing effectiveness of two walking exercise programmes late after stroke. Clin Rehabil 24: 46-54.
88. Kuys S, Brauer S, Ada L (2011) Higher-intensity treadmill walking during rehabilitation after stroke in feasible and not detrimental to walking pattern or quality: a pilot randomized trial. Clin Rehabil 25: 316-326.

89. Lau K, Mak M (2011) Speed-dependent treadmill training is effective to improve gait and balance performance in patients with sub-acute stroke. J Rehabil Med 43: 709-713.
90. Olawale O, Jaja S, Anigbogu C, Appiah-Kubi K, Jones-Okai D (2011) Exercise training improves walking function in an African group of stroke survivors: a randomized controlled trial. Clin Rehabil 25: 442-450.
91. Wall J, Turnbull G (1987) Evaluation of out-patient physiotherapy and a home exercise program in the management of gait asymmetry in residual stroke. Neurorehabil Neural Repair 1: 115-123.
92. Wade D, Collen F, Robb G, Warlow C (1992) Physiotherapy intervention late after stroke and mobility. BMJ 304: 609-613.
93. Dean C, Richards C, Malouin F (2000) Task-related circuit training improves performance of locomotor tasks in chronic stroke: a randomized, controlled pilot trial. Arch Phys Med Rehabil 81: 409-417.
94. Green J, Forster A, Bogle S, Young J (2002) Physiotherapy for patients with mobility problems more than 1 year after stroke: a randomised controlled trial. Lancet 359: 199-203.
95. Lin J, Hsieh C, Lo S, Chai H, Liao L (2004) Preliminary study of the effect of low-intensity home-based physical therapy in chronic stroke patients. Kaohsiung J Med Sci 20: 18-23.
96. Salbach N, Mayo N, Wood-Dauphinee S, Hanley J, Richards C, et al. (2004) A task-orientated intervention enhances walking distance and speed in the first year post stroke: a randomized controlled trial. Clin Rehabil 18: 509-519.
97. Salbach N, Mayo N, Robichaud-Ekstrand S, Hanley J, Richards C, et al. (2005) The effect of a task-oriented walking intervention on improving balance self-efficacy poststroke: a randomized, controlled trial. J Am Geriatr Soc 53: 576-582.
98. Pang M, Eng J, Dawson A, McKay H, Harris J (2005) A community-based fitness and mobility exercise program for older adults with chronic stroke: a randomized, controlled trial. J Am Geriatr Soc 53: 1667-1674.
99. Pang M, Harris J, Eng J (2006) A community-based upper-extremity group exercise program improves motor function and performance of functional activities in chronic stroke: a randomized controlled trial. Arch Phys Med Rehabil 87: 1-9.
100. Yang Y, Yen J, Wang R, Yen L, Lieu F (2005) Gait outcomes after additional backward walking training in patients with stroke: a randomized controlled trial. Clin Rehabil 19: 264-273.
101. Yang Y, Wang R, Chen Y, Kao M (2007) Dual-task exercise improves walking ability in chronic stroke: a randomized controlled trial. Arch Phys Med Rehabil 88: 1236-1240.
102. Patil P, Rao S (2011) Effects of Thera-Band elastic resistance-assisted gait training in stroke patients: a pilot study. Eur J Phys Rehabil Med 47: 427-433.
103. Sungkarat S, Fisher B, Kovindha A (2011) Efficacy of an insole shoe wedge and augmented pressure sensor for gait training in individuals with stroke: a randomized controlled trial. Clin Rehabil 25: 360-369.
104. Mandel A, Nymark J, Balmer S, Grinnell D, O'Riain M (1990) Electromyographic versus rhythmic positional biofeedback in computerized gait retraining with stroke patients. Arch Phys Med Rehabil 71: 649-654.
105. Thaut M, McIntosh G, Rice R (1997) Rhythmic facilitation of gait training in hemiparetic stroke rehabilitation. J Neurol Sci 151: 207-212.
106. Schauer M, Mauritz K (2003) Musical motor feedback (MMF) in walking hemiparetic stroke patients: randomized trials of gait improvement. Clin Rehabil 17: 713-722.
107. Argstatter H, Hillecke T, Thaut M, Bolay H (2007) Music therapy in motor rehabilitation. Evaluation of a musicomedical gait training programfor hemiparetic stroke patients [Musiktherapie in der neurologischen Rehabilitation. Evaluation eines musikmedizinischen Behandlungskonzepts für die Gangrehabilitation von hemiparetischen Patienten nach Schlaganfall]. Neurol Rehabil 13: 159-165.
108. Jeong S, Kim M (2007) Effects of a theory-driven music and movement program for stroke survivors in a community setting. Appl Nurs Res 20: 125-131.
109. Thaut M, Leins A, Rice R, Argstatter H, Kenyon G, et al. (2007) Rhythmic auditory stimulation improves gait more than NDT/Bobath training in near-ambulatory patients early poststroke: a single-blind, randomized trial. Neurorehabil Neural Repair 21: 455-459.
110. Lord S, McPherson K, McNaughton H, Rochester L, Weatherall M (2008) How feasible is the attainment of community ambulation after stroke? A pilot randomized controlled trial to evaluate community-based physiotherapy in subacute stroke. Clin Rehabil 22: 215-225.
111. Park H, Oh D, Kim S, Choi J (2011) Effectiveness of community-based ambulation training for walking function of post-stroke hemiparesis: a randomized controlled pilot trial. Clin Rehabil 25: 451-459.

112. Jaffe D, Brown D, Pierson-Carey C, Buckley E, Lew H (2004) Stepping over obstacles to improve walking in individuals with poststroke hemiplegia. J Rehabil Res Dev 41: 283-292.
113. You S, Jang S, Kim Y, Hallett M, Ahn S, et al. (2005) Virtual reality-induced cortical reorganization and associated locomotor recovery in chronic stroke: an experimenter-blind randomized study. Stroke 36: 1166-1171.
114. Lam Y, Man D, Tam S, Weiss P (2006) Virtual reality training for stroke rehabilitation. NeuroRehabilitation 21: 245-253.
115. Yang Y, Tsai M, Chuang T, Sung W, Wang R (2008) Virtual reality-based training improves community ambulation in individuals with stroke: a randomized controlled trial. Gait Posture 28: 201-206.
116. Kim J, Jang S, Kim C, Jung J, You J (2009) Use of virtual reality to enhance balance and ambulation in chronic stroke: a double-blind, randomized controlled study. Am J Phys Med Rehabil 88: 693-701.
117. Mirelman A, Bonato P, Deutsch J (2009) Effects of training with a robot-virtual reality system compared with a robot alone on the gait of individuals after stroke. Stroke 40: 169-174.
118. Mirelman A, Patritti B, Bonato P, Deutsch J (2010) Effects of virtual reality training on gait biomechanics of individuals post-stroke. Gait Posture 31: 433-437.
119. Blennerhassett J, Dite W (2004) Additional task-related practice improves mobility and upper limb function early after stroke: a randomised controlled trial. Aust J Physiother 50: 219-224.
120. Yang Y, Wang R, Lin K, Chu M, Chan R (2006) Task-oriented progressive resistance strength training improves muscle strength and functional performance in individuals with stroke. Clin Rehabil 20: 860-870.
121. Mead G, Greig C, Cunningham I, Lewis S, Dinan S, et al. (2007) Stroke: a randomized trial of exercise or relaxation. J Am Geriatr Soc 55: 892-899.
122. Mudge S, Barber P, Stott N (2009) Circuit-based rehabilitation improves gait endurance but not usual walking activity in chronic stroke: a randomized controlled trial. Arch Phys Med Rehabil 90: 1989-1996.
123. Kalra L, Evans A, Perez I, Melbourn A, Patel A, et al. (2004) Training carers of stroke patients: randomised controlled trial. BMJ 328: 1099.
124. Galvin R, Cusack T, O'Grady E, Murphy T, Stokes E (2011) Family-mediated exercise intervention (FAME): evaluation of a novel form of exercise delivery after stroke. Stroke 42: 681-686.
125. Beckerman H, Becher J, Lankhorst G, Verbeek A, Vogelaar T (1996) The efficacy of thermocoagulation of the tibial nerve and a polypropylene ankle-foot orthosis on spasticity of the leg in stroke patients: results of a randomized clinical trial. Clin Rehabil 10: 112-120.
126. Wright P, Mann G, Swain I (2004) A comparison of electrical stimulation and the conventional ankle foot orthosis in the correction of a dropped foot following stroke. Final report to funder.
127. Erel S, Uygur F, Engin Simsek I, Yakut Y (2011) The effects of dynamic ankle-foot orthoses in chronic stroke patients at three-month follow-up: a randomized controlled trial. Clin Rehabil 25: 515-523.
128. Chu K, Eng J, Dawson A, Harris J, Ozkaplan A, et al. (2004) Water-based exercise for cardiovascular fitness in people with chronic stroke: a randomized controlled trial. Arch Phys Med Rehabil 85: 870-874.
129. Aidar F, Silva A, Reis V, Carneiro A, Carneiro-Cotta S (2007) A study of the quality of life in ischemic vascular accidents and its relation to physical activity [Estudio de la calidad de vida en el accidente vascular isquémico y sur relación con la actividad física]. Rev Neurol 45: 518-522.
130. Noh D, Lim J, Shin H, Paik N (2008) The effect of aquatic therapy on postural balance and muscle strength in stroke survivors--a randomized controlled pilot trial. Clin Rehabil 22: 966-976.
131. Lynch E, Hillier S, Stiller K, Campanella R, Fisher P (2007) Sensory retraining of the lower limb after acute stroke: a randomized controlled pilot trial. Arch Phys Med Rehabil 88: 1101-1107.
132. Yavuzer G, Oken O, Atay M, Stam H (2007) Effects of sensory-amplitude electric stimulation on motor recovery and gait kinematics after stroke: a randomized controlled study. Arch Phys Med Rehabil 88: 710-714.
133. Torriani C, Mota E, Moreira Sales A, Ricci M, Nishida P, et al. (2008) Effect of foot motor and sensorial stimulation hemiparetic in stroke patients. Rev Neurocienc 16: 25-29.
134. Wu H, Lin Y, Hsu M, Liu S, Hsieh C, et al. (2010) Effect of thermal stimulation on upper extremity motor recovery 3 months after stroke. Stroke 41: 2378-2380.

135. Chen J, Lin C, Wei Y, Hsiao J, Liang C (2011) Facilitation of motor and balance recovery by thermal intervention for the paretic lower limb of acute stroke: a single-blind randomized clinical trial. Clin Rehabil 25: 823-832.
136. Merletti R, Zelaschi F, Latella D, Galli M, Angeli S, et al. (1978) A control study of muscle force recovery in hemiparetic patients during treatment with functional electrical stimulation. Scand J Rehabil Med 10: 147-154.
137. Cozean C, Pease W, Hubbell S (1988) Biofeedback and functional electric stimulation in stroke rehabilitation. Arch Phys Med Rehabil 69: 401-405.
138. Winchester P, Montgomery J, Bowman B, Hislop H (1983) Effects of feedback stimulation training and cyclical electrical stimulation on knee extension in hemiparetic patients. Phys Ther 63: 1096-1103.
139. Macdonell R, Triggs W, Leikauskas J, Bourque M, Robb K, et al. (1994) Functional electrical stimulation to the affected lower limb and recovery after cerebral infarction. J Stroke Cerebrovasc Dis 4: 155-160.
140. Bogataj U, Gros N, Kljajic M, Acimovic R, Malezic M (1995) The rehabilitation of gait in patients with hemiplegia: a comparison between conventional therapy and multichannel functional electrical stimulation therapy. Phys Ther 75: 490-502.
141. Burridge J, Taylor P, Hagan S, Wood D, Swain I (1997) The effects of common peroneal stimulation on the effort and speed of walking: a randomized controlled trial with chronic hemiplegic patients. Clin Rehabil 11: 201-210.
142. Heckmann J, Mokrusch T, Krockel A, Warnke S, Neundorfer B (1997) EMG-triggered electrical muscle stimulation in the treatment of central hemiparesis after a stroke. Eur J Phys Med Rehabil 7: 138-141.
143. Tekeoglu Y, Adak B, Goksoy T (1998) Effect of transcutaneous electrical nerve stimulation (TENS) on Barthel Activities of Daily Living (ADL) index score following stroke. Clin Rehabil 12: 277-280.
144. Newsam C, Baker L (2004) Effect of an electric stimulation facilitation program on quadriceps motor unit recruitment after stroke. Arch Phys Med Rehabil 85: 2040-2045.
145. Chen S, Chen Y, Chen C, Lai C, Chiang W, et al. (2005) Effects of surface electrical stimulation on the muscle-tendon junction of spastic gastrocnemius in stroke patients. Disabil Rehabil 27: 105-110.
146. Yan T, Hui-Chan C, Li L (2005) Functional electrical stimulation improves motor recovery of the lower extremity and walking ability of subjects with first acute stroke: a randomized placebo-controlled trial. Stroke 36: 80-85.
147. Yavuzer G, Geler-Kulcu D, Sonel-Tur B, Kutlay S, Ergin S, et al. (2006) Neuromuscular electric stimulation effect on lower-extremity motor recovery and gait kinematics of patients with stroke: a randomized controlled trial. Arch Phys Med Rehabil 87: 536-540.
148. Ng S, Hui-Chan C (2007) Transcutaneous electrical nerve stimulation combined with task-related training improves lower limb functions in subjects with chronic stroke. Stroke 38: 2953-2959.
149. Bakhtiary A, Fatemy E (2008) Does electrical stimulation reduce spasticity after stroke? A randomized controlled study. Clin Rehabil 22: 418-425.
150. Ferrante S, Pedrocchi A, Ferrigno G, Molteni F (2008) Cycling induced by functional electrical stimulation improves the muscular strength and the motor control of individuals with post-acute stroke. Europa Medicophysica-SIMFER 2007 Award Winner. Eur J Phys Rehabil Med 44: 159-167.
151. Janssen T, Beltman J, Elich P, Koppe P, Konijnenbelt H, et al. (2008) Effects of electric stimulation-assisted cycling training in people with chronic stroke. Arch Phys Med Rehabil 89: 463-469.
152. Kojovic J, Djuric-Jovicic M, Dosen S, Popovic M, Popovic D (2009) Sensor-driven four-channel stimulation of paretic leg: functional electrical walking therapy. J Neurosci Methods 181: 100-105.
153. Mesci N, Ozdemir F, Kabayel D, Tokuc B (2009) The effects of neuromuscular electrical stimulation on clinical improvement in hemiplegic lower extremity rehabilitation in chronic stroke: a single-blind, randomised, controlled trial. Disabil Rehabil 31: 2047-2054.
154. Ng SS, Hui-Chan CW (2009) Does the use of TENS increase the effectiveness of exercise for improving walking after stroke? A randomized controlled clinical trial. Clin Rehabil 23: 1093-1103.
155. Hui-Chan C, Ng S, Mak M (2009) Effectiveness of a home-based rehabilitation programme on lower limb functions after stroke. Hong Kong Med J 15: 42-46.

156. Yan T, Hui-Chan C (2009) Transcutaneous electrical stimulation on acupuncture points improves muscle function in subjects after acute stroke: a randomized controlled trial. J Rehabil Med 41: 312-316.
157. Cheng J, Yang Y, Cheng S, Lin P, Wang R (2010) Effects of combining electric stimulation with active ankle dorsiflexion while standing on a rocker board: a pilot study for subjects with spastic foot after stroke. Arch Phys Med Rehabil 91: 505-512.
158. Ambrosini E, Ferrante S, Pedrocchi A, Ferrigno G, Molteni F (2011) Cycling induced by electrical stimulation improves motor recovery in postacute hemiparetic patients: a randomized controlled trial. Stroke 42: 1068-1073.
159. Basmajian J, Kukulka C, Narayan M, Takebe K (1975) Biofeedback treatment of foot-drop after stroke compared with standard rehabilitation technique: effects on voluntary control and strength. Arch Phys Med Rehabil 56: 231-236.
160. Hurd W, Pegram V, Nepomuceno C (1980) Comparison of actual and simulated EMG biofeedback in the treatment of hemiplegic patients. Am J Phys Med 59: 73-82.
161. Binder S, Moll C, Wolf S (1981) Evaluation of electromyographic biofeedback as an adjunct to therapeutic exercise in treating the lower extremities of hemiplegic patients. Phys Ther 61: 886-893.
162. John J (1986) Failure of electrical myofeedback to augment the effects of physiotherapy in stroke. Int J Rehabil Res 9: 35-45.
163. Mulder T, Hulstijn W, Van der Meer J (1986) EMG feedback and the restoration of motor control. A controlled group study of 12 hemiparetic patients. Am J Phys Med 65: 173-188.
164. Colborne G, Olney S, Griffin M (1993) Feedback of ankle joint angle and soleus electromyography in the rehabilitation of hemiplegic gait. Arch Phys Med Rehabil 74: 1100-1106.
165. Intiso D, Santilli V, Grasso M, Rossi R, Caruso I (1994) Rehabilitation of walking with electromyographic biofeedback in foot-drop after stroke. Stroke 25: 1189-1192.
166. Bradley L, Hart B, Mandana S, Flowers K, Riches M, et al. (1998) Electromyographic biofeedback for gait training after stroke. Clin Rehabil 12: 11-22.
167. Jonsdottir J, Cattaneo D, Recalcati M, Regola A, Rabuffetti M, et al. (2010) Task-oriented biofeedback to improve gait in individuals with chronic stroke: motor learning approach. Neurorehabil Neural Repair 24: 478-485.
168. Dean C, Mackey F, Katrak P (2000) Examination of shoulder positioning after stroke: A randomised controlled pilot trial. Aust J Physiother 46: 35-40.
169. Ada L, Goddard E, McCully J, Stavrinos T, Bampton J (2005) Thirty minutes of positioning reduces the development of shoulder external rotation contracture after stroke: a randomized controlled trial. Arch Phys Med Rehabil 86: 230-234.
170. Turton A, Britton E (2005) A pilot randomized controlled trial of a daily muscle stretch regime to prevent contractures in the arm after stroke. Clin Rehabil 19: 600-612.
171. De Jong L, Nieuwboer A, Aufdemkampe G (2006) Contracture preventive positioning of the hemiplegic arm in subacute stroke patients: a pilot randomized controlled trial. Clin Rehabil 20: 656-667.
172. Gustafsson L, McKenna K (2006) A programme of static positional stretches does not reduce hemiplegic shoulder pain or maintain shoulder range of motion--a randomized controlled trial. Clin Rehabil 20: 277-286.
173. Rose V, Shah S (1980) A comparative study on the immediate effects of hand orthosis on reduction of hypertonus. Aust Occup Ther J 34: 59-64.
174. Carey J (1990) Manual stretch: effect on finger movement control and force control in stroke subjects with spastic extrinsic finger flexor muscles. Arch Phys Med Rehabil 71: 888-894.
175. Langlois S, Pederson L, MacKinnon J (1991) The effects of splinting on the spastic hemiplegic hand: report of a feasible study. Can J Occup Ther 58: 17-25.
176. Sheehan J, Winzeler-Mercay U, Mudie M (2006) A randomized controlled pilot study to obtain the best estimate of the size of the effect of a thermoplastic resting splint on spasticity in the stroke-affected wrist and fingers. Clin Rehabil 20: 1032-1037.
177. Lannin N, Cusick A, McCluskey A, Herbert H (2007) Effects of splinting on wrist contracture after stroke: a randomized controlled trial. Stroke 38: 111-116.
178. Bürge E, Kupper D, Finckh A, Ryerson S, Schnider A, et al. (2008) Neutral functional realignment orthosis prevents hand pain in patients with subacute stroke: a randomized trial. Arch Phys Med Rehabil 89: 1857-1862.

179. Heidari M, Eghlidi Z, About Talebi S, Hosseini S, Rahimifard H, et al. (2011) Comparison of mobilizing and immobilizing splints on hand motor function in stroke patients: a randomized clinical trial. QOM University Med Sci J 4: 48-53.
180. Suat E, Engin S, Nilgun B, Yavuz Y, Fatma U (2011) Short- and long-term effects of an inhibitor hand splint in poststroke patients: a randomized controlled trial. Top Stroke Rehabil 18: 231-237.
181. Poole J, Whitney S, Hangeland N, Baker C (1990) The effectiveness of inflatable pressure splints on motor function in stroke patients. Occup Ther J Res 10: 360-366.
182. Feys H, De Weerdt W, Selz B, Cox Steck G, Spichiger R, et al. (1998) Effect of a therapeutic intervention for the hemiplegic upper limb in the acute phase after stroke: a single-blind, randomized, controlled multicenter trial. Stroke 29: 785-792.
183. Feys H, De Weerdt W, Verbeke G, Steck G, Capiau C, et al. (2004) Early and repetitive stimulation of the arm can substantially improve the long-term outcome after stroke: a 5-year follow-up study of a randomized trial. Stroke 35: 924-929.
184. Roper T, Redford S, Tallis R (1999) Intermittent compression for the treatment of the oedematous hand in hemiplegic stroke: a randomized controlled trial. Age Ageing 28: 9-13.
185. Cambier D, de Corte E, Danneels L, Witvrouw E (2003) Treating sensory impairments in the post-stroke upper limb with intermittent pneumatic compression. Results of a preliminary trial. Clin Rehabil 17: 14-20.
186. Platz T, van Kaick S, Mehrholz J, Leidner O, Eickhof C, et al. (2009) Best conventional therapy versus modular impairment-oriented training for arm paresis after stroke: a single-blind, multicenter randomized controlled trial. Neurorehabil Neural Repair 23: 706-716.
187. Hanger H, Whitewood P, Brown G, Ball M, Harper J, et al. (2000) A randomized controlled trial of strapping to prevent post-stroke shoulder pain. Clin Rehabil 14: 370-380.
188. Griffin A, Bernhardt J (2006) Strapping the hemiplegic shoulder prevents development of pain during rehabilitation: a randomized controlled trial. Clin Rehabil 20: 287-295.
189. Appel C, Mayston M, Perry (2011) Feasibility study of a randomized controlled trial protocol to examine clinical effectiveness of shoulder strapping in acute stroke patients. Clin Rehabil 25: 833-843.
190. Van der Lee J, Wagenaar R, Lankhorst G, Vogelaar T, Deville W, et al. (1999) Forced use of the upper extremity in chronic stroke patients: results from a single-blind randomized clinical trial. Stroke 30: 2369-2375.
191. Mudie M, Matyas T (2001) Responses of the densely hemiplegic upper extremity to bilateral training. Neurorehabil Neural Repair 15: 129-140.
192. Platz T, Bock S, Prass K (2001) Reduced skilfulness of arm motor behaviour among motor stroke patients with good clinical recovery: does it indicate reduced automaticity? Can it be improved by unilateral or bilateral training? A kinematic motion analysis study. Neuropsychologia 39: 687-698.
193. Cauraugh J, Kim S (2002) Two coupled motor recovery protocols are better than one: electromyogram-triggered neuromuscular stimulation and bilateral movements. Stroke 33: 1589-1594.
194. Cauraugh J, Kim S (2003) Progress toward motor recovery with active neuromuscular stimulation: muscle activation pattern evidence after a stroke. J Neurol Sci 207: 25-29.
195. Luft A, McCombe-Waller S, Whitall J, Forrester L, Macko R, et al. (2004) Repetitive bilateral arm training and motor cortex activation in chronic stroke: a randomized controlled trial. JAMA 292: 1853-1861.
196. Suputtitada A, Suwanwela N, Tumvitee S (2004) Effectiveness of constraint-induced movement therapy in chronic stroke patients. J Med Assoc Thai 87: 1482-1490.
197. Cauraugh J, Kim S, Duley A (2005) Coupled bilateral movements and active neuromuscular stimulation: intralimb transfer evidence during bimanual aiming. Neurosci Lett 382: 39-44.
198. Desrosiers J, Bourbonnais D, Corriveau H, Gosselin S, Bravo G (2005) Effectiveness of unilateral and symmetrical bilateral task training for arm during the subacute phase after stroke: a randomized controlled trial. Clin Rehabil 19: 581-593.
199. Lum P, Burgar C, Van der Loos M, Shor P, Majmundar M, et al. (2006) MIME robotic device for upper-limb neurorehabilitation in subacute stroke subjects: A follow-up study. J Rehabil Res Dev 43: 631-642.
200. Summers J, Kagerer F, Garry M, Hiraga C, Loftus A, et al. (2007) Bilateral and unilateral movement training on upper limb function in chronic stroke patients: A TMS study. J Neurol Sci 252: 76-82.

201. Cauraugh J, Kim S, Summers J (2008) Chronic stroke longitudinal motor improvements: cumulative learning evidence found in the upper extremity. Cerebrovasc Dis 25: 115-121.
202. McCombe Waller S, Liu W, Whitall J (2008) Temporal and spatial control following bilateral versus unilateral training. Hum Mov Sci 27: 749-758.
203. Morris J, van WF, Joice S, Ogston S, Cole I, et al. (2008) A comparison of bilateral and unilateral upper-limb task training in early poststroke rehabilitation: a randomized controlled trial. Arch Phys Med Rehabil 89: 1237-1245.
204. Cauraugh J, Coombes S, Lodha N, Naik S, Summers J (2009) Upper extremity improvements in chronic stroke: coupled bilateral load training. Restor Neurol Neurosci 27: 17-25.
205. Lin K, Chang Y, Wu C, Chen Y (2009) Effects of constraint-induced therapy versus bilateral arm training on motor performance, daily functions, and quality of life in stroke survivors. Neurorehabil Neural Repair 23: 441-448.
206. Stoykov M, Lewis G, Corcos D (2009) Comparison of bilateral and unilateral training for upper extremity hemiparesis in stroke. Neurorehabil Neural Repair 23: 945-953.
207. Hayner K, Gibson G, Giles G (2010) Comparison of constraint-induced movement therapy and bilateral treatment of equal intensity in people with chronic upper-extremity dysfunction after cerebrovascular accident. Am J Occup Ther 64: 528-539.
208. Lin K, Chen Y, Chen C, Wu C, Chang Y (2010) The effects of bilateral arm training on motor control and functional performance in chronic stroke: a randomized controlled study. Neurorehabil Neural Repair 24: 42-51.
209. Wu C, Hsieh Y, Lin K, Chuang L, Chang Y, et al. (2010) Brain reorganization after bilateral arm training and distributed constraint-induced therapy in stroke patients: a preliminary functional magnetic resonance imaging study. Chang Gung Med J 33: 628-638.
210. Whitall J, Waller S, Sorkin J, Forrester L, Macko R, et al. (2011) Bilateral and unilateral arm training improve motor function through differing neuroplastic mechanisms: a single-blinded randomized controlled trial. Neurorehabil Neural Repair 25: 118-129.
211. Wu C, Chuang L, Lin K, Chen H, Tsay P (2011) Randomized trial of distributed constraint-induced therapy versus bilateral arm training for the rehabilitation of upper-limb motor control and function after stroke. Neurorehabil Neural Repair 25: 130-139.
212. Taub E, Miller N, Novack T, Cook 3rd E, Fleming W, et al. (1993) Technique to improve chronic motor deficit after stroke. Arch Phys Med Rehabil 74: 347-354.
213. Dromerick A, Edwards D, Hahn M (2000) Does the application of constraint-induced movement therapy during acute rehabilitation reduce arm impairment after ischemic stroke? Stroke 31: 2984-2988.
214. Page S, Sisto S, Levine P, Johnston M, Hughes M (2001) Modified constraint induced therapy: a randomized feasibility and efficacy study. J Rehabil Res Dev 38: 583-590.
215. Page S, Sisto S, Johnston M, Levine P (2002) Modified constraint-induced therapy after subacute stroke: a preliminary study. Neurorehabil Neural Repair 16: 290-295.
216. Wittenberg G, Chen R, Ishii K, Bushara K, Eckloff S, et al. (2003) Constraint-induced therapy in stroke: magnetic-stimulation motor maps and cerebral activation. Neurorehabil Neural Repair 17: 48-57.
217. Alberts J, Butler A, Wolf S (2004) The effects of constraint-induced therapy on precision grip: a preliminary study. Neurorehabil Neural Repair 18: 250-258.
218. Atteya A (2004) Effects of modified constraint induced therapy on upper limb function in subacute stroke patients. Neurosciences (Riyadh) 9: 24-29.
219. Page S, Sisto S, Levine P, McGrath R (2004) Efficacy of modified constraint-induced movement therapy in chronic stroke: a single-blinded randomized controlled trial. Arch Phys Med Rehabil 85: 14-18.
220. Ploughman M, Corbett D (2004) Can forced-use therapy be clinically applied after stroke? An exploratory randomized controlled trial. Arch Phys Med Rehabil 85: 1417-1423.
221. Page S, Levine P, Leonard A (2005) Modified constraint-induced therapy in acute stroke: a randomized controlled pilot study. Neurorehabil Neural Repair 19: 27-32.
222. Yen J, Wang R, Chen H, Hong C (2005) Effectiveness of modified constraint-induced movement therapy on upper limb function in stroke subjects. Acta Neurol Taiwan 14: 16-20.
223. Ro T, Noser E, Boake C, Johnson R, Gaber M, et al. (2006) Functional reorganization and recovery after constraint-induced movement therapy in subacute stroke: case reports. Neurocase 12: 50-60.
224. Brogardh C, Sjolund B (2006) Constraint-induced movement therapy in patients with stroke: a pilot study on effects of small group training and of extended mitt use. Clin Rehabil 20: 218-227.

225. Wolf S, Winstein C, Miller J, Taub E, Uswatte G, et al. (2006) Effect of constraint-induced movement therapy on upper extremity function 3 to 9 months after stroke: the EXCITE randomized clinical trial. JAMA 296: 2095-2104.
226. Wolf S, Thompson P, Winstein C, Miller J, Blanton S, et al. (2010) The EXCITE stroke trial: comparing early and delayed constraint-induced movement therapy. Stroke 41: 2309-2315.
227. Boake C, Noser E, Ro T, Baraniuk S, Gaber M, et al. (2007) Constraint-induced movement therapy during early stroke rehabilitation. Neurorehabil Neural Repair 21: 14-24.
228. Lin K, Wu C, Wei T, Lee C, Liu J (2007) Effects of modified constraint-induced movement therapy on reach-to-grasp movements and functional performance after chronic stroke: a randomized controlled study. Clin Rehabil 21: 1075-1086.
229. Wu C, Chen C, Tang S, Lin K, Huang Y (2007) Kinematic and clinical analyses of upper-extremity movements after constraint-induced movement therapy in patients with stroke: a randomized controlled trial. Arch Phys Med Rehabil 88: 964-970.
230. Wu C, Chen C, Tsai W, Lin K, Chou S (2007) A randomized controlled trial of modified constraint-induced movement therapy for elderly stroke survivors: changes in motor impairment, daily functioning, and quality of life. Arch Phys Med Rehabil 88: 273-278.
231. Wu C, Lin K, Chen H, Chen I, Hong W (2007) Effects of modified constraint-induced movement therapy on movement kinematics and daily function in patients with stroke: a kinematic study of motor control mechanisms. Neurorehabil Neural Repair 21: 460-466.
232. Dahl A, Askim T, Stock R, Langorgen E, Lydersen S, et al. (2008) Short- and long-term outcome of constraint-induced movement therapy after stroke: a randomized controlled feasibility trial. Clin Rehabil 22: 436-447.
233. Gauthier L, Taub E, Perkins C, Ortmann M, Mark V, et al. (2008) Remodeling the brain: plastic structural brain changes produced by different motor therapies after stroke. Stroke 39: 1520-1525.
234. Kim D, Cho Y, Hong J, Song J, Chung H, et al. (2008) Effect of constraint-induced movement therapy with modified opposition restriction orthosis in chronic hemiparetic patients with stroke. NeuroRehabilitation 23: 239-244.
235. Myint J, Yuen G, Yu T, Kng C, Wong A, et al. (2008) A study of constraint-induced movement therapy in subacute stroke patients in Hong Kong. Clin Rehabil 22: 112-124.
236. Myint M, Yuen F, Yu K, Kng P, Wong M, et al. (2008) Use of constraint-induced movement therapy in Chinese stroke patients during the sub-acute period. Hong Kong Med J 14: 40-42.
237. Page S, Levine P, Leonard A, Szaflarski J, Kissela B (2008) Modified constraint-induced therapy in chronic stroke: results of a single-blinded randomized controlled trial. Phys Ther 88: 333-340.
238. Sawaki L, Butler A, Leng X, Wassenaar P, Mohammad Y, et al. (2008) Constraint-induced movement therapy results in increased motor map area in subjects 3 to 9 months after stroke. Neurorehabil Neural Repair 22: 505-513.
239. Azab M, Al-Jarrah M, Nazzal M, Maayah M, Sammour M, et al. (2009) Effectiveness of constraint-induced movement therapy (CIMT) as home-based therapy on Barthel Index in patients with chronic stroke. Top Stroke Rehabil 16: 207-211.
240. Brogardh C, Vestling M, Sjolund B (2009) Shortened constraint-induced movement therapy in subacute stroke - no effect of using a restraint: a randomized controlled study with independent observers. J Rehabil Med 41: 231-236.
241. Brogardh C, Lexell J (2010) A 1-year follow-up after shortened constraint-induced movement therapy with and without mitt poststroke. Arch Phys Med Rehabil 91: 460-464.
242. Dromerick A, Lang C, Birkenmeier R, Wagner J, Miller J, et al. (2009) Very Early Constraint-Induced Movement during Stroke Rehabilitation (VECTORS): a single-center RCT. Neurology 73: 195-201.
243. Hammer A, Lindmark B (2009) Is forced use of the paretic upper limb beneficial? A randomized pilot study during subacute post-stroke recovery. Clin Rehabil 23: 424-433.
244. Lin K, Wu C, Liu J, Chen Y, Hsu C (2009) Constraint-induced therapy versus dose-matched control intervention to improve motor ability, basic/extended daily functions, and quality of life in stroke. Neurorehabil Neural Repair 23: 160-165.
245. Woodbury M, Howland D, McGuirk T, Davis S, Senesac C, et al. (2009) Effects of trunk restraint combined with intensive task practice on poststroke upper extremity reach and function: a pilot study. Neurorehabil Neural Repair 23: 78-91.
246. Abu Tariah H, Almalty A, Sbeih, Al-Oraibi S (2010) Constraint induced movement therapy for stroke survivors in Jordon: a home-based model. Int J Ther Rehab 17: 638-646.

247. Lin K, Chung H, Wu C, Liu H, Hsieh Y, et al. (2010) Constraint-induced therapy versus control intervention in patients with stroke: a functional magnetic resonance imaging study. Am J Phys Med Rehabil 89: 177-185.
248. Sun S, Hsu C, Sun H, Hwang C, Yang C, et al. (2010) Combined botulinum toxin type A with modified constraint-induced movement therapy for chronic stroke patients with upper extremity spasticity: a randomized controlled study. Neurorehabil Neural Repair 24: 34-41.
249. Wang Q, Zhao J, Zhu Q, Li J, Meng P (2011) Comparison of conventional therapy, intensive therapy and modified constraint-induced movement therapy to improve upper extremity function after stroke. J Rehabil Med 43: 619-625.
250. Aisen M, Krebs H, Hogan N, McDowell F, Volpe B (1997) The effect of robot-assisted therapy and rehabilitative training on motor recovery following stroke. Arch Neurol 54: 443-446.
251. Volpe B, Krebs H, Hogan N, Edelsteinn L, Driels C, et al. (1999) Robot training enhances motor outcome in patients with stroke maintained over 3 years. Neurology 53: 1874-1876.
252. Volpe B, Krebs H, Hogan N, Edelstein OTR L, Diels C, et al. (2000) A novel approach to stroke rehabilitation: robot-aided sensorimotor stimulation. Neurology 54: 1938-1944.
253. Lum P, Burgar C, Shor P, Majmundar M, Van der Loos M (2002) Robot-assisted movement training compared with conventional therapy techniques for the rehabilitation of upper-limb motor function after stroke. Arch Phys Med Rehabil 83: 952-959.
254. Stein J, Krebs H, Frontera W, Fasoli S, Hughes R, et al. (2004) Comparison of two techniques of robot-aided upper limb exercise training after stroke. Am J Phys Med Rehabil 83: 720-728.
255. Daly J, Hogan N, Perepezko E, Krebs H, Rogers J, et al. (2005) Response to upper-limb robotics and functional neuromuscular stimulation following stroke. J Rehabil Res Dev 42: 723-736.
256. Hesse S, Werner C, Pohl M, Rueckriem S, Mehrholz J, et al. (2005) Computerized arm training improves the motor control of the severely affected arm after stroke: a single-blinded randomized trial in two centers. Stroke 36: 1960-1966.
257. Kahn L, Zygman M, Rymer W, Reinkensmeyer D (2006) Robot-assisted reaching exercise promotes arm movement recovery in chronic hemiparetic stroke: a randomized controlled pilot study. J Neuroeng Rehabil 3: 12.
258. Masiero S, Celia A, Rosati G, Armani M (2007) Robotic-assisted rehabilitation of the upper limb after acute stroke. Arch Phys Med Rehabil 88: 142-149.
259. Mayr A, Kofler M, Saltuari L (2008) [ARMOR: an electromechanical robot for upper limb training following stroke. A prospective randomised controlled pilot study]. Handchir Mikrochir Plast Chir 40: 66-73.
260. Rabadi M, Galgano M, Lynch D, Akerman M, Lesser M, et al. (2008) A pilot study of activity-based therapy in the arm motor recovery post stroke: a randomized controlled trial. Clin Rehabil 22: 1071-1082.
261. Takahashi C, Der-Yeghiaian L, Le V, Motiwala R, Cramer S (2008) Robot-based hand motor therapy after stroke. Brain 131: 425-437.
262. Volpe B, Lynch D, Rykman-Berland A, Ferraro M, Galgano M, et al. (2008) Intensive sensorimotor arm training mediated by therapist or robot improves hemiparesis in patients with chronic stroke. Neurorehabil Neural Repair 22: 305-310.
263. Ellis M, Sukal-Moulton T, Dewald J (2009) Progressive shoulder abduction loading is a crucial element of arm rehabilitation in chronic stroke. Neurorehabil Neural Repair 23: 862-869.
264. Housman S, Scott K, Reinkensmeyer D (2009) A randomized controlled trial of gravity-supported, computer-enhanced arm exercise for individuals with severe hemiparesis. Neurorehabil Neural Repair 23: 505-514.
265. Hu X, Tong K, Song R, Zheng X, Leung W (2009) A comparison between electromyography-driven robot and passive motion device on wrist rehabilitation for chronic stroke. Neurorehabil Neural Repair 23: 837-846.
266. Kutner N, Zhang R, Butler A, Wolf S, Alberts J (2010) Quality-of-life change associated with robotic-assisted therapy to improve hand motor function in patients with subacute stroke: a randomized clinical trial. Phys Ther 90: 493-504.
267. Lo A, Guarino P, Richards L, Haselkorn J, Wittenberg G, et al. (2010) Robot-assisted therapy for long-term upper-limb impairment after stroke. N Engl J Med 362: 1772-1783.
268. Hsieh Y, Wu C, Liao W, Lin K, Wu K, et al. (2011) Effects of treatment intensity in upper limb robot-assisted therapy for chronic stroke: a pilot randomized controlled trial. Neurorehabil Neural Repair 25: 503-511.
269. Masiero S, Armani M, Rosati G (2011) Upper-limb robot-assisted therapy in rehabilitation of acute stroke patients: focused review and results of new randomized controlled trial. J Rehabil Res Dev 48: 355-366.

270. Burgar C, Lum P, Scremin A, Garber S, van der Loos H, et al. (2011) Robot-assisted upper-limb therapy in acute rehabilitation setting following stroke: Department of Veterans Affairs multisite clinical trial. J Rehabil Res Dev 48: 445-458.
271. Conroy S, Whitall J, Dipietro L, Jones-Lush L, Zhan M, et al. (2011) Effect of gravity on robot-assisted motor training after chronic stroke: a randomized trial. Arch Phys Med Rehabil 92: 1754-1761.
272. Page S (2000) Imagery improves upper extremity motor function in chronic stroke patients: a pilot study. Occup Ther J Res 20: 200-215.
273. Page S, Levine P, Sisto S, Johnston M (2001) Mental practice combined with physical practice for upper-limb motor deficit in subacute stroke. Phys Ther 81: 1455-1462.
274. Liu K, Chan C, Lee T, Hui-Chan C (2004) Mental imagery for promoting relearning for people after stroke: a randomized controlled trial. Arch Phys Med Rehabil 85: 1403-1408.
275. Page S, Levine P, Leonard A (2005) Effects of mental practice on affected limb use and function in chronic stroke. Arch Phys Med Rehabil 86: 399-402.
276. Müller K, Bütefisch C, Seitz R, Hömberg V (2007) Mental practice improves hand function after hemiparetic stroke. Restor Neurol Neurosci 25: 501-511.
277. Page S, Levine P, Leonard A (2007) Mental practice in chronic stroke: results of a randomized, placebo-controlled trial. Stroke 38: 1293-1297.
278. Cacchio A, De Blasis E, Necozione S, di Orio F, Santilli V (2009) Mirror therapy for chronic complex regional pain syndrome type 1 and stroke. N Engl J Med 361: 634-636.
279. Liu K (2009) Use of mental imagery to improve task generalisation after a stroke. Hong Kong Med J 15: 37-41.
280. Page S, Szaflarski J, Eliassen J, Pan H, Cramer S (2009) Cortical plasticity following motor skill learning during mental practice in stroke. Neurorehabil Neural Repair 23: 382-388.
281. Riccio I, Iolascon G, Barillari MR, Gimigliano R, Gimigliano F (2010) Mental practice is effective in upper limb recovery after stroke: a randomized single-blind cross-over study. Eur J Phys Rehabil Med 46: 19-25.
282. Braun S, Beurskens A, Kleynen M, Oudelaar B, Schols J, et al. (2012) A multicenter randomized controlled trial to compare subacute 'treatment as usual' with and without mental practice among persons with stroke in Dutch nursing homes. J Am Med Dir Assoc 13: e1-7.
283. Ferreira H, Leite Lopes M, Luiz R, Cardoso L, Andre C (2011) Is visual scanning better than mental practice in hemispatial neglect? Results from a pilot study. Top Stroke Rehabil 18: 155-161.
284. Ietswaart M, Johnston M, Dijkerman HC, Joice S, Scott CL, et al. (2011) Mental practice with motor imagery in stroke recovery: randomized controlled trial of efficacy. Brain 134: 1373-1386.
285. Altschuler E, Wisdom S, Stone L, Foster C, Galasko D, et al. (1999) Rehabilitation of hemiparesis after stroke with a mirror. Lancet 353: 2035-2036.
286. Rothgangel A, Morton A, Van der Hout J, Beurskens A (2004) Phantoms in the brain: spiegeltherapie bij chronische CVA-patiënten: een pilot-study. Ned Tijdschr Fysiother 114: 36-40.
287. Yavuzer G, Selles R, Sezer N, Sutbeyaz S, Bussmann JB, et al. (2008) Mirror therapy improves hand function in subacute stroke: a randomized controlled trial. Arch Phys Med Rehabil 89: 393-398.
288. Cacchio A, De Blasis E, De Blasis V, Santilli V, Spacca G (2009) Mirror therapy in complex regional pain syndrome type 1 of the upper limb in stroke patients. Neurorehabil Neural Repair 23: 792-799.
289. Dohle C, Pullen J, Nakaten A, Kust J, Rietz C, et al. (2009) Mirror therapy promotes recovery from severe hemiparesis: a randomized controlled trial. Neurorehabil Neural Repair 23: 209-217.
290. Michielsen M, Selles R, van der Geest J, Eckhardt M, Yavuzer G, et al. (2011) Motor recovery and cortical reorganization after mirror therapy in chronic stroke patients: a phase II randomized controlled trial. Neurorehabil Neural Repair 25: 223-233.
291. Carey J, Kimberley T, Lewis S, Auerbach E, Dorsey L, et al. (2002) Analysis of fMRI and finger tracking training in subjects with chronic stroke. Brain 125: 773-788.
292. Piron L, Tonin P, Atzori A, Zucconi C, Massaro C, et al. (2003) The augmented-feedback rehabilitation technique facilitates the arm motor recovery in patients after a recent stroke. Stud Health Technol Inform 94: 265-267.

293. Piron L, Tombolini A, Turolla C, Zucconi C, Agostini M, et al. (2007) Reinforced feedback in virtual environment facilitates the arm motor recovery in patients after a recent stroke. IEEE Xplore: 121-123.
294. Jang S, You S, Hallett M, Cho Y, Park C, et al. (2005) Cortical reorganization and associated functional motor recovery after virtual reality in patients with chronic stroke: an experimenter-blind preliminary study. Arch Phys Med Rehabil 86: 2218-2223.
295. Broeren J, Claesson L, Goude D, Rydmark M, Sunnerhagen K (2008) Virtual rehabilitation in an activity centre for community-dwelling persons with stroke. The possibilities of 3-dimensional computer games. Cerebrovasc Dis 26: 289-296.
296. Crosbie J, Lennon S, McDonough S (2007) Virtual reality in the rehabilitation of the upper limb following hemiplegic stroke: a pilot randomised controlled trial (RCT). UK Stroke Forum Conference 2007: 9.
297. Piron L, Turolla A, Tonin P, Piccione F, Lain L, et al. (2008) Satisfaction with care in post-stroke patients undergoing a telerehabilitation programme at home. J Telemed Telecare 14: 257-260.
298. Yavuzer G, Senel A, Atay M, Stam H (2008) "Playstation eyetoy games" improve upper extremity-related motor functioning in subacute stroke: a randomized controlled clinical trial. Eur J Phys Rehabil Med 44: 237-244.
299. Piron L, Turolla A, Agostini M, Zucconi C, Cortese F, et al. (2009) Exercises for paretic upper limb after stroke: a combined virtual-reality and telemedicine approach. J Rehabil Med 41: 1016-1102.
300. Piron L, Turolla A, Agostini M, Zucconi CS, Ventura L, et al. (2010) Motor learning principles for rehabilitation: a pilot randomized controlled study in poststroke patients. Neurorehabil Neural Repair 24: 501-508.
301. Sucar L, Luis R, Leder R, Hernandez J, Sanchez I (2010) Gesture therapy: a vision-based system for upper extremity stroke rehabilitation. Conf Proc IEEE Eng Med Biol Soc 2010: 3690-3693.
302. Saposnik G, Teasell R, Mamdani M, Hall J, McIlroy W, et al. (2010) Effectiveness of virtual reality using Wii gaming technology in stroke rehabilitation: a pilot randomized clinical trial and proof of principle. Stroke 41: 1477-1484.
303. Carey J, Durfee W, Bhatt E, Nagpal A, Weinstein S, et al. (2007) Comparison of finger tracking versus simple movement training via telerehabilitation to alter hand function and cortical reorganization after stroke. Neurorehabil Neural Repair 21: 216-232.
304. Da Silva Cameirao M, Bermúdez I Badia S, Duarte E, Verschure P (2011) Virtual reality based rehabilitation speeds up functional recovery of the upper extremities after stroke: a randomized controlled pilot study in the acute phase of stroke using the Rehabilitation Gaming System. Restor Neurol Neurosci 29: 287-298.
305. Fischer H, Stubblefield K, Kline T, Luo X, Kenyon R, et al. (2007) Hand rehabilitation following stroke: a pilot study of assisted finger extension training in a virtual environment. Top Stroke Rehabil 14: 1-12.
306. Carmeli E, Peleg S, Bartur G, Elbo E, Vatine J (2010) HandTutor(TM) enhanced hand rehabilitation after stroke - a pilot study. Physiother Res Int 16: 191-200.
307. Bowman B, Baker L, Waters R (1979) Positional feedback and electrical stimulation: an automated treatment for the hemiplegic wrist. Arch Phys Med Rehabil 60: 497-502.
308. Baker L, Parker K (1986) Neuromuscular electrical stimulation of the muscles surrounding the shoulder. Phys Ther 66: 1930-1937.
309. Leandri M, Parodi C, Corrieri N, Rigardo S (1990) Comparison of TENS treatments in hemiplegic shoulder pain. Scand J Rehabil Med 22: 69-71.
310. Faghri P, Rodgers M, Glaser R, Bors J, Ho C, et al. (1994) The effects of functional electrical stimulation on shoulder subluxation, arm function recovery, and shoulder pain in hemiplegic stroke patients. Arch Phys Med Rehabil 75: 73-79.
311. Faghri P (1997) The effects of neuromuscular stimulation-induced muscle contraction versus elevation on hand edema in CVA patients. J Hand Ther 10: 29-34.
312. King T (1996) The effect of neuromuscular electrical stimulation in reducing tone. Am J Occup Ther 50: 62-64.
313. Chae J, Bethoux F, Bohine T, Dobos L, Davis T, et al. (1998) Neuromuscular stimulation for upper extremity motor and functional recovery in acute hemiplegia. Stroke 29: 975-979.
314. Francisco G, Chae J, Chawla H, Kirshblum S, Zorowitz R, et al. (1998) Electromyogram-triggered neuromuscular stimulation for improving the arm function of acute stroke survivors: a randomized pilot study. Arch Phys Med Rehabil 79: 570-575.

315. Sonde L, Gip C, Fernaeus S, Nilsson C, Viitanen M (1998) Stimulation with low frequency (1.7 Hz) transcutaneous electric nerve stimulation (low-tens) increases motor function of the post-stroke paretic arm. Scand J Rehabil Med 30: 95-99.
316. Sonde L, Kalimo H, Fernaeus S, Viitanen M (2000) Low TENS treatment on post-stroke paretic arm: a three-year follow-up. Clin Rehabil 14: 14-19.
317. Linn S, Granat M, Lees K (1999) Prevention of shoulder subluxation after stroke with electrical stimulation. Stroke 30: 963-968.
318. Powell J, Pandyan A, Granat M, Cameron M, Stott D (1999) Electrical stimulation of wrist extensors in poststroke hemiplegia. Stroke 30: 1384-1389.
319. Cauraugh J, Light K, Kim S, Thigpen M, Behrman A (2000) Chronic motor dysfunction after stroke: recovering wrist and finger extension by electromyography-triggered neuromuscular stimulation. Stroke 31: 1360-1364.
320. Wang R, Chan R, Tsai M (2000) Functional electrical stimulation on chronic and acute hemiplegic shoulder subluxation. Am J Phys Med Rehabil 79: 385-390.
321. Wang R, Yang Y, Tsai M, Wang W, Chan R (2002) Effects of functional electric stimulation on upper limb motor function and shoulder range of motion in hemiplegic patients. Am J Phys Med Rehabil 81: 283-290.
322. Cauraugh J, Kim S (2003) Chronic stroke motor recovery: duration of active neuromuscular stimulation. J Neurol Sci 215: 13-19.
323. Cauraugh J, Kim S (2003) Stroke motor recovery: active neuromuscular stimulation and repetitive practice schedules. J Neurol Neurosurg Psychiatry 74: 1562-1566.
324. Popovic M, Popovic D, Sinkjaer T, Stefanovic A, Schwirtlich L (2003) Clinical evaluation of functional electrical therapy in acute hemiplegic subjects. J Rehabil Res Dev 40: 443-453.
325. De Kroon J, IJzerman M, Lankhorst G, Zilvold G (2004) Electrical stimulation of the upper limb in stroke: stimulation of the extensors of the hand vs. alternate stimulation of flexors and extensors. Am J Phys Med Rehabil 83: 592-600.
326. Kimberley T, Lewis S, Auerbach E, Dorsey L, Lojovich J, et al. (2004) Electrical stimulation driving functional improvements and cortical changes in subjects with stroke. Exp Brain Res 154: 450-460.
327. Gabr U, Levine P, Page S (2005) Home-based electromyography-triggered stimulation in chronic stroke. Clin Rehabil 19: 737-745.
328. Mann G, Burridge J, Malone L, Strike P (2005) A pilot study to investigate the effects of electrical stimulation on recovery of hand function and sensation in subacute stroke patients. Neuromodulation 8: 193-202.
329. Popovic M, Thrasher T, Zivanovic V, Takaki J, Hajek V (2005) Neuroprosthesis for retraining reaching and grasping functions in severe hemiplegic patients. Neuromodulation 8: 58-72.
330. Ring H, Rosenthal N (2005) Controlled study of neuroprosthetic functional electrical stimulation in sub-acute post-stroke rehabilitation. J Rehabil Med 37: 32-36.
331. Church C, Price C, Pandyan A, Huntley S, Curless R, et al. (2006) Randomized controlled trial to evaluate the effect of surface neuromuscular electrical stimulation to the shoulder after acute stroke. Stroke 37: 2995-3001.
332. Hara Y, Ogawa S, Muraoka Y (2006) Hybrid power-assisted functional electrical stimulation to improve hemiparetic upper-extremity function. Arch Phys Med Rehabil 85: 977-985.
333. Alon G, Levitt A, McCarthy P (2007) Functional electrical stimulation enhancement of upper extremity functional recovery during stroke rehabilitation: a pilot study. Neurorehabil Neural Repair 21: 207-215.
334. Bhatt E, Nagpal A, Greer K, Grunewald T, Steele J, et al. (2007) Effect of finger tracking combined with electrical stimulation on brain reorganization and hand function in subjects with stroke. Exp Brain Res 182: 435-447.
335. Hemmen B, Seelen H (2007) Effects of movement imagery and electromyography-triggered feedback on arm hand function in stroke patients in the subacute phase. Clin Rehabil 21: 587-594.
336. Kowalczewski J, Gritsenko V, Ashworth N, Ellaway P, Prochazka A (2007) Upper-extremity functional electric stimulation-assisted exercises on a workstation in the subacute phase of stroke recovery. Arch Phys Med Rehabil 88: 833-839.
337. McDonnell M, Hillier S, Miles T, Thompson P, Ridding M (2007) Influence of combined afferent stimulation and task-specific training following stroke: a pilot randomized controlled trial. Neurorehabil Neural Repair 21: 435-443.

338. Alon G, Levitt A, McCarthy P (2008) Functional electrical stimulation (FES) may modify the poor prognosis of stroke survivors with severe motor loss of the upper extremity: a preliminary study. Am J Phys Med Rehabil 87: 627-636.
339. Barker R, Brauer S, Carson R (2008) Training of reaching in stroke survivors with severe and chronic upper limb paresis using a novel nonrobotic device: a randomized clinical trial. Stroke 39: 1800-1807.
340. Barker R, Brauer S, Carson R (2009) Training-induced changes in the pattern of triceps to biceps activation during reaching tasks after chronic and severe stroke. Exp Brain Res 196: 483-496.
341. De Kroon J, IJzerman M (2008) Electrical stimulation of the upper extremity in stroke: cyclic versus EMG-triggered stimulation. Clin Rehabil 22: 690-697.
342. Hara Y, Ogawa S, Tsujiuchi K, Muraoka Y (2008) A home-based rehabilitation program for the hemiplegic upper extremity by power-assisted functional electrical stimulation. Disabil Rehabil 30: 296-304.
343. Shin H, Cho S, Jeon H, Lee Y, Song J, et al. (2008) Cortical effect and functional recovery by the electromyography-triggered neuromuscular stimulation in chronic stroke patients. Neurosci Lett 442: 174-179.
344. Thrasher T, Zivanovic V, McIlroy W, Popovic M (2008) Rehabilitation of reaching and grasping function in severe hemiplegic patients using functional electrical stimulation therapy. Neurorehabil Neural Repair 22: 706-714.
345. Chan M, Tong R, Chung K (2009) Bilateral upper limb training with functional electric stimulation in patients with chronic stroke. Neurorehabil Neural Repair 23: 357-365.
346. Klaiput A, Kitisomprayoonkul W (2009) Increased pinch strength in acute and subacute stroke patients after simultaneous median and ulnar sensory stimulation. Neurorehabil Neural Repair 23: 351-356.
347. Mangold S, Schuster C, Keller T, Zimmermann-Schlatter A, Ettlin T (2009) Motor training of upper extremity with functional electrical stimulation in early stroke rehabilitation. Neurorehabil Neural Repair 23: 184-190.
348. Hsu S, Hu M, Wang Y, Yip P, Chiu J, et al. (2010) Dose-response relation between neuromuscular electrical stimulation and upper-extremity function in patients with stroke. Stroke 41: 821-824.
349. Koyuncu E, Nakipoglu-Yüzer G, Dogan A, Ozgirgin N (2010) The effectiveness of functional electrical stimulation for the treatment of shoulder subluxation and shoulder pain in hemiplegic patients: A randomized controlled trial. Disabil Rehabil 32: 560-566.
350. Fil A, Armutlu K, Atay A, Kerimoglu U, Elibol B (2011) The effect of electrical stimulation in combination with Bobath techniques in the prevention of shoulder subluxation in acute stroke patients. Clin Rehabil 25: 51-59.
351. Lin Z, Yan T (2011) Long-term effectiveness of neuromuscular electrical stimulation for promoting motor recovery of the upper extremity after stroke. J Rehabil Med 43: 506-510.
352. Sentandreu Mano T, Salom Terradez J, Tomas J, Melendez Moral J, Fuente Fernandez T, et al. (2011) [Electrical stimulation in the treatment of the spastic hemiplegic hand after stroke: a ramdomized study]. Med Clin (Barc) 137: 297-301.
353. Shindo K, Fujiwara T, Hara J, Oba H, Hotta F, et al. (2011) Effectiveness of hybrid assistive neuromuscular dynamic stimulation therapy in patients with subacute stroke: a randomized controlled pilot trial. Neurorehabil Neural Repair 25: 830-837.
354. Tarkka I, Pitkänen K, Popovic D, Vanninen R, Könönen M (2011) Functional electrical therapy for hemiparesis alleviates disability and enhances neuroplasticity. Tohoku J Exp Med 225: 71-76.
355. Smith K (1979) Biofeedback in strokes. Aust J Physiother 25: 155-161.
356. Greenberg S, Fowler Jr R (1980) Kinesthetic biofeedback: a treatment modality for elbow range of motion in hemiplegia. Am J Occup Ther 34: 738-743.
357. Basmajian J, Gowland C, Brandstater M, Swanson L, Trotter J (1982) EMG feedback treatment of upper limb in hemiplegic stroke patients: a pilot study. Arch Phys Med Rehabil 63: 613-616.
358. Williams J (1982) Use of electromyographic biofeedback for pain reduction in the spastic hemiplegic shoulder: a pilot study. Physiother Can 34: 327-333.
359. Inglis J, Donald M, Monga T, Sproule M, Young M (1984) Electromyographic biofeedback and physical therapy of the hemiplegic upper limb. Arch Phys Med Rehabil 65: 755-759.
360. Basmajian J, Gowland C, Finlayson M, Hall A, Swanson L, et al. (1987) Stroke treatment: comparison of integrated behavioral-physical therapy vs traditional physical therapy programs. Arch Phys Med Rehabil 68: 267-272.
361. Crow J, Lincoln N, Nouri F, De WW (1989) The effectiveness of EMG biofeedback in the treatment of arm function after stroke. Int Disabil Stud 11: 155-160.

362. Bate P, Matyas T (1992) Negative transfer of training following brief practice of elbow tracking movements with electromyographic feedback from spastic antagonists. Arch Phys Med Rehabil 73: 1050-1058.
363. Armagan O, Tascioglu F, Oner C (2003) Electromyographic biofeedback in the treatment of the hemiplegic hand: a placebo-controlled study. Am J Phys Med Rehabil 82: 856-861.
364. Dogan-Aslan M, Nakipoglu-Yuzer G, Dogan A, Karabay I, Ozgirgin N (2010) The effect of electromyographic biofeedback treatment in improving upper extremity functioning of patients with hemiplegic stroke. J Stroke Cerebrovasc Dis 21: 187-192.
365. Michaelsen S, Levin M (2004) Short-term effects of practice with trunk restraint on reaching movements in patients with chronic stroke: a controlled trial. Stroke 35: 1914-1919.
366. Michaelsen S, Dannenbaum R, Levin M (2006) Task-specific training with trunk restraint on arm recovery in stroke: randomized control trial. Stroke 37: 186-192.
367. Thielman G (2010) Rehabilitation of reaching poststroke: a randomized pilot investigation of tactile versus auditory feedback for trunk control. J Neurol Phys Ther 34: 138-144.
368. Heldmann B, Kerkhoff G, Struppler A, Havel P, Jahn T (2000) Repetitive peripheral magnetic stimulation alleviates tactile extinction. Neuroreport 11: 3193-3198.
369. Byl N, Roderick J, Mohamed O, Hanny M, Kotler J, et al. (2003) Effectiveness of sensory and motor rehabilitation of the upper limb following the principles of neuroplasticity: patients stable poststroke. Neurorehabil Neural Repair 17: 176-191.
370. Chen J, Liang C, Shaw F (2005) Facilitation of sensory and motor recovery by thermal intervention for the hemiplegic upper limb in acute stroke patients: a single-blind randomized clinical trial. Stroke 36: 2665-2669.
371. Byl N, Pitsch E, Abrams G (2008) Functional outcomes can vary by dose: learning-based sensorimotor training for patients stable poststroke. Neurorehabil Neural Repair 22: 494-504.
372. Wolny T, Saulicz E, Gnat R, Kokosz M (2010) Butler's neuromobilizations combined with proprioceptive neuromuscular facilitation are effective in reducing of upper limb sensory in late-stage stroke subjects: a three-group randomized trial. Clin Rehabil 24: 810-821.
373. Carey L, Macdonell R, Matyas T (2011) SENSe: Study of the Effectiveness of Neurorehabilitation on Sensation: a randomized controlled trial. Neurorehabil Neural Repair 25: 304-313.
374. Hunter S, Hammett L, Ball S, Smith N, Anderson C, et al. (2011) Dose-response study of mobilisation and tactile stimulation therapy for the upper extremity early after stroke: a phase I trial. Neurorehabil Neural Repair 25: 314-322.
375. Inaba M, Edberg E, Montgomery J, Gillis MK (1973) Effectiveness of functional training, active exercise, and resistive exercise for patients with hemiplegia. PhysTher 53: 28-35.
376. Glasser L (1986) Effects of isokinetic training on the rate of movement during ambulation in hemiparetic patients. Phys Ther 66: 673-676.
377. Kim C, Eng J, MacIntyre D, Dawson A (2001) Effects of isokinetic strength training on walking in persons with stroke: a double-blind controlled pilot study. J Stroke Cerebrovasc Dis 10: 265-273.
378. Bourbonnais D, Bilodeau S, Lepage Y, Beaudoin N, Gravel D, et al. (2002) Effect of force-feedback treatments in patients with chronic motor deficits after a stroke. Am J Phys Med Rehabil 81: 890-897.
379. Carr M, Jones J (2003) Physiological effects of exercise on stroke survivors. Top Stroke Rehabil 9: 57-64.
380. Moreland J, Goldsmith C, Huijbregts M, Anderson R, Prentice D, et al. (2003) Progressive resistance strengthening exercises after stroke: a single-blind randomized controlled trial. Arch Phys Med Rehabil 84: 1433-1440.
381. Ouellette M, LeBrasseur N, Bean J, Phillips E, Stein J, et al. (2004) High-intensity resistance training improves muscle strength, self-reported function, and disability in long-term stroke survivors. Stroke 35: 1404-1409.
382. De Boissezon X, Burlot S, Glezes S, Roques C, Marque P (2005) A randomized controlled trial to compare isokinetic and conventional muscular strengthening in poststroke patients. Isokinet Exerc Sci: 91-92.
383. Akbari A, Karimi H (2006) The effect of strengthening exercises in exaggerated muscle tonicity in chronic hemiparesis following stroke. J Med Sci 6: 382-388.
384. Tihanyi T, Horvath M, Fazekas G, Hortobagyi T, Tihanyi J (2007) One session of whole body vibration increases voluntary muscle strength transiently in patients with stroke. Clin Rehabil 21: 782-793.
385. Bale M, Strand L (2008) Does functional strength training of the leg in subacute stroke improve physical performance? A pilot randomized controlled trial. Clin Rehabil 22: 911-921.

386. Flansbjer U, Miller M, Downham D, Lexell J (2008) Progressive resistance training after stroke: effects on muscle strength, muscle tone, gait performance and perceived participation. J Rehabil Med 40: 42-48.
387. Lee M, Kilbreath S, Singh M, Zeman B, Lord S, et al. (2008) Comparison of effect of aerobic cycle training and progressive resistance training on walking ability after stroke: a randomized sham exercise-controlled study. J Am Geriatr Soc 56: 976-985.
388. Lee M, Kilbreath S, Singh M, Zeman B, Davis G (2010) Effect of progressive resistance training on muscle performance after chronic stroke. Med Sci Sports Exerc 42: 23-34.
389. Page S, Levine P, Teepen J, Hartman E (2008) Resistance-based, reciprocal upper and lower limb locomotor training in chronic stroke: a randomized, controlled crossover study. Clin Rehabil 22: 610-617.
390. Singh S (2008) Closed versus open kinematic chain exercises on gait performance in subacute stroke patients. Physiother Occup Ther J 1: 73-89.
391. Sims J, Galea M, Taylor N, Dodd K, Jespersen S, et al. (2009) Regenerate: assessing the feasibility of a strength-training program to enhance the physical and mental health of chronic post stroke patients with depression. Int J Geriatr Psychiatry 24: 76-83.
392. Cooke E, Tallis R, Clark A, Pomeroy V (2010) Efficacy of functional strength training on restoration of lower-limb motor function early after stroke: phase I randomized controlled trial. Neurorehabil Neural Repair 24: 88-96.
393. Tihanyi J, Di Giminiani R, Tihanyi T, Gyulai G, Trzaskoma L, et al. (2010) Low resonance frequency vibration affects strength of paretic and non-paretic leg differently in patients with stroke. Acta Physiol Hung 97: 172-182.
394. Lippert-Grüner M, Grüner M (1999) Muskelkrafttraining in der Rehabilitation des zentral paretischen Armes. Neurol Rehabil 5: 275-279.
395. Thielman G, Dean C, Gentile A (2004) Rehabilitation of reaching after stroke: task-related training versus progressive resistive exercise. Arch Phys Med Rehabil 85: 1613-1618.
396. Winstein C, Rose D, Tan S, Lewthwaite R, Chui H, et al. (2004) A randomized controlled comparison of upper-extremity rehabilitation strategies in acute stroke: A pilot study of immediate and long-term outcomes. Arch Phys Med Rehabil 85: 620-628.
397. Thielman G, Kaminski T, Gentile A (2008) Rehabilitation of reaching after stroke: comparing 2 training protocols utilizing trunk restraint. Neurorehabil Neural Repair 22: 697-705.
398. Donaldson C, Tallis R, Miller S, Sunderland A, Lemon R, et al. (2009) Effects of conventional physical therapy and functional strength training on upper limb motor recovery after stroke: a randomized phase II study. Neurorehabil Neural Repair 23: 389-397.
399. Potempa K, Lopez M, Braun L, Szidon J, Fogg L, et al. (1995) Physiological outcomes of aerobic exercise training in hemiparetic stroke patients. Stroke 26: 101-105.
400. Katz-Leurer M, Carmeli E, Shochina M (2003) The effect of early aerobic training on independence six months post stroke. Clin Rehabil 17: 735-741.
401. Katz-Leurer M, Shochina M (2007) The influence of autonomic impairment on aerobic exercise outcome in stroke patients. NeuroRehabilitation 22: 267-272.
402. Kamps A, Schüle K (2005) Cyclic movement training of the lower limb in stroke rehabilitation. Neurol Rehabil 11: S1-S12.
403. Lennon O, Carey A, Gaffney N, Stephenson J, Blake C (2008) A pilot randomized controlled trial to evaluate the benefit of the cardiac rehabilitation paradigm for the non-acute ischaemic stroke population. Clin Rehabil 22: 125-133.
404. Quaney B, Boyd L, McDowd J, Zahner L, He J, et al. (2009) Aerobic exercise improves cognition and motor function poststroke. Neurorehabil Neural Repair 23: 879-885.
405. Dobke B, Schüle K, Diehl W, Kaiser T (2010) Apparativ-assistive Bewegungstherapie in der Schlaganfallrehabilitation. Neurol Rehabil 16: 173-185.
406. Toledano-Zarhi A, Tanne D, Carmeli E, Katz-Leurer M (2011) Feasibility, safety and efficacy of an early aerobic rehabilitation program for patients after minor ischemic stroke: A pilot randomized controlled trial. NeuroRehabilitation 28: 85-90.
407. Richards C, Malouin F, Wood-Dauphinee S, Williams J, Bouchard J, et al. (1993) Task-specific physical therapy for optimization of gait recovery in acute stroke patients. Arch Phys Med Rehabil 74: 612-620.
408. Duncan P, Richards L, Wallace D, Stoker-Yates J, Pohl P, et al. (1998) A randomized, controlled pilot study of a home-based exercise program for individuals with mild and moderate stroke. Stroke 29: 2055-2060.

409. Teixeira-Salmela L, Olney S, Nadeau S, Brouwer B (1999) Muscle strengthening and physical conditioning to reduce impairment and disability in chronic stroke survivors. Arch Phys Med Rehabil 80: 1211-1218.
410. Rimmer J, Riley B, Creviston T, Nicola T (2000) Exercise training in a predominantly African-American group of stroke survivors. Med Sci Sports Exerc 32: 1990-1996.
411. Duncan P, Studenski S, Richards L, Gollub S, Lai S, et al. (2003) Randomized clinical trial of therapeutic exercise in subacute stroke. Stroke 34: 2173-2180.
412. Studenski S, Duncan P, Perera S, Reker D, Lai S, et al. (2005) Daily functioning and quality of life in a randomized controlled trial of therapeutic exercise for subacute stroke survivors. Stroke 36: 1764-1770.
413. Lai S, Studenski S, Richards L, Perera S, Reker D, et al. (2006) Therapeutic exercise and depressive symptoms after stroke. J Am Geriatr Soc 54: 240-247.
414. Olney S, Nymark J, Brouwer B, Culham E, Day A, et al. (2006) A randomized controlled trial of supervised versus unsupervised exercise programs for ambulatory stroke survivors. Stroke 37: 476-481.
415. Letombe A, Cornille C, Delahaye H, Khaled A, Morice O, et al. (2010) Early post-stroke physical conditioning in hemiplegic patients: a preliminary study. Ann Phys Rehabil Med 53: 632-642.
416. Outermans J, Van Peppen R, Wittink H, Takken T, Kwakkel G (2010) Effects of a high-intensity task-oriented training on gait performance early after stroke: a pilot study. Clin Rehabil 24: 979-987.
417. Smania N, Girardi F, Domenicali C, Lora E, Aglioti S (2000) The rehabilitation of limb apraxia: a study in left-brain-damaged patients. Arch Phys Med Rehabil 81: 379-388.
418. Smania N, Aglioti SM, Girardi F, Tinazzi M, Fiaschi A, et al. (2006) Rehabilitation of limb apraxia improves daily life activities in patients with stroke. Neurology 67: 2050-2052.
419. Jongbloed L, Morgan D (1991) An investigation of involvement in leisure activities after a stroke. Am J Occup Ther 45: 420-427.
420. Drummond A, Walker M (1995) A randomized controlled trial of leisure rehabilitation after stroke. Clin Rehabil 9: 283-290.
421. Drummond A, Walker M (1996) Generalisation of the effects of leisure rehabilitation for stroke patients. Br J Occup Ther 59: 330-334.
422. Parker C, Gladman J, Drummond A, Dewey M, Lincoln N, et al. (2001) A multicentre randomized controlled trial of leisure therapy and conventional occupational therapy after stroke. TOTAL Study Group. Trial of Occupational Therapy and Leisure. Clin Rehabil 15: 42-52.
423. Nour K, Desrosiers J, Gauthier P, Carbonneau H (2002) Impact of a home leisure educadtional program for older adults who have had a stroke (Home Leisure Educational Program). Ther Recr J 36: 48-64.
424. Desrosiers J, Noreau L, Rochette A, Carbonneau H, Fontaine L, et al. (2007) Effect of a home leisure education program after stroke: a randomized controlled trial. Arch Phys Med Rehabil 88: 1095-1100.
425. Sütbeyaz S, Koseoglu F, Inan L, Coskun O (2010) Respiratory muscle training improves cardiopulmonary function and exercise tolerance in subjects with subacute stroke: a randomized controlled trial. Clin Rehabil 24: 240-250.
426. Britto R, Rezende N, Marinho K, Torres J, Parreira V, et al. (2011) Inspiratory muscular training in chronic stroke survivors: a randomized controlled trial. Arch Phys Med Rehabil 92: 184-190.
427. Stern P, McDowell F, Miller J, Robinson M (1970) Effects of facilitation exercise techniques in stroke rehabilitation. Ann Phys Rehabil Med 51: 526-531.
428. Smith D, Goldenberg E, Ashburn A, Kinsella G, Sheikh K, et al. (1981) Remedial therapy after stroke: a randomised controlled trial. Br med J (Clin Res Ed) 282: 517-520.
429. Sivenius S, Pyrorala K, Heinonen O, Salonen J, Riekkinen P (1985) The significance of intensity of rehabilitation of stroke--a controlled trial. Stroke 16: 928-931.
430. Young J, Forster A (1991) The Bradford community stroke trial: eight week results. Clin Rehabil 5: 283-292.
431. Young J, Forster A (1992) The Bradford community stroke trial: results at six months. BMJ 304: 1085-1089.
432. Sunderland A, Tinson D, Bradley E, Fletcher D, Langton Hewer R, et al. (1992) Enhanced physical therapy improves recovery of arm function after stroke. A randomised controlled trial. J Neurol Neurosurg Psychiatry 55: 530-535.
433. Werner R, Kessler S (1996) Effectiveness of an intensive outpatient rehabilitation program for postacute stroke patients. Am J Phys Med Rehabil 75: 114-120.

434. Logan P, Ahern J, Gladman J, Lincoln N (1997) A randomized controlled trial of enhanced Social Service occupational therapy for stroke patients. Clin Rehabil 11: 107-113.
435. Baskett J, Broad J, Reekie G, Hocking C, Green G (1999) Shared responsibility for ongoing rehabilitation: a new approach to home-based therapy after stroke. Clin Rehabil 13: 23-33.
436. Kwakkel G, Wagenaar R, Twisk J, Lankhorst G, Koetsier J (1999) Intensity of leg and arm training after primary middle-cerebral-artery stroke: a randomised trial. Lancet 354: 191-196.
437. Lincoln N, Parry R, Vass C (1999) Randomized, controlled trial to evaluate increased intensity of physiotherapy treatment of arm function after stroke. Stroke 30: 573-579.
438. Walker M, Gladman J, Lincoln N, Siemonsma P, Whiteley T (1999) Occupational therapy for stroke patients not admitted to hospital: a randomised controlled trial. Lancet 354: 278-280.
439. Partridge C, Mackenzie M, Edwards S, Reid A, Jayawardena S, et al. (2000) Is dosage of physiotherapy a critical factor in deciding patterns of recovery from stroke: a pragmatic randomized controlled trial. Physiother Res Int 5: 230-240.
440. Gilbertson L, Langhorne P, Walker A, Allen A, Murray G (2000) Domiciliary occupational therapy for patients with stroke discharged from hospital: randomised controlled trial. BMJ 320: 603-606.
441. Andersen H, Eriksen K, Brown A, Schultz-Larsen K, Forchhammer B (2002) Follow-up services for stroke survivors after hospital discharge--a randomized control study. Clin Rehabil 16: 593-603.
442. Slade A, Tennant A, Chamerlain M (2002) A randomised controlled trial to determine the effect of intensity of therapy upon length of stay in a neurological rehabilitation setting. J Rehabil Med 34: 260-266.
443. Di Lauro A, Pellegrino L, Savastano G, Ferraro C, Fusco M, et al. (2003) A randomized trial on the efficacy of intensive rehabilitation in the acute phase of ischemic stroke. J Neurol 250: 1206-1208.
444. Fang Y, Chen X, Lin H, Lin J, Huang R, et al. (2003) A study on additional early physiotherapy after stroke and factors affecting functional recovery. Clin Rehabil 17: 608-617.
445. Rodgers H, Mackintosh J, Price C, Wood R, McNamee P, et al. (2003) Does an early increased-intensity interdisciplinary upper limb therapy programme following acute stroke improve outcome? Clin Rehabil 17: 579-589.
446. Glasgow Augmented Physiotherapy Study (GAPS) (2004) Can augmented physiotherapy input enhance recovery of mobility after stroke? A randomized controlled trial. Clin Rehabil 18: 529-537.
447. Davidson I, Hillier V, Waters K, Walton T, Booth J (2005) A study to assess the effect of nursing interventions at the weekend for people with stroke. Clin Rehabil 19: 126-137.
448. Platz T, Eickhof C, van KS, Engel U, Pinkowski C, et al. (2005) Impairment-oriented training or Bobath therapy for severe arm paresis after stroke: a single-blind, multicentre randomized controlled trial. ClinRehabil 19: 714-724.
449. Katz-Leurer M, Sender I, Keren O, Dvir Z (2006) The influence of early cycling training on balance in stroke patients at the subacute stage. Results of a preliminary trial. Clin Rehabil 20: 398-405.
450. Langhammer B, Lindmark B, Stanghelle J (2007) Stroke patients and long-term training: is it worthwhile? A randomized comparison of two different training strategies after rehabilitation. Clin Rehabil 21: 495-510.
451. Huijgen B, Vollenbroek-Hutten M, Zampolini M, Opisso E, Bernabeu M, et al. (2008) Feasibility of a home-based telerehabilitation system compared to usual care: arm/hand function in patients with stroke, traumatic brain injury and multiple sclerosis. J Telemed Telecare 14: 249-256.
452. Harris J, Eng J, Miller W, Dawson A (2009) A self-administered Graded Repetitive Arm Supplementary Program (GRASP) improves arm function during inpatient stroke rehabilitation: a multi-site randomized controlled trial. Stroke 40: 2123-2128.
453. Harrington R, Taylor G, Hollinghurst S, Reed M, Kay H, et al. (2010) A community-based exercise and education scheme for stroke survivors: a randomized controlled trial and economic evaluation. Clin Rehabil 24: 3-15.
454. Hesse S, Welz A, Werner C, Quentin B, Wissel J (2011) Comparison of an intermittent high-intensity vs continuous low-intensity physiotherapy service over 12 months in community-dwelling people with stroke: a randomized trial. Clin Rehabil 25: 146-156.
455. Gelber D, Josefczyk P, Herman D, Good D, Verhulst S (1995) Comparison of two therapy approaches in the rehabilitation of the pure motor hemiparetic stroke patient. Neurorehabil Neural Repair 9: 191-196.

456. Langhammer B, Stanghelle J (2000) Bobath or motor relearning programme? A comparison of two different approaches of physiotherapy in stroke rehabilitation: a randomized controlled study. Clin Rehabil 14: 361-369.
457. Langhammer B, Stanghelle J (2003) Bobath or motor relearning programme? A follow-up one and four years post stroke. Clin Rehabil 17: 731-734.
458. Richards C, Malouin F, Bravo G, Dumas F, Wood-Dauphinee S (2004) The role of technology in task-oriented training in persons with subacute stroke: a randomized controlled trial. Neurorehabil Neural Repair 18: 199-211.
459. Tang Q, Yang Q, Wu Y, Wang G, Huang Z, et al. (2005) Effects of problem-oriented willed-movement therapy on motor abilities for people with poststroke cognitive deficits. Phys Ther 85: 1020-1033.
460. Van Vliet P, Lincoln N, Foxall A (2005) Comparison of Bobath based and movement science based treatment for stroke: a randomised controlled trial. J Neurol Neurosurg Psychiatry 76: 503-508.
461. Wang R, Chen H, Chen C, Yang Y (2005) Efficacy of Bobath versus orthopaedic approach on impairment and function at different motor recovery stages after stroke: a randomized controlled study. Clin Rehabil 19: 155-164.
462. Brock K, Haase G, Rothacher G, Cotton S (2011) Does physiotherapy based on the Bobath concept, in conjunction with a task practice, achieve greater improvement in walking ability in people with stroke compared to physiotherapy focused on structured task practice alone?: a pilot randomized controlled trial. Clin Rehabil 25: 903-912.
463. Bütefisch C, Hummelsheim H, Denzler P, Mauritz K (1995) Repetitive training of isolated movements improves the outcome of motor rehabilitation of the centrally paretic hand. J Neurol Sci 130: 59-68.
464. Dechaumont-Palacin S, Marque P, de Boissezon X, Castel-Lacanal E, Carel C, et al. (2008) Neural correlates of proprioceptive integration in the contralesional hemisphere of very impaired patients shortly after a subcortical stroke: an FMRI study. Neurorehabil Neural Repair 22: 154-165.
